# Formylation or methylation: what determines the chemoselectivity of the reaction of amine, CO_2_, and hydrosilane catalyzed by 1,3,2-diazaphospholene?†
†Electronic supplementary information (ESI) available: Additional computational results, energies, and Cartesian coordinates of the optimized structures. See DOI: 10.1039/c7sc00824d


**DOI:** 10.1039/c7sc00824d

**Published:** 2017-09-11

**Authors:** Yu Lu, Zhong-Hua Gao, Xiang-Yu Chen, Jiandong Guo, Zheyuan Liu, Yanfeng Dang, Song Ye, Zhi-Xiang Wang

**Affiliations:** a School of Chemistry and Chemical Engineering , University of the Chinese Academy of Sciences , Beijing 100049 , China . Email: zxwang@ucas.ac.cn; b Institute of Chemistry , Chinese Academy of Sciences , Beijing , 100190 , China . Email: songye@iccas.ac.cn

## Abstract

DFT computations have been performed to gain insight into the mechanisms of formylation/methylation of amines (*e.g.* methylaniline (**1a**)/2,2,4,4-tetramethylpiperidine (**2a**)) with CO_2_ and hydrosilane ([Si]H_2_, [Si] = Ph_2_Si), catalyzed by 1,3,2-diazaphospholene (**[NHP]**H). Different from the generally proposed sequential mechanism for the methylation of amine with CO_2_, *i.e.* methylation proceeds *via* formylation, followed by further reduction of formamide to give an *N*-methylated amine, the study characterized a competition mechanism between formylation and methylation. The chemoselectivity originates from the competition between the amine and **[NHP]**H hydride to attack the formyloxy carbon of [Si](OCHO)_2_ (the insertion product of CO_2_ into [Si]H_2_). When the attack of an amine (*e.g.***1a**) wins, the transformation affords formamide (**1b**) but would otherwise (*e.g.***2a**) result in an *N*-methylated amine (**2c**). The reduction of formamide by [Si]H_2_ or **[NHP]**H is highly unfavorable kinetically, thus we call attention to the sequential mechanism for understanding the methylation of amine with CO_2_. In addition, the study has the following key mechanistic findings. The activation of CO_2_ by **[NHP]**H establishes an equilibrium: **[NHP]**H + CO_2_ ⇄ **[NHP]**OCHO ⇄ **[NHP]^+^** + HCO_2_^–^. The ions play catalytic roles to promote formylation *via* HCO_2_^–^ or methylation *via***[NHP]^+^**. In **1a** formylation, HCO_2_^–^ initiates the reaction, giving **1b** and silanol byproducts. However, after the initiation, the silanol byproducts acting as hydrogen transfer shuttles are more effective than HCO_2_^–^ to promote formylation. In **2a** methylation, **[NHP]^+^** promotes the generation of the key species, formaldehyde and a carbocation species (**IM17^+^**). Our experimental study corroborates our computed mechanisms.

## Abstract 2

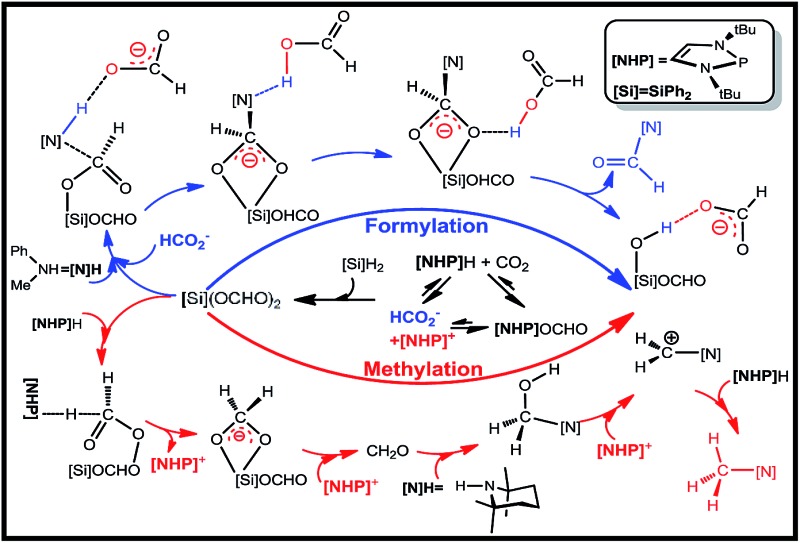
A DFT study demonstrates that methylation and formylation of amines with CO_2_ and hydrosilane, catalyzed by 1,3,2-diazaphospholene, are two competitive reaction channels.

## 1. Introduction

The rising concentration of carbon dioxide (CO_2_) in the atmosphere is one of the key factors for global warming, leading to great efforts to develop effective catalytic routes that convert CO_2_ to value-added chemicals.^1^–^3^ Formylation and methylation of amines with CO_2_ are promising synthetic strategies to use CO_2_ as a C1 carbon source.^4^ In 1998, Vaska and coworkers developed the first Pt-catalyzed formylation of amine with CO_2_ and H_2_.^5^ This study has encouraged further developments using other transition metal catalysts^6^ or metal-free catalysts.^7^ In 2012, Cantat and coworkers achieved the first organocatalytic formylation of amines with CO_2_ and hydrosilane, catalyzed by triazabicyclodecene (TBD).^8^ Since then, more similar transformations were reported.^9^ In 2013, Beller and coworkers reported the first methylation of amine with CO_2_ and hydrosilane, catalyzed by a ruthenium complex.^10^ More similar transformations were later developed.^11^ It is worth mentioning that Cantat *et al.* also developed metal-free methylation of CO_2_ with amines.^12^ Furthermore, transition metal catalyzed methylation of amines with CO_2_ and H_2_ has also been accomplished by several groups.^13^

**Scheme 1:**
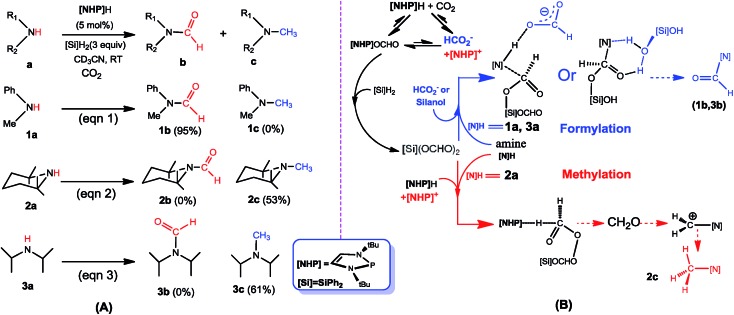
(A) Formylation (eqn (1)) and methylation (eqn (2) and (3)) of amines with CO_2_ and hydrosilane ([Si]H_2_ = Ph_2_SiH_2_), reported by Kinjo *et al.* (B) Schematic illustration of our proposed mechanism.

Previously, we studied the catalytic mechanisms of CO_2_ reduction to methanol^14^ and methane.^15^ In this context, we were intrigued by the catalytic reactions developed by Kinjo and coworkers.^16^ They used 1,3,2-diazaphospholene (**[NHP]**H) to catalyze the formylation of amines (**[N]**H) with CO_2_ and hydrosilane (Ph_2_SiH_2_ = [Si]H_2_) (*e.g.* eqn (1) in Scheme 1). Interestingly, two amines (**2a** and **3a**) were found to be exceptional, affording *N*-methylated amines (**2c** and **3c**). They attributed **2c** and **3c** to the further reductions of **2b** and **3b**, respectively, complying with the general consideration that methylation takes place sequentially through formylation, giving formamide, followed by the reduction of formamide.^10^,^17^ Nevertheless, we conceived that this mechanism may not be true in the present system. First, due to the smaller steric effect of **1b** compared to **2b**, **1b** should be reduced more easily than **2b**, but eqn (1) affords **1b** rather than **1c**. Second, if the methylation mechanism is true, *N*-methylated amines could be at least detected in eqn (1). In addition, Cantat *et al.*^18^ showed that in the TBD-catalyzed aminal synthesis from amine, CO_2_, and hydrosilane, which is somewhat similar to methylation, the formation of an aminal product takes place after forming [Si]OCH_2_O[Si] *via* two sequential 2-electron reductions of CO_2_ with hydrosilane and the HC(=O)O[Si] intermediate resulting from the first 2-electron reduction of CO_2_ with hydrosilane does not react with amine to give formamide. Thus, the formation of aminal does not pass formamide as an intermediate. Given these analyses, we carried out a DFT mechanistic study to deeply understand the catalytic system, in combination with experimental verifications. To our knowledge, there has been no systematic study on the mechanisms of formylation and methylation of amines with CO_2_, although Cantat and coworkers reported some computational results in their experimental study.^19^

Scheme 1B sketches our computed mechanisms. CO_2_ first inserts into the P–H bond of **[NHP]**H, giving **[NHP]**OCHO. The insertion is only slightly exergonic and the insertion product can easily dissociate into HCO_2_^–^ and **[NHP]^+^** ions, thus resulting in a microscopic equilibrium: **[NHP]**H + CO_2_ ⇄ **[NHP]**OCHO ⇄ **[NHP]^+^** + HCO_2_^–^. Subsequently, **[NHP]**OCHO reacts with [Si]H_2_, giving [Si](OCHO)_2_. Finally, [Si](OCHO)_2_ reacts with amine, giving either a formamide or an *N*-methylated amine, with the chemoselectivity controlled by the competition between the amine nucleophilic attack (blue pathway) and **[NHP]**H hydride transfer (red pathway). For small amines such as **1a**, the blue pathway is preferred, giving formamide (*e.g.***1b**) under the catalytic effect of HCO_2_^–^ or silanol (*e.g.* [Si](OH)_2_). For bulky amines (*e.g.***2a**), the red pathway is favored, giving the *N*-methylated amine (*e.g.***2c**) with the involvement of [**NHP**]H and **[NHP]^+^**. Instead of formamide being the intermediate of methylation, formaldehyde and a carbocation species were found to be the key intermediates of the methylation. Note that our results show that **3a** prefers formylation, giving **3b** rather than **3c**, as reported previously (eqn (3)).

## 2. Computational details

Experimentally, the reactions were carried out in a polar solvent (acetonitrile, *ε* = 35.7). Considering the possible significant effects of the strong polar solvent, all geometries were optimized and characterized as minima (no imaginary frequency) or transition states (TSs, having one unique imaginary frequency) at the M06-2X^20^/6-31G(d,p) level with the solvation effect of acetonitrile simulated by the SMD^21^ solvent model. At the M06-2X/6-31G(d,p) geometries, the energies were further refined by M06-2X/6-311++G(d,p) single-point energy calculations with the solvent effect accounted for by the SMD solvent model. All DFT calculations adopted ultrafine integration grids (Int = ultrafine) to ensure stable numerical integrations. The M06-2X/6-31G(d,p) frequencies were used for thermal and entropic corrections at 298.15 K and 1 atm. It should be emphasized that such a correction approach is based on the ideal gas phase model, which inevitably overestimates entropy contributions to free energies for reactions in solvent, in particular for reactions involving a multicomponent change, because they ignore the suppressing effect of solvent on the rotational and transitional freedoms of substrates. The entropy overestimation of the approach was also demonstrated experimentally.^22^,^23^ While no standard quantum mechanics-based method is available to accurately calculate entropy in solution, approximate methods were proposed. According to the proposal of Martin *et al.*^24^ we previously applied a correction of (*n* – *m*) × 4.3 kcal mol^–1^ for a process from *m*- to *n*-components and found that such corrected free energies were more reasonable than enthalpies and uncorrected free energies,^15^,^25^ although the protocol is by no means accurate. Other correction factors (*e.g.* 1.9,^26^ 2.6,3a,^27^ and 5.4 kcal mol^–1^ (ref. 28)) were adopted in the literature depending on the approximate approaches. As will be seen, our studied reactions involve multicomponent changes. As a conservative consideration, we applied a correction factor of 1.9 kcal mol^–1^ in this study. The corrected free energies are discussed and the uncorrected ones are given in the parentheses for references, unless otherwise specified. Note that using a correction factor of 4.3 kcal mol^–1^ does not alter our conclusions except for the numerical values. Natural bond orbital (NBO) analyses were performed at the M06-2X/6-311++G(d,p) level to assign partial atomic charges (*Q*).^29^ All calculations were carried out using Gaussian 09.^30^

## 3. Results and discussion

In this study, we use eqn (1) as a representative to compute the formylation mechanism of amine **1a** (Section 3.1). In Section 3.2, using eqn (2), we investigate the methylation mechanism of amine (**2a**). After characterizing the mechanisms of formylation and methylation, we discuss the origins of chemoselectivity and experimentally verify our proposed mechanism in Section 3.3. Our computed mechanisms involve ionic species, thus we explicitly label the charges of all species when applicable for simplicity of the descriptions.

### 3.1 Mechanism for **1a** formylation (eqn (1))

The catalytic cycle for **1a** formylation (eqn (1)) consists of three stages, namely, hydrophosphination of CO_2_ (stage I), formation of diformyloxysilane (stage II), and C–N bond formation (stage III). We below characterize how these stages proceed in order.

#### Hydrophosphination of CO_2_ (stage I)

**Fig. 1:**
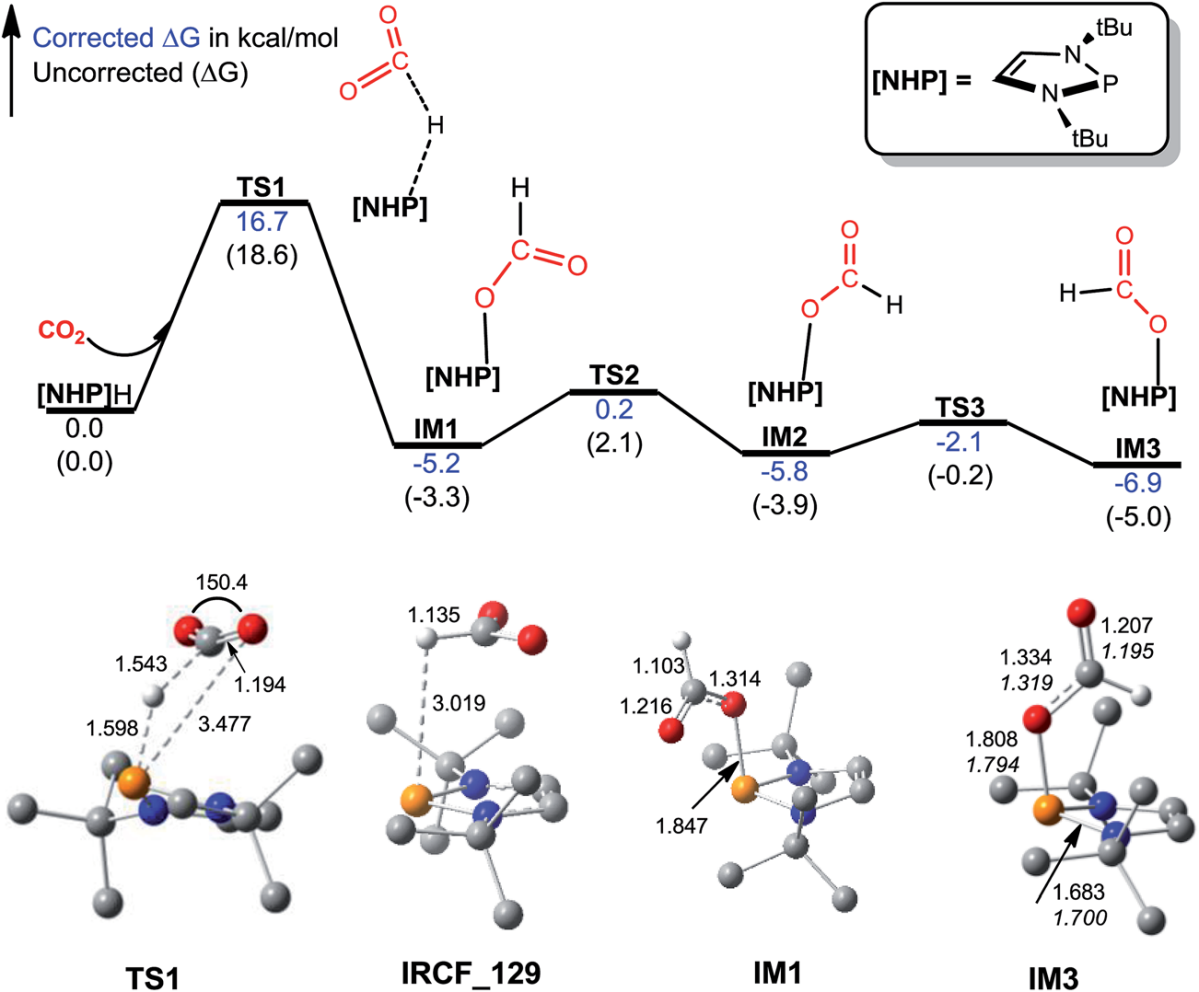
Free energy profile for hydrophosphination of CO_2_, together with key optimized structures with key bond lengths in angstroms and bond angles in degrees. All optimized structures are displayed in Fig. S2.† The italic values in **IM3** are X-ray geometric parameters.

Fig. 1 illustrates the mechanism for CO_2_ hydrophosphination, along with the key optimized structures. The catalyst **[NHP]**H is a hydride with P and H bearing 0.921 and –0.069*e* partial charges, respectively. Conventionally, CO_2_ prefers inserting into an E–H bond (*e.g.* E = B or Ni) *via* a four-membered TS, forming C–H and E–O bonds concertedly.14b,^15^ However, the optimized structure of **TS1** targeting for an insertion TS describes a hydrogen abstraction process. Zhu *et al.* reported a similar TS.^31^ The IRC (intrinsic reaction coordinate) calculation toward the product stopped after 129 steps (Fig. S1†), giving a structure (namely, **IRCF-129**) which can be viewed as an ion pair resulting from CO_2_ abstraction of the H^*δ*–^ atom of **[NHP]**H. However, geometric optimization starting from **IRCF-129** reached an insertion product **[NHP]**OCHO (**IM1**). We attribute the abnormal insertion to the difference between the P^*δ*+^–H^*δ*–^ bond in **[NHP]**H and E^*δ*+^–H^*δ*–^ bond (*e.g.* B–H or Ni–H);14b,^15^ the P center has a lone pair disfavoring P–O bond formation, while the E center features an empty orbital favoring E–O bond formation. **IM1** is different from the X-ray structure of the CO_2_ hydrophosphination product (**IM3**) but can convert to the more stable **IM3** easily (see Fig. 1). Overall, the insertion crosses a barrier of 16.7 kcal mol^–1^ and is exergonic by 6.9 kcal mol^–1^, indicating the feasibility of the process.

**Scheme 2:**
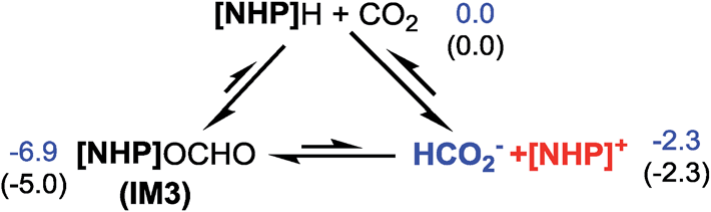
Microscopic equilibrium in the system. Values are relative free energies.

Kinjo *et al.* observed zwitterionic character of **IM3**. Consistently, the **[NHP]** and HCO_2_ moieties in **IM3** bear charges of 0.658 and –0.658*e*, respectively. Because of the zwitterionic nature, we conceived that **IM3** can dissociate easily in the strong polar acetonitrile solvent, as demonstrated by the small dissociation energy (4.6 kcal mol^–1^, see Scheme 2). Thus a microscopic equilibrium is expected in this catalytic system. As will be shown, the free **[NHP]^+^** and HCO_2_^–^ ions play catalytic roles to mediate subsequent steps of the transformation.

#### Formation of diformyloxysilane[Si](OCHO)_2_ (stage II)

**Fig. 2:**
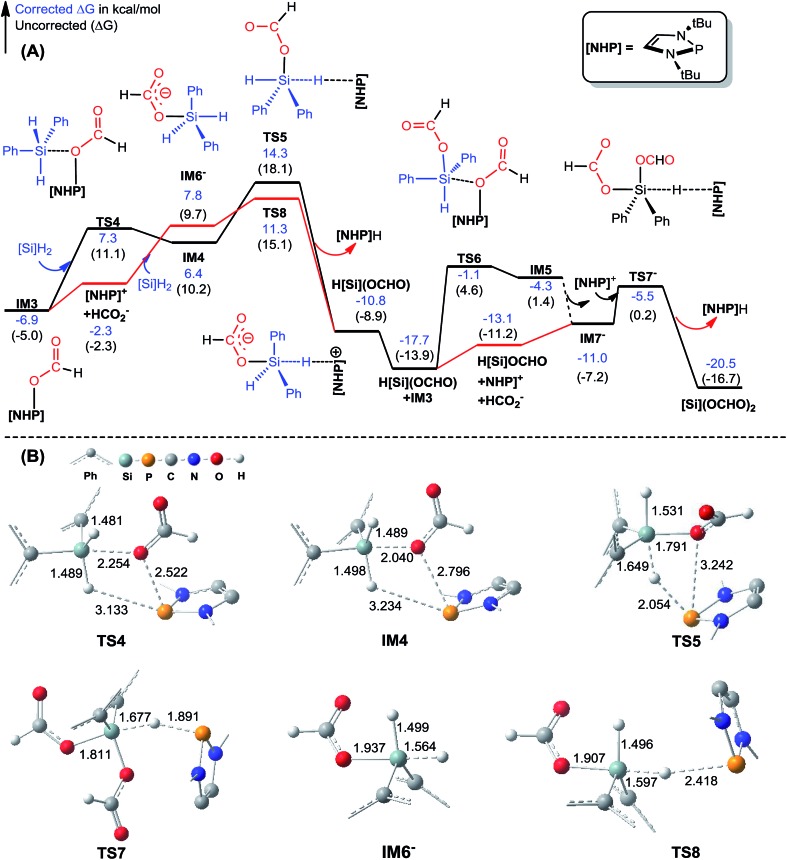
(A) Free energy profiles for the formation of [Si](OCHO)_2_. Energies are relative to **[NHP]**H, CO_2_, and [Si]H_2_ and are mass balanced. (B) Key optimized structures with key bond lengths given in angstroms. Other optimized structures are given in Fig. S4.† The details for the isomerization of **IM5** to **IM7^–^** are given in Fig. S3.†

Experimentally, it has been demonstrated that [Si](OCHO)_2_ is involved in the transformation.^16^Fig. 2 illustrates the possible pathways leading to [Si](OCHO)_2_, along with the key optimized structures. The black pathway from **IM3** to H[Si]OCHO in Fig. 2A can be considered as a stepwise σ-bond metathesis between **IM3** and [Si]H_2_, which forms Si–O and P–H bonds and meanwhile breaks Si–H and P–O bonds, leading to H[Si]OCHO and **[NHP]**H. When we attempted to locate a similar metathesis pathway leading H[Si]OCHO to [Si](OCHO)_2_, we were able to obtain a TS (*i.e.***TS6**) similar to **TS4** but the counterpart of **TS5** could not be located. **TS6** leads to an intermediate **IM5** tending to dissociate, giving **[NHP]^+^** and an anionic component which can isomerize to **IM7^–^** easily (the details for the isomerization are given in Fig. S3†). Subsequently, **[NHP]^+^** extracts the H(–Si) atom in **IM7^–^***via***TS7^–^**, giving [Si](OCHO)_2_ and regenerating the catalyst **[NHP]**H. The metathesis process from **IM3** to H[Si]OCHO is energetically feasible with a RDS (rate determining step) barrier of 21.2 kcal mol^–1^ (**TS5**) relative to **IM3**. Yet, we speculated that the stage may proceed *via* an ionic mechanism because free HCO_2_^–^ is available *via* the equilibrium (Scheme 2). The red pathway in Fig. 2A illustrates the ionic mechanism. Once **IM3** dissociates, the resulting HCO_2_^–^ attacks the Si^*δ*+^ center of [Si]H_2_, forming a HCO_2_^–^–[Si]H_2_ complex (**IM6^–^**). Although the nucleophilic attack is unfavorable by 10.2 kcal mol^–1^ mainly due to the entropic penalty of the association, HCO_2_^–^ activates its *trans* Si–H bond significantly, as reflected by the stretched Si–H bond (*R* = 1.564 Å in **IM6^–^***versus* 1.485 Å in [Si]H_2_). Subsequently, the cationic species **[NHP]^+^** extracts the activated H^*δ*–^ of the HCO_2_^–^–[Si]H_2_ complex (**IM6^–^**) *via* a S_N_2-like transition state **TS8**, resulting in H[Si]OCHO and regenerating **[NHP]**H. Comparing the two mechanisms, the ionic mechanism is 3.0 kcal mol^–1^ (the energy difference of **TS5** and **TS8**) kinetically more favorable than the metathesis mechanism. The lower **TS8** compared to **TS5** can be attributed to the more favorable *trans* Si–H bond activation by HCO_2_^–^ in **TS8**, compared to the *cis* activation in **TS5** (see Fig. 2B). The Si–H bond marked at 1.564 Å in **IM6^–^** is activated more significantly than that marked at 1.498 Å in **IM4**. Thus, the dissociation of **IM3** to free HCO_2_^–^ and **[NHP]^+^** essentially benefits the achievement of optimal *trans* activation of the Si–H bond in spite of the energy cost of 4.6 kcal mol^–1^ for the dissociation. For the conversion of H[Si]OCHO to [Si](OCHO)_2_, because HCO_2_^–^ as a free species can attack H[Si]OCHO directly, forming **IM7^–^**, a TS similar to **TS6** is not necessary. Overall, the transformation (2CO_2_ + [Si]H_2_ → [Si](OCHO)_2_) is exergonic by 20.5 kcal mol^–1^ and the RDS barrier is 18.2 kcal mol^–1^ (ionic mechanism) or 21.2 kcal mol^–1^ (metathesis mechanism), thus [Si](OCHO)_2_ can be produced easily, in agreement with the experimental observation.^16^

#### C–N bond formation (stage III)

**Scheme 3:**
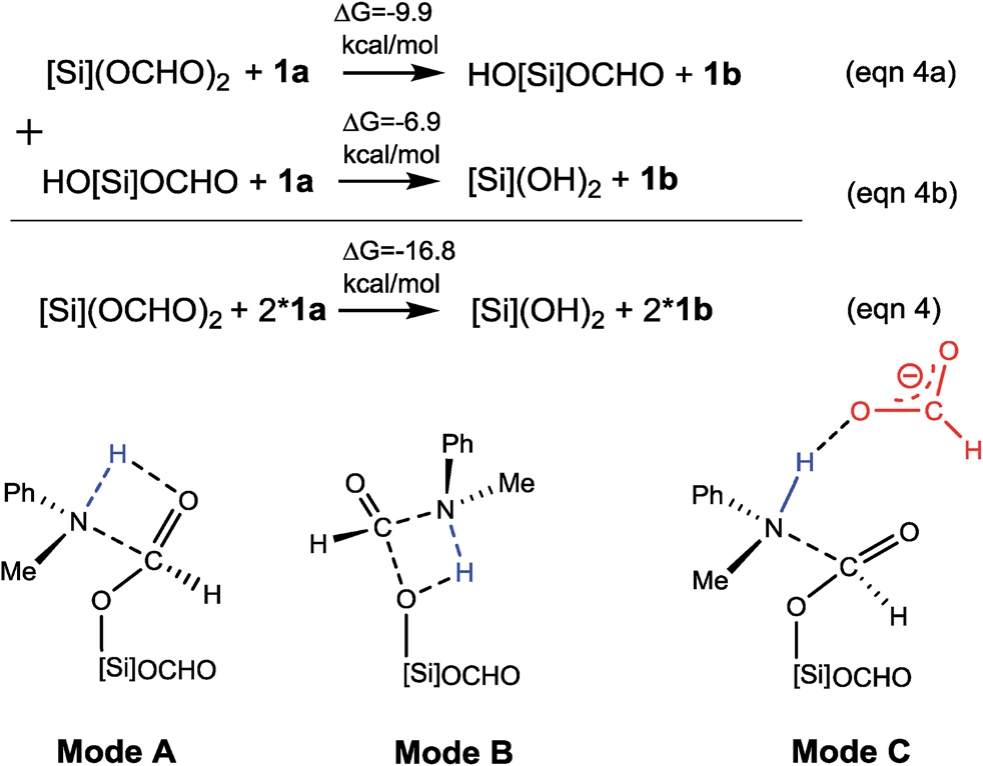
C–N bond formation stage (eqn (4)) and possible modes to form the bond.

After forming [Si](OCHO)_2_, a C–N bond starts to form (eqn (4) in Scheme 3). Intuitively, the bond can be formed *via* the nucleophilic attacks of amine, illustrated by mode A and B in Scheme 3, yet the high barriers, 41.1 (mode A) and 31.6 kcal mol^–1^ (mode B), rule out the two modes, considering that the reaction could occur under mild conditions (see eqn (1)). We explored other alternatives discussed below.

##### C–N bond formation catalyzed by HCO_2_^–^

**Fig. 3:**
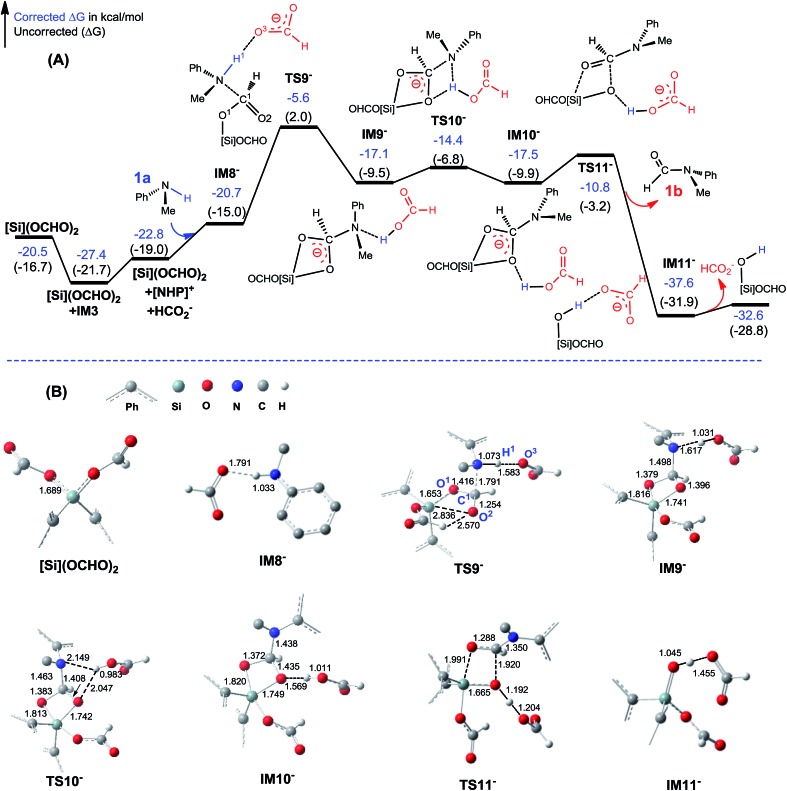
(A) Free energy profile for eqn (4a). Energies are relative to **[NHP]**H, CO_2_, **1a**, and [Si]H_2_ and are mass balanced. (B) Key optimized structures with key bond lengths in angstroms. Other optimized structures are given in Fig. S4.†

As discussed above, HCO_2_^–^ is available *via* microscopic equilibrium (Scheme 2). Thus, we considered whether a HCO_2_^–^ ion can facilitate the C–N bond formation *via* H-bonding to the N–H bond of **1a** (*i.e.* mode C in Scheme 3), because the bonding of the anionic species can enhance the nucleophilicity of amine **1a**. Fig. 3 depicts the mechanism for eqn (4a) under the catalytic effect of HCO_2_^–^, along with key optimized structures. First, HCO_2_^–^ and **1a** form a H-bond complex **IM8^–^**, then the complex attacks [Si](OCHO)_2_*via***TS9^–^**, giving **IM9^–^** with a C–N bond formed. Interestingly, the C–N bond formation shifts the N–H^1^···O^3^ H-bond pattern (R(N–H^1^)/R(H^1^···O^3^) = 1.033/1.791 Å) in **IM8^–^** to the N···H^1^–O^3^ pattern (R(N···H^1^)/R(H–O^3^) = 1.617/1.031 Å) in **IM9^–^**. Meanwhile, the formal negative charge of HCO_2_^–^ is shifted to the O^1^C^1^O^2^ moiety, as reflected by the bond equalization of the two C–O bonds from 1.348/1.198 Å in [Si](OCHO)_2_ to 1.379/1.396 Å in **IM9^–^**. The charge transfer shortens the O^2^···Si distance to 1.741 Å due to the attraction of Si^*δ*+^ and (O^2^)^*δ*–^ and elongates the Si–O^1^ bond (from 1.683 to 1.816 Å) because of the disruption of the original Si–O^1^ single bond, resulting in the four-membered ring (SiO^1^C^1^O^2^) in **IM9^–^**. Subsequently, the HCO_2_H moiety in **IM9^–^** swings to the O^2^ site by crossing a lower barrier (**TS10^–^**, 2.7 kcal mol^–1^ relative to **IM9^–^**), giving **IM10^–^**, in which the four-membered SiO^1^C^1^O^2^ ring and the O^2^···H^1^–O^3^ H-bond pattern (R(O^2^···H^1^)/R(H^1^–O^3^) = 1.569/1.011 Å) are maintained. **TS11^–^** leads **IM10^–^** to the formamide product (**1b**) and **IM11^–^**. In addition to breaking the C–O^2^ and Si–O^1^ bonds to give **1b**, **TS11^–^** alters the O^2^···H^1^–O^3^ H-bond pattern in **IM10^–^** to the O^2^–H^1^···O^3^ H-bond pattern (R(O^2^–H^1^)/R(H^1^···O^3^) = 1.045/1.455 Å) in **IM11^–^**. The dissociation of HCO_2_^–^ from **IM11^–^** to regenerate the active HCO_2_^–^ species costs only 5.0 kcal mol^–1^. The mechanism discussed above indicates that HCO_2_^–^ is not just a H-bond partner to enhance the nucleophilicity of amine **1a**. By altering the H-bond pattern between X···H–O and X–H···O (X = N or O) and shifting the charge between the HCO_2_^–^ and O^1^C^1^O^2^ unit, HCO_2_^–^ catalyzes bond formations (*i.e.* C–N and Si–O^2^ bonds in **IM9^–^**) and cleavages (*i.e.* C–O^2^ and Si–O^1^ bonds in **IM10^–^**). It is interesting that CO_2_ can be activated to an active species to facilitate its transformation. Following the same mechanism in Fig. 3, eqn (4b) takes place, producing another formamide (**1b**) and silanol [Si](OH)_2_. Without going into detail (see Fig. S5† for the energy profile of eqn (4b)), we mention that the RDS barrier of eqn (4b) is 27.3 kcal mol^–1^, 5.5 kcal mol^–1^ higher than that of eqn (4a).

##### C–N bond formation facilitated by hydrogen transfer shuttle

**Fig. 4:**
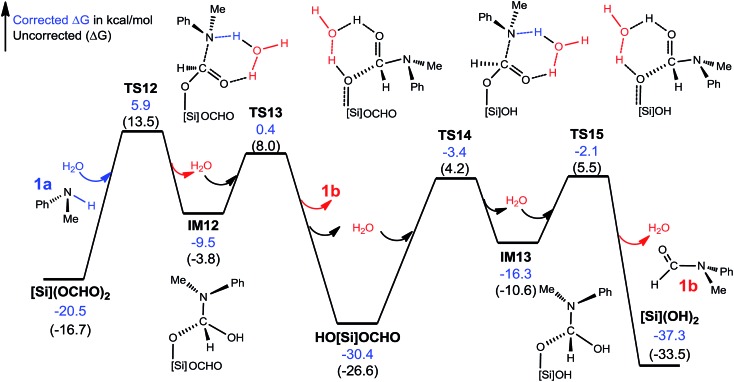
Free energy profile for the conversion of [Si](OCHO)_2_ + 2 × **1a**→ 2 × **1b** + [Si](OH)_2_. Optimized structures of key stationary points are displayed in Fig. S7.† Energies are relative to **[NHP]**H, CO_2_, **1a**, H_2_O, and [Si]H_2_ and are mass balanced.

The C–N bond formation through mode A and B involves a four-membered TS featuring hydrogen transfer (see Scheme 3). Thus a protic molecule such as water may act as a hydrogen transfer shuttle (H-shuttle)^32^,^33^ to facilitate the stage. In the present system, the possible H-shuttles could be water (trace water could not be excluded absolutely), *N*-methylaniline **1a**, and silanol (HO[Si]OCHO and [Si](OH)_2_), which are available when the reaction is initiated. Using water as a representative, we characterize the H-shuttle-aided pathway (eqn (4)) through mode A, as illustrated in Fig. 4. Without going into detail, we mention that the water-aided C–N bond formation involves two hydrogen transfer steps, sequentially forming C–N and breaking C–O (CO_2_ deoxygenation) bonds, as described by **TS12** and **TS13** for eqn (4a), respectively.

Table 1 compares the RDS barriers for eqn (4a) and (4b), mediated by various H-shuttles and HCO_2_^–^. Note that, because the hydrogen transfers do not involve **IM3** or **[NHP]^+^**/HCO_2_^–^ ions, their RDS barriers were measured relative to [Si](OCHO)_2_ for eqn (4a) or HO[Si](OCHO) for eqn (4b). As compared, water is a more effective H-shuttle than amine **1a**, which is consistent with our previous study of C–N bond formation in the dehydrogenative coupling of alcohol and amine.25*d* Both HO[Si]OCHO and [Si](OH)_2_ are better than water with HO[Si]OCHO being even better, which is due to the more polar O–H bond in silanol compared to that in water (see Fig. S6†). HCO_2_^–^ is more effective than water but less effective than silanol.

**Table 1:** Comparisons of the RDS barriers for eqn (4a) and (4b), facilitated by various promoters

Mediator	Eqn (4a)	Eqn (4b)
HCO_2_^–^	21.8(23.7)	27.3(29.2)
No (mode A)	41.1(43.0)	ND
Water (mode A)	26.4(30.2)	28.3(32.1)
Amine **1a** (mode A)^*a*^	28.7(32.5)	34.1(37.9)
HO[Si]OCHO (mode A)^*b*^	18.8(22.6)	19.9(23.7)
[Si](OH)_2_ (mode A)^*c*^	20.4(24.2)	24.8(28.6)
No (mode B)	31.6(33.5)	ND
Water (mode B)	30.5(34.3)	ND
HO[Si]OCHO (mode B)	27.3(31.1)	ND

^*a*^Complete pathway is given in Fig. S8.

^*b*^Complete pathway is given in Fig. S9.

^*c*^Complete pathway is given in Fig. S10. ND: not determined.

For the formation of the C–N bond through mode B (Scheme 3), the water H-shuttle does not help much with only a slightly lower barrier (30.5 kcal mol^–1^), compared to 31.6 kcal mol^–1^ without the H-shuttle. The most effective H-shuttle, HO[Si]OCHO, in the case of mode A has a barrier of 27.3 kcal mol^–1^ in the case of mode B, which is much higher than 18.8 kcal mol^–1^ through mode A. We thus do not expect that other H-shuttles could aid the stage through the mode B mechanism more efficiently than that through mode A and did not pursue the mode further.

After characterizing the efficiency of these hydrogen transfer mediators in prompting C–N bond formation, we now discuss how the C–N bond could actually be formed. Both eqn (4a) and (4b) are thermodynamically favorable, being exergonic by 9.9 and 6.9 kcal mol^–1^, respectively. We focus on the kinetics of the reactions using eqn (4a) as an example for simplicity.

It was reported that in the absence of **[NHP]**H and CO_2_, [Si](OCHO)_2_ alone could react with **1a** to give **1b**. As the efficiency of the reaction was not reported, our energetic results show that the reaction is able to take place, because the barrier for eqn (4a), when using water as a H-shuttle, is 26.4 kcal mol^–1^, which is somewhat high but in a reasonable range for a reaction to occur. Importantly, when the reaction is initiated to produce silanol, the silanol byproducts can promote the reaction more effectively, with lower barriers (see Table 1). In the presence of **[NHP]**H and CO_2_, HCO_2_^–^ plays the role of initiating the reaction rather than water, because the RDS barrier of 21.8 kcal mol^–1^ using HCO_2_^–^ as a catalyst is much lower than 26.4 kcal mol^–1^ using a water H-shuttle as a promoter. As the reaction proceeds, more and more silanols (HO[Si]OCHO or [Si](OH)_2_) are produced, thus, silanols take the role of HCO_2_^–^ to promote C–N bond formation.

### 3.2 Mechanism for **2a** methylation (eqn (2))

Kinjo *et al.*^16^ have applied an **[NHP]**H catalyst to perform formylations of a range of primary and secondary amines. Intriguingly, 2,2,4,4-tetramethylpiperidine (**2a**) and diisopropylamine (**3a**) were found to afford *N*-methylated amines, **2c** (eqn (2)) and **3c** (eqn (3)), respectively. In general, formamide (the formylation product) was considered to be the intermediate for the methylation of amine with CO_2_.^10^,^17^ The mechanism was also adopted to elucidate the methylation products (**2c** and **3c**). Nevertheless, we reasoned that this could not be true in the present catalytic system (*supra infra*). Using eqn (2) as an example, we investigate the methylation mechanism.

**Fig. 5:**
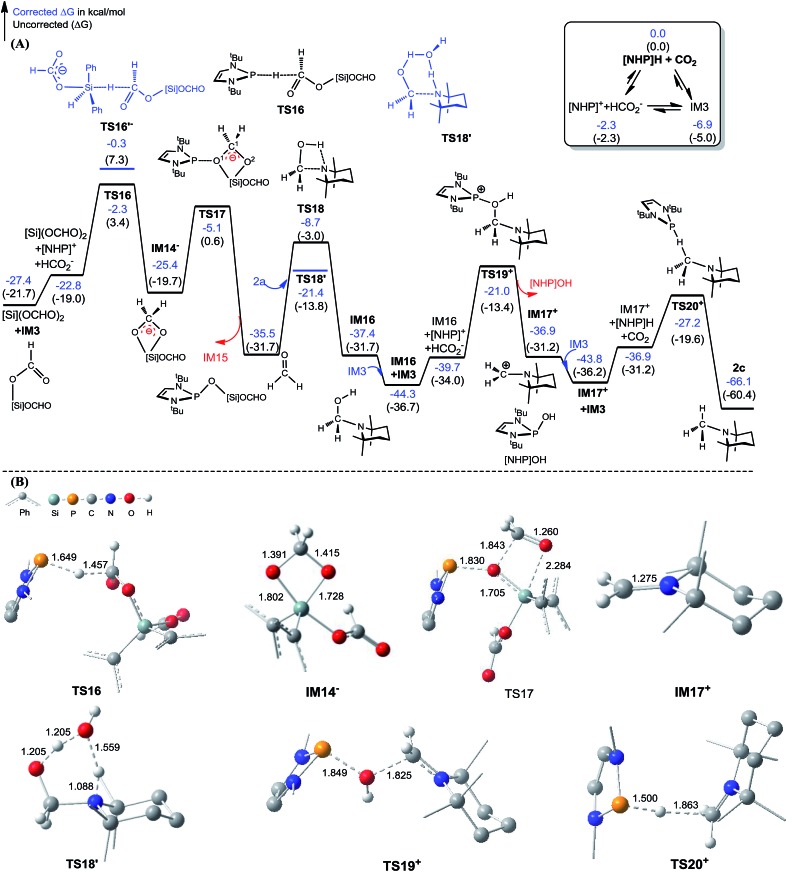
(A) Free energy profile for the methylation of [Si](OCHO)_2_ + **2a** → **2c** + H[Si]OCHO. (B) Optimized structures of key stationary points with key bond lengths given in angstroms. Those of others are given in Fig. S11.† Energies are relative to **[NHP]**H, CO_2_, **2a**, H_2_O, and [Si]H_2_ and are mass balanced.

The C–N bond in formylation is formed *via* the nucleophilic attack of amine (**1a**) to [Si](OCHO)_2_ (see **TS9^–^** in Fig. 3). Alternatively, we speculated that the hydrides, either [Si]H_2_ or **[NHP]**H, may compete with the amine to attack [Si](OCHO)_2_. Fig. 5 illustrates our computed pathway for **2a** methylation, along with key optimized structures. Starting from [Si](OCHO)_2_, **[NHP]**H first transfers its H^*δ*–^ to a formyloxy carbon of [Si](OCHO)_2_ with a barrier of 25.1 kcal mol^–1^ (**TS16**). Under the catalytic effect of HCO_2_^–^, [Si]H_2_ offers its H^*δ*–^ with the higher barrier (27.1 kcal mol^–1^ at **TS16′^–^**). Regardless of which hydride attacks [Si](OCHO)_2_, the hydride transfer results in an anionic four-membered intermediate **IM14^–^**, which corresponds to **IM9^–^** in Fig. 3. Subsequently, the **[NHP]^+^** cation attacks an O atom of the four-membered ring *via***TS17**, breaking the C^1^–O^1^ and Si–O^2^ bonds, resulting in formaldehyde (CH_2_O) and **[NHP]**O[Si]OCHO (**IM15**). The *in situ* generated CH_2_O then attacks **2a** electrophilically, forming a C–N bond and meanwhile transferring the (N–)H atom of amine to the carbonyl group of the formaldehyde moiety *via***TS18**, resulting in **IM16**. The barrier for the process is 26.8 kcal mol^–1^ (**TS18** relative to **IM15**), which is somewhat high but can be greatly lowered when a H-shuttle is used. For example, a water H-shuttle can lower the barrier to 14.1 kcal mol^–1^ (**TS18′**).

Subsequently, another **[NHP]^+^** attacks the hydroxyl group of **IM16***via***TS19^+^**, leading to a carbocation species (**IM17^+^**) and **[NHP]**OH with a barrier of 23.3 kcal mol^–1^ (**TS19^+^** relative to **IM16** + **IM3**). After receiving a H^*δ*–^ of **[NHP]**H or [Si]H_2_, the carbocation species converts to an *N*-methylated amine (**2c**). Our calculations showed that for this step, **[NHP]**H is a preferred hydride donor with a barrier of 16.6 kcal mol^–1^ (**TS20^+^** relative to **IM17^+^** + **IM3**). An attempt using HCO_2_^–^ to promote the H^*δ*–^ transfer of [Si]H_2_ was not successful, and the geometric optimization to locate the H^*δ*–^ transfer TS indicated that the steric effect between the bulky amine and [Si]H_2_ prevents the hydride transfer.

According to the methylation pathway (Fig. 5A), the reaction seems to consume the catalyst by forming **[NHP]**O[Si]OCHO (*i.e.***IM15**) and **[NHP]**OH by-products. However, as detailed in ESI 2,† the two intermediates can be recovered to catalyst **[NHP]**H feasibly in terms of both kinetics and thermodynamics.

The methylation mechanism involves formaldehyde and a carbocation species **IM17^+^** as the key intermediates. For the viability of formaldehyde, we call attention to the fact that Bontemps, Sabo-Etienne and coworkers experimentally detected formaldehyde in their Ru-catalyzed conversion of CO_2_ to C2 species with pinacolborane as a reducing reagent.^34^ Previously, we predicted that formaldehyde could be involved in the NHC- and Ni-catalyzed CO_2_ conversion to CH_3_OH.^14^ The involvement of a carbocation species in CO_2_ conversion has not ever been reported. For the viability of the carbocation species (**IM17^+^**), the cationic species must not form stable species (namely, **IM17OCHO**) with the anionic HCO_2_^–^, because a deep trap would raise the hydrogen transfer barrier from **IM17^+^** + **IM3** to **TS20^+^** (Fig. 5A). To estimate the stability of **IM17OCHO**, we computed the reaction energy of eqn (5). The small endergonicity (1.8 kcal mol^–1^) of the equation indicates that **IM17OCHO** is only slightly more stable than **IM3**.
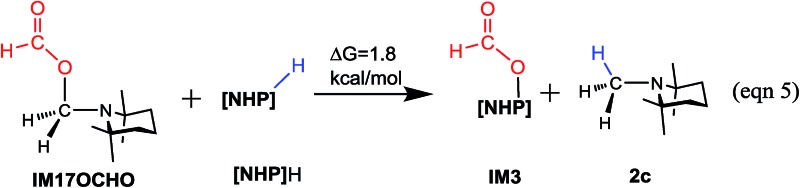


It is interesting to compare the roles of the **[NHP]^+^** and HCO_2_^–^ ions in formylation and methylation. In **1a** formylation (Fig. 3), only the HCO_2_^–^ component plays the catalytic role and **[NHP]^+^** is a spectator. Differently, in **2a** methylation (Fig. 5) the cationic component **[NHP]^+^** plays the catalytic role, and **[NHP]^+^** promotes the generation of CH_2_O (from **IM14^–^** to **IM15**) and the carbocation species (**IM17^+^**) from **IM16**.

### 3.3 The origins for chemoselectivities of formylation and methylation

**Fig. 6:**
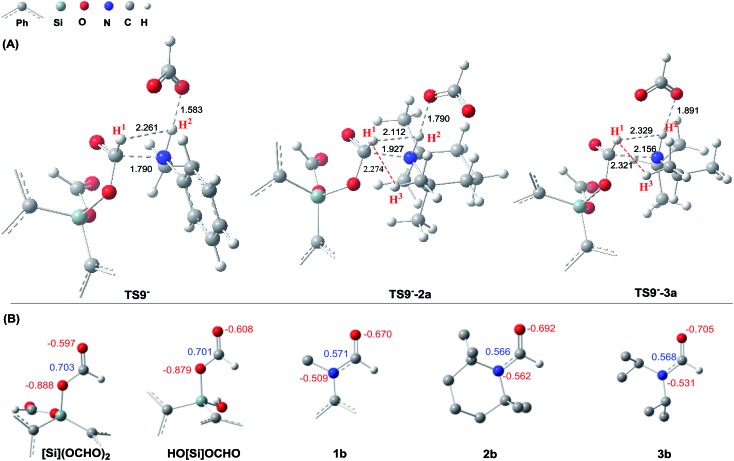
(A) Comparing the structures of the transition states (**TS9^–^**, **TS9^–^-2a**, and **TS9^–^-3a**) resulting in **1a**, **2a**, and **3a** formylations. (B) Comparing the NBO charges (in *e*) of [Si](OCHO)_2_ and HO[Si]OCHO with those of formamides (**1b–3b**).

The detailed characterizations of the mechanisms of eqn (1) and (2) facilitate our understanding of the chemoselectivities of the catalytic system. Using the conversion of the first formyloxy group of [Si](OCHO)_2_ as a representative case, we discuss the origins of the chemoselectivities. Key results for the conversion of the second formyloxy group of [Si](OCHO)_2_ (*i.e.* that in HO[Si]OCHO given in Table S1†) support the discussions below. According to the discussion in Section 3.2, the formylation/methylation preference stems from the competition between nucleophilic attacks of amine and hydride (*i.e.***TS9^–^** in Fig. 3 and **TS16** in Fig. 5) to [Si](OCHO)_2_. Table 2 compares the barriers of the two attacks for different amines. Note that the barrier for methylation is independent of amines. For **1a** formylation, the barrier is 21.8 kcal mol^–1^, which is well below the barrier of 25.1 kcal mol^–1^ for methylation, thus eqn (1) prefers formylation. In contrast, the barrier (29.3 kcal mol^–1^, **TS9-2a** in Fig. 6) for **2a** formylation is much higher than the barrier of 25.1 kcal mol^–1^ for its methylation, rationalizing the production of *N*-methylated amine (*i.e.***2c**) in eqn (2). The higher formylation barrier of **2a** compared to **1a** can be attributed to the greater steric effect in **TS9^–^-2a** than that in **TS9^–^**, as indicated by the shorter H^1^···H^2^ distance (2.112 Å) than that (2.261 Å) in **TS9^–^**. In addition, **TS9^–^-2a** suffers steric repulsion between H^1^ and H^3^.

**Table 2:** Comparisons of the barriers for formylation, methylation, and hydride transfer from **[NHP]**H and HCO_2_^–^–[Si]H_2_ to formamides^*a*^

Substrate		Formylation	Methylation		Hydride transfer to formamide		
			Hydride source		Hydride source		
			**[NHP]**H	HCO_2_^–^–[Si]H_2_		**[NHP]**H	HCO_2_^–^–[Si]H_2_
		Δ*G*^≠^	Δ*G*^≠^	Δ*G*^≠^		Δ*G*^≠^	Δ*G*^≠^
**1a**	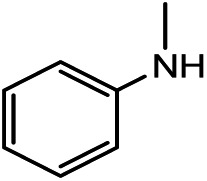	21.8(23.7)^*b*^	25.1(25.1)	27.1(29.0)	**1b**	37.3(37.3)	36.7(38.6)
**2a**	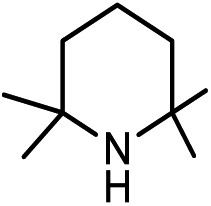	29.3(31.2)			**2b**	46.5(46.5)	41.8(43.7)
**3a**	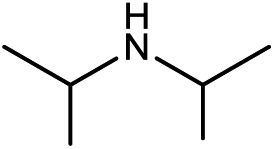	20.5(22.4)			**3b**	44.1(44.1)	43.6(45.5)
**4a**	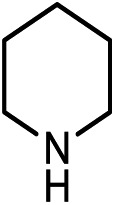	18.8(20.7)			**4b**	43.3(43.3)	43.7(45.6)
**5a**	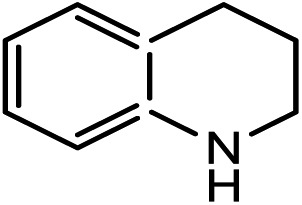	20.7(22.6)			**5b**	38.3(38.3)	36.5(38.4)
**6a**	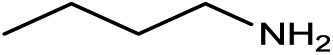	17.3(19.2)			**6b**	42.5(42.5)	39.8(41.7)
**7a**	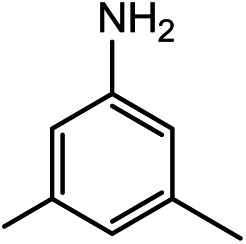	22.5(24.4)			**7b**	35.7(35.7)	34.6(36.5)

^*a*^All optimized structures of the transition states are displayed in Fig. S12.

^*b*^Values in parentheses are the free energy barriers without corrections.

The competition mechanisms rationalize the chemoselectivities of eqn (1) and (2), but the energetic results disagree with the reported experimental result of eqn (3), affording *N*-methylated amine **3c**. The formylation barrier of 20.5 kcal mol^–1^ (**TS9^–^-3a** in Fig. 6A) for **3a** is lower than that (25.1 kcal mol^–1^) for its methylation. On the other hand, comparing the structures of **TS9^–^-3a** and **TS9^–^** (the TSs for **3a** and **1a** formylations respectively), the H^1^–H^2^ distance (2.329 Å) in the former is even longer than that (2.261 Å) in the latter, indicating a smaller steric effect in **TS9^–^-3a** than in **TS9^–^**. In addition, the N atom in **3a** bears more negative charge (–0.728*e*) than that (–0.658*e*) in **1a**, indicating that **3a** is more nucleophilic than **1a**. Thus both the steric and electronic effect agree with the slightly lower formylation barrier (20.5 kcal mol^–1^) of **3a** than that of **1a** (21.8 kcal mol^–1^). We doubt that eqn (3) might actually produce formamide (**3b**).

**Scheme 4:**
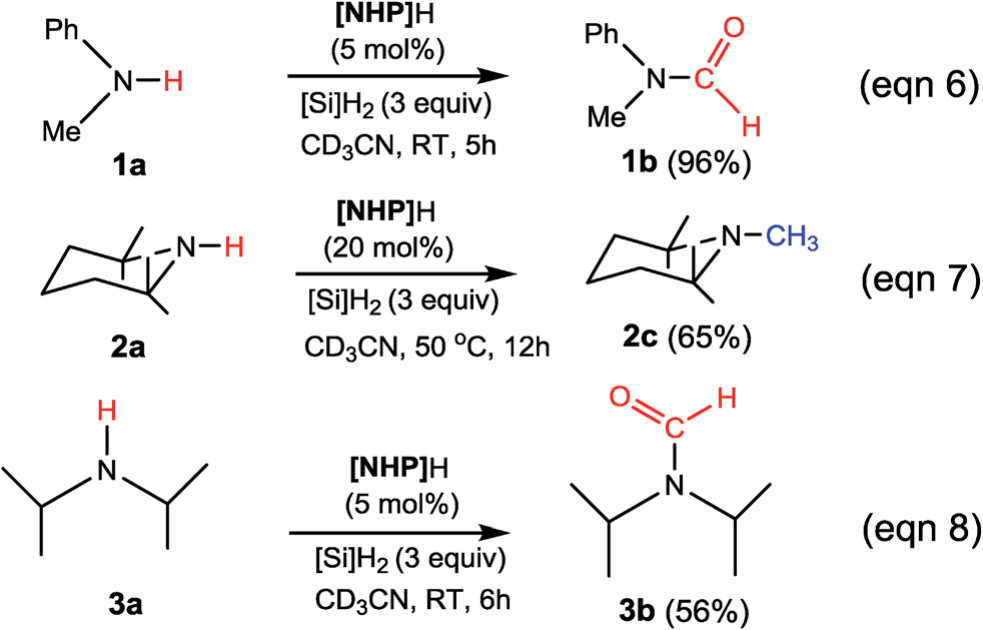
Our experimental results. See ESI 3 for experimental details.†

To verify our computed mechanisms and the production of **3b** in eqn (3), we performed experiments to study the reactions of **1a–3a** (see ESI 3 for experimental details†).^35^Scheme 4 shows our experimental results. Under the same experimental conditions, we were successful in reproducing the reported results of eqn (1), giving **1a** in 96% yield (see eqn (6)). However, our study shows that **3a** prefers to undergo formylation, affording formamide (**3b**) in 56% yield (eqn (8)), rather than *N*-methylated amine **3c** as reported previously (eqn (3)), supporting our computational prediction. For **2a**, under the same experimental conditions, we could only obtain traces of **2c**. Based on our computed mechanism, we reasoned that the poor performance of the reaction could be due to (a) the barrier for methylation (25.1 kcal mol^–1^) being higher than that for formylation (*e.g.* 21.8 kcal mol^–1^ for **1a** formylation) and (b) **[NHP]**H being required to finally reduce **IM17^+^** to **2c** (see Fig. 5), but it could be consumed during the process reaching **IM17^+^**. Thus, we modified the experimental conditions as shown in eqn (7) of Scheme 4. Delightedly, under the modified conditions, the methylated amine **2c** could be produced in 65% yield. Overall the experimental results corroborate our computational prediction satisfactorily.

We have shown that, in the present catalytic system, it is unlikely that methylation passes through formamide as an intermediate. We analyze why this is true. To further reduce formamide, the hydride (either **[NHP]**H or [Si]H_2_) should transfer its H^*δ*–^ to the carbonyl carbon of formamide, thus the electrophilicity of the carbon should be a factor to determine how favorably the formamide accepts a hydridic hydrogen of a hydride donor. Fig. 6B compares the NBO charges of formamides (**1b–3b**) with those of [Si](OCHO)_2_ and HO[Si]OCHO. It can be found that the formyloxy carbon in [Si](OCHO)_2_ and HO[Si]OCHO bears significantly more positive charge (>0.70*e*) than that in formamides (<0.58*e*). Thus [Si](OCHO)_2_ and HO[Si]OCHO can be reduced more easily than formamides. Consistently, the hydride transfer barriers from **[NHP]**H to **1b**, **2b**, and **3b** are substantially higher (37.3–44.1 kcal mol^–1^) than that (25.1 kcal mol^–1^) to [Si](OCHO)_2_. This is also true when [Si]H_2_ is used as the hydride donor with HCO_2_^–^ as the promoter (see Table 2).

To further corroborate our conclusions, we calculated the RDS barriers for formylation of the other four amines (**4a–7a** in Table 2) reported in ref. 16. The barriers for formylation of the four amines, ranging from 18.8–22.5 kcal mol^–1^, are all lower than the barrier for methylation (25.1 kcal mol^–1^), in excellent agreement with the experimental fact that these amines prefer formylation. Again, the barriers for hydride transfers to their corresponding formamides (**4b–5b**) are substantially high (>34.6 kcal mol^–1^). The high reduction barriers of formamides call attention to the sequential mechanism for understanding the methylation of amine with CO_2_.

## 4. Conclusions

In this study, we have performed a DFT study to investigate the catalytic mechanisms of the 1,3,2-diazaphospholene (**[NHP]**H)-mediated formylation/methylation of amines (methylaniline (**1a**)/2,2,4,4-tetramethylpiperidine (**2a**)) with CO_2_ and hydrosilane (Ph_2_SiH_2_ = [Si]H_2_) as a reducing reagent. Formylation of **1a** proceeds *via* three stages, including hydrophosphination of CO_2_, giving **[NHP]**OCHO (stage I), reaction of **[NHP]**OCHO with [Si]H_2_ to form [Si](OCHO)_2_ (stage II), and aminolysis of [Si](OCHO)_2_ to form a C–N bond, finally affording formamide (stage III). Methylation of **2a** shares the first two stages of formylation but is different in stage III. After stages I and II, the resultant [Si](OCHO)_2_ is preferentially subjected to the attack of an **[NPH]**H hydride, resulting in formaldehyde which then couples with **2a** to form a C–N bond in **IM16**. Subsequently, **IM16** converts to a carbocation species. The methyl group is finally formed *via* hydride transfer of **[NHP]**H to the carbocation species. Thus, different from the general consideration that methylation passes through formamide as reduced intermediates of CO_2_, the formylation and methylation in the present catalytic system are two competitive reaction channels. The chemoselectivity originates from the competition between amines and **[NHP]**H to attack the formyloxy carbon of [Si](OCHO)_2_. If the attack of an amine (*e.g.***1a**) wins the competition, the transformation affords formamide (**1b**) and otherwise (*e.g.***2a**) results in *N*-methylated amine (**2c**). The reduction of formamides is highly kinetically unfavorable, which calls attention to the sequential mechanism for understanding amine methylation with CO_2_.

On the basis of the detailed pathways, we have the following key findings in terms of reaction modes. The activation of CO_2_ by **[NHP]**H establishes a microscopic equilibrium: **[NHP]**H + CO_2_ ⇄ **[NHP]**OCHO ⇄ **[NHP]^+^** + HCO_2_^–^. The ions play catalytic roles to facilitate formylation with HCO_2_^–^ or methylation with **[NHP]^+^**. In **1a** formylation, HCO_2_^–^ initially forms a N–H···O (of HCO_2_^–^) H-bond complex with **1a** to attack [Si](OCHO)_2_. By altering the H-bond pattern between X–H···O and X···H–O (X = N or O) and shifting the formal charge between HCO_2_^–^ and the OCO unit in [Si](OCHO)_2_, HCO_2_^–^ promotes C–N bond formation and CO_2_ deoxygenation, finally resulting in formamide. However, it should be noted that, after the formylation is initiated, the silanol byproduct (either HO[Si]OCHO or [Si](OH)_2_) is more effective than HCO_2_^–^ to promote the formylation. Formaldehyde and a carbocation (**IM17^+^**) were characterized to be two important species to tunnel methylation and the generations of the species require the catalytic action of **[NHP]^+^**.

## Conflicts of interest

There are no conflicts to declare.

## Supplementary Material

Supplementary information
