# Supplementary material for: Formylation or methylation: what determines the chemoselectivity of the reaction of amine, CO2, and hydrosilane catalyzed by 1,3,2-diazaphospholene?
Source: Chem Sci. 2017 Sep 11;8(11):7637–50. doi: 10.1039/c7sc00824d (PMC5849201; doi:10.1039/c7sc00824d)
Supplement: Supplementary file 1 [file SC-008-C7SC00824D-s001.pdf]

Electronic Supplementary Material (ESI) for Chemical Science.
This journal is © The Royal Society of Chemistry 2017

Supporting Information

Formylation or methylation: what determine the chemoselectivity of the reaction of amine, CO_2, and hydrosilane, catalyzed by 1,3,2-diazaphospholene?

Yu Lu,[†, ǁ] Zhong-Hua Gao,[‡, ǁ] Xiang-Yu Chen,[‡] Jiandong Guo,[†] Zheyuan Liu,[†] Yanfeng, Dang, [†,] Song Ye*[,‡] and Zhi-Xiang Wang[*,†]

[†]School of Chemistry and Chemical Engineering, University of the Chinese Academy of Sciences, Beijing 100049, China (zxwang@ucas.ac.cn (ZXW)

[‡]Institute of Chemistry, Chinese Academy of Sciences, Beijing, 100190, China (songye@iccas.ac.cn (YS)

[ǁ] Y. L. and Z.-H. G. contributed equally to this work.

Table of Contents

SI 1: Figures S1–S12 and Table S1 .... S2–S12

SI 2: Regeneration of **[NHP]**H catalyst from **[NHP]**O[Si]OCHO and **[NHP]**OH S13–S14

SI 3: Experimental details and results S15–S22

SI 4: Cartesian coordinates, SCF energies, and free energies of optimized structures S23–S74

SI 1: Figures S1–S12 and Table S1

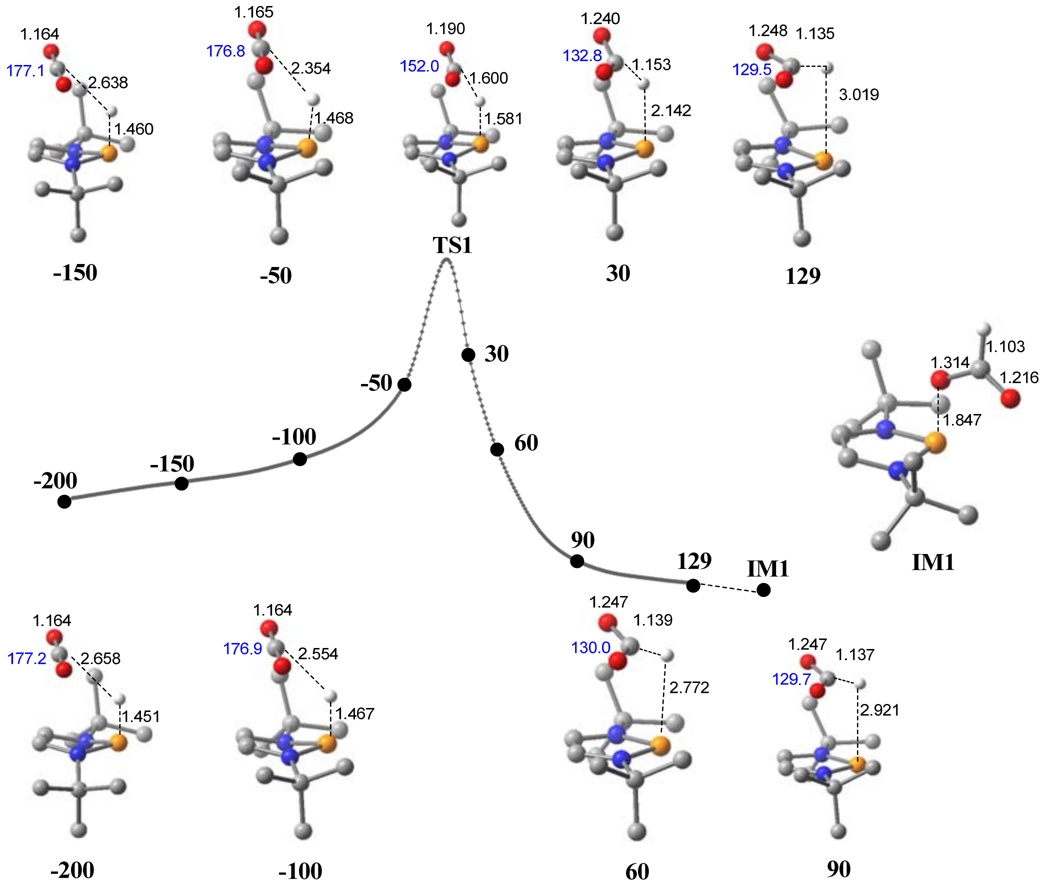

Figure S1. IRC results starting from **TS1.** In the backward direction, the IRC leads to reactants (CO_2 + **[NHP]**H). In the forward direction, IRC terminated after 129 steps. However, the geometric optimization starting at the point led to **IM1**. Values in black are key bond lengths in angstroms, values in blue are OCO bond angles in degrees. Trivial H atoms are omitted for clarity.

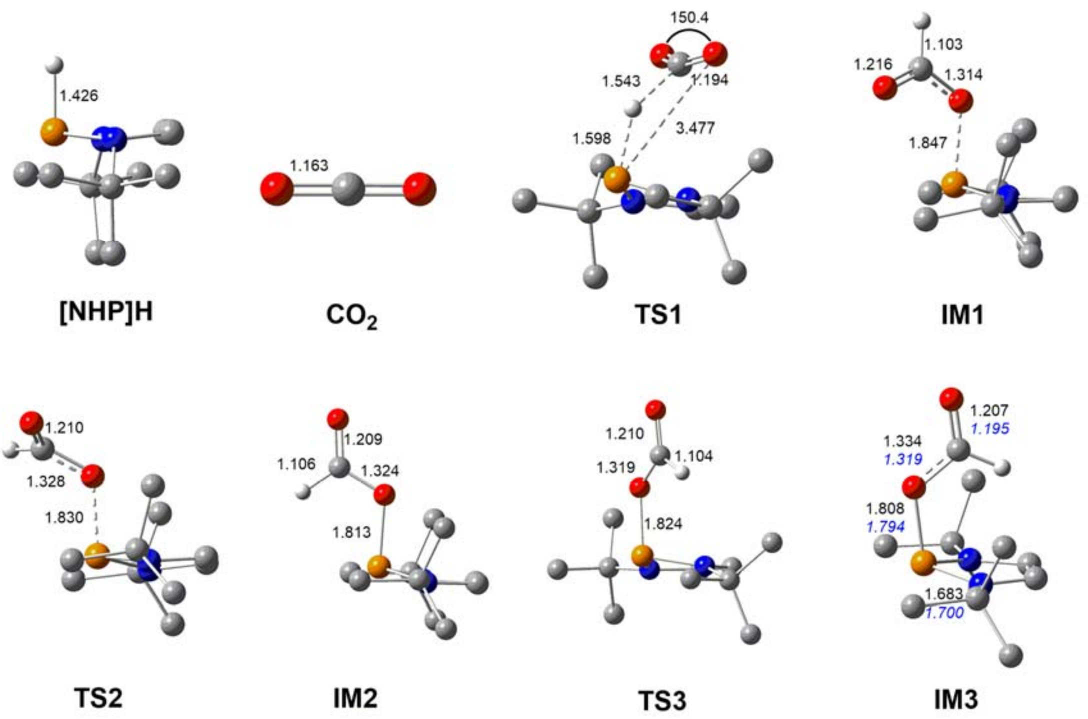

Figure S2. M06-2X/6-31G(d,p) optimized structures of stationary points shown in Figure 1, along with key bond lengths in angstroms. The italic values in **IM3** are X-ray geometric parameters. Trivial H atoms are omitted for clarity.

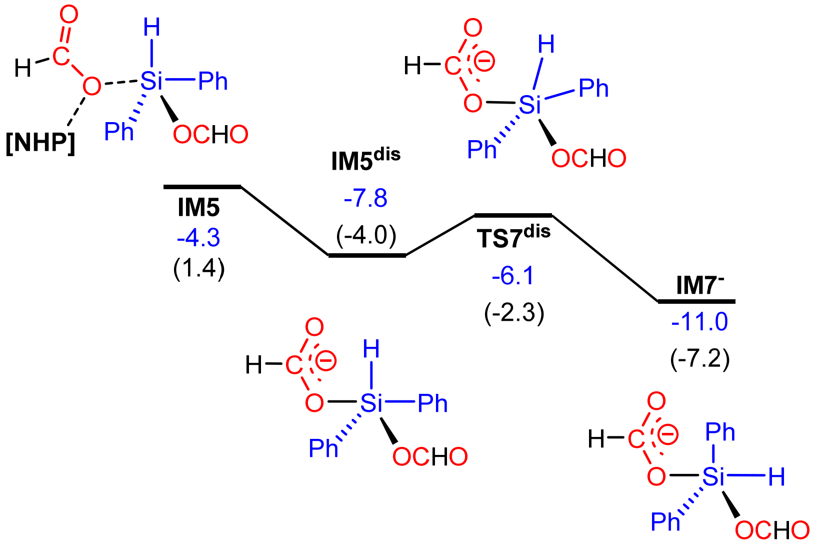

Figure S3. Free energy profile for the isomerization of **IM5** to **IM7**$^-$. Energies are relative to **[NHP]**H, [Si]H_2, and CO_2, and are mass balanced.

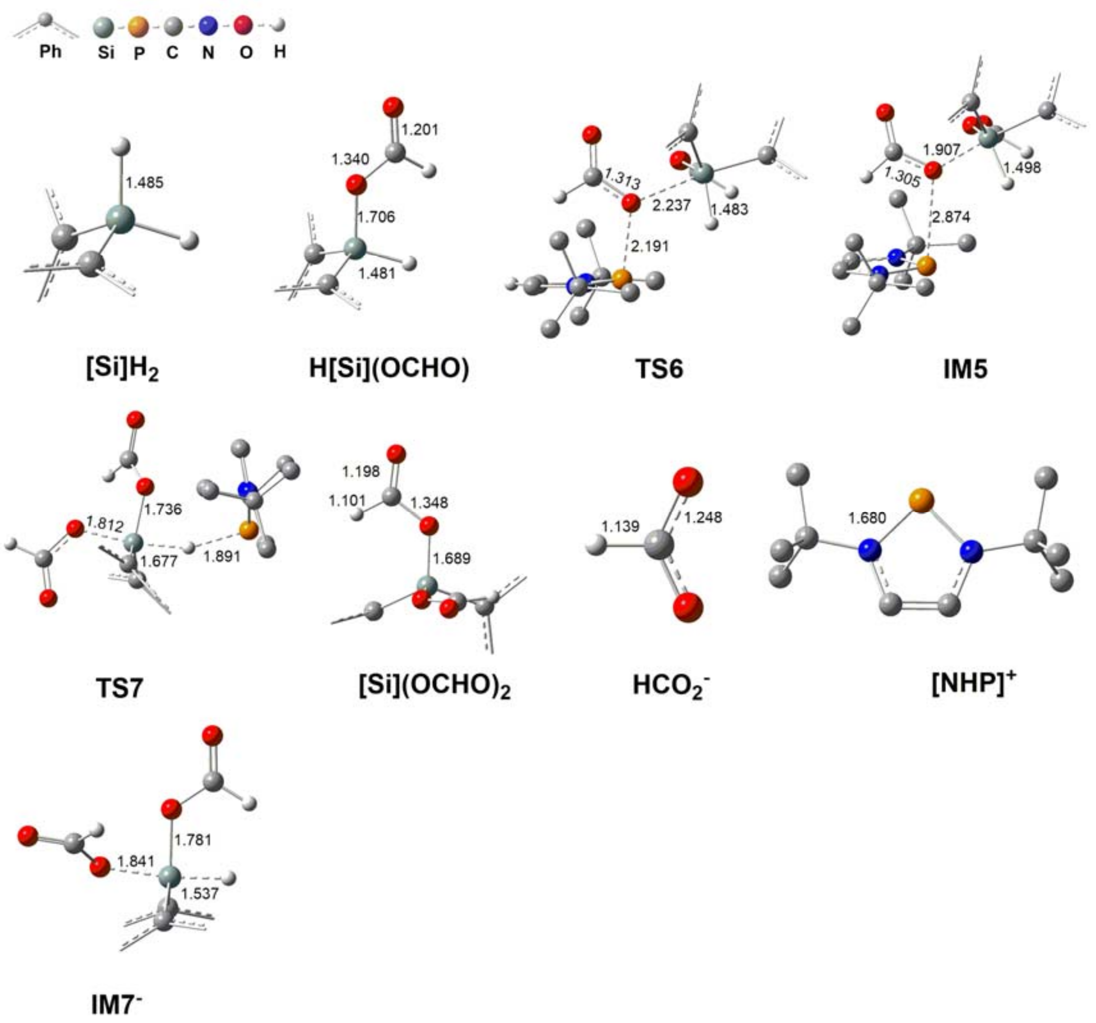

Figure S4. M06-2X/6-31G(d,p) optimized structures of stationary points not shown in Figure 2 and Figure 3, along with key bond lengths in angstroms. Trivial H atoms are omitted for clarity.

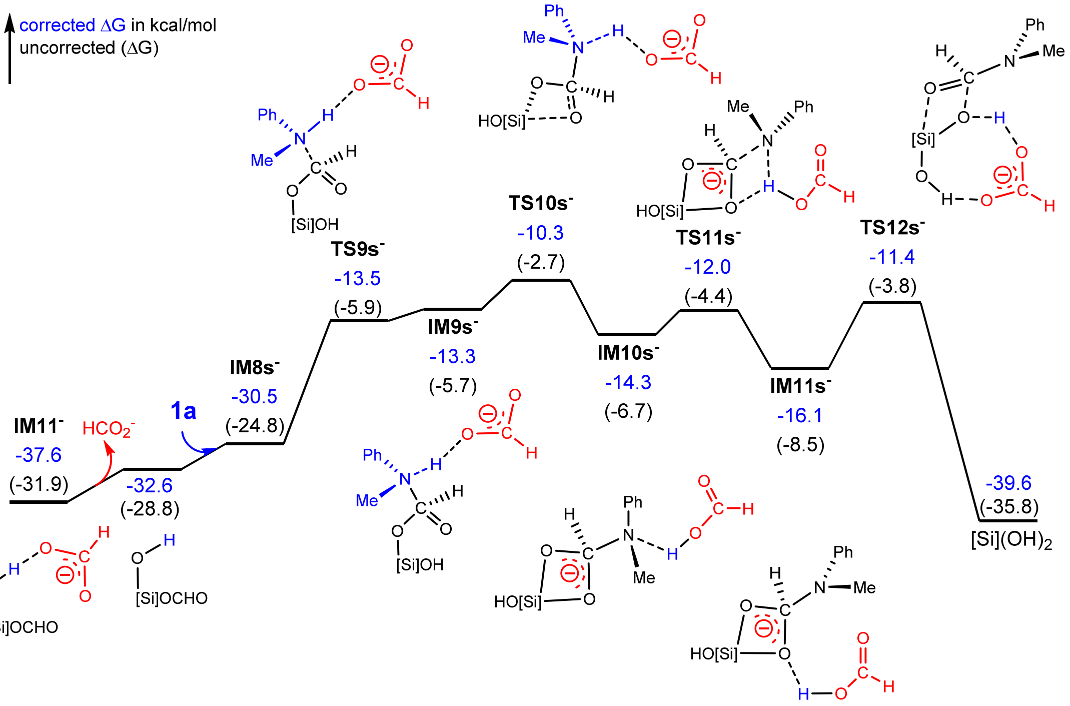

Figure S5. Free energy profile for the conversion of HO[Si]OCHO+ **1a** → **1b** + [Si](OH)$_2$. Energies are relative to **[NHP]**H, 1a, [Si]H$_2$, and CO_2, and are mass balanced.

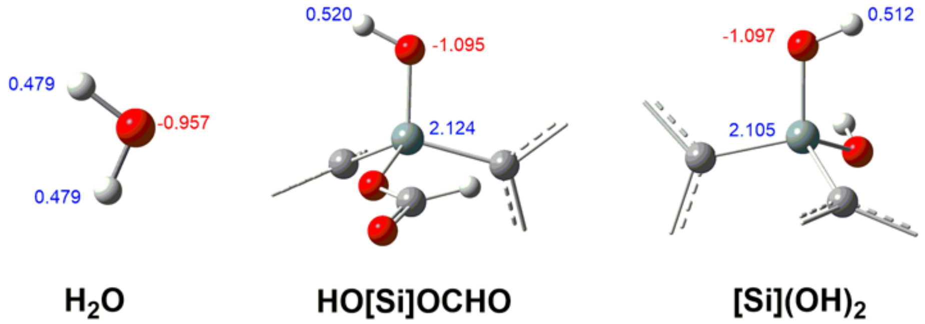

Figure S6. NBO charges (in *e*) of H_2O, HO[Si]OCHO and [Si](OH)$_2$. As indicated by the NBO charges, the hydroxyl groups in HO[Si]OCHO and [Si](OH)$_2$ are more polarized than that in water.

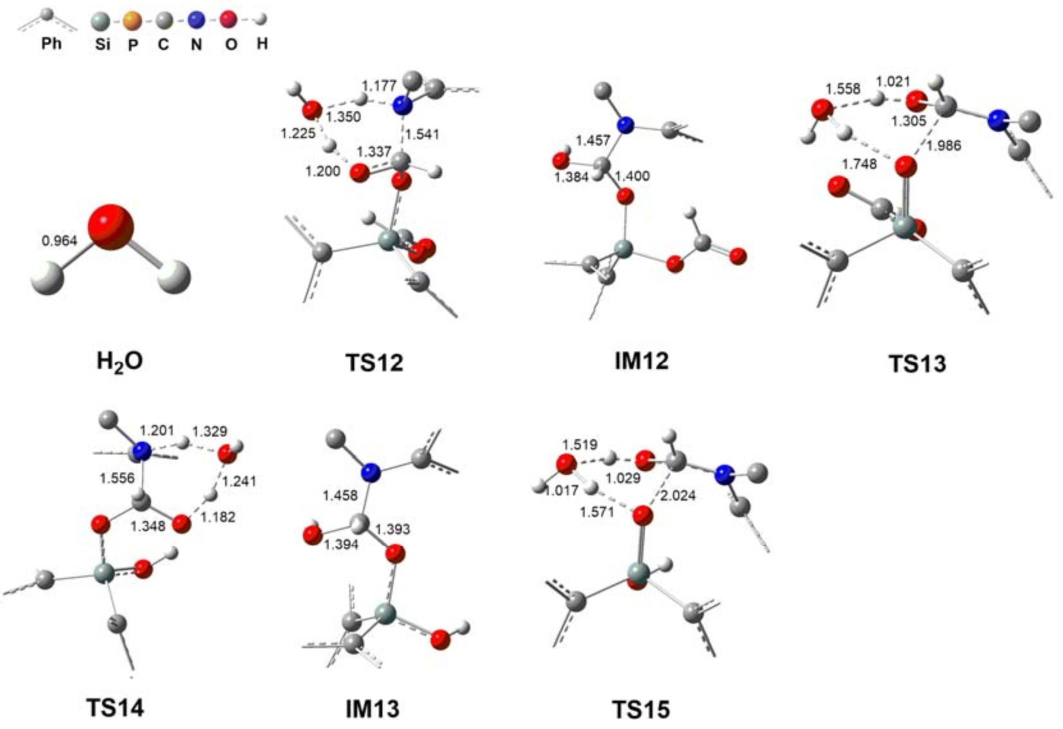

Figure S7. M06-2X/6-31G(d,p) optimized structures of stationary points shown in Figure 4, along with key bond lengths in angstroms. Trivial H atoms are omitted for clarity.

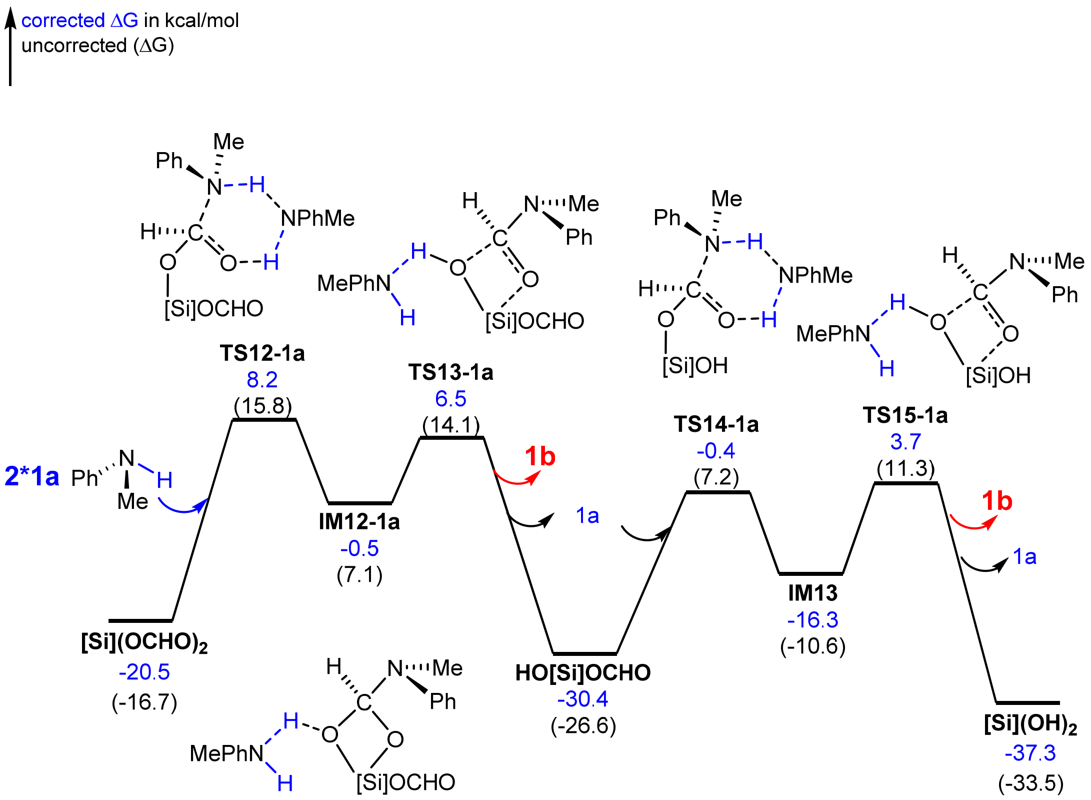

Figure S8. 1a-aided aminolysis pathway for eq 4 ([Si]$(OCHO)_2$ + 2***1a** → 2***1b** + [Si]$(OH)_2$) through mode A. Free energies are relative to **[NHP]**H, 1a, [Si]H_2, and CO_2, and are mass balanced.

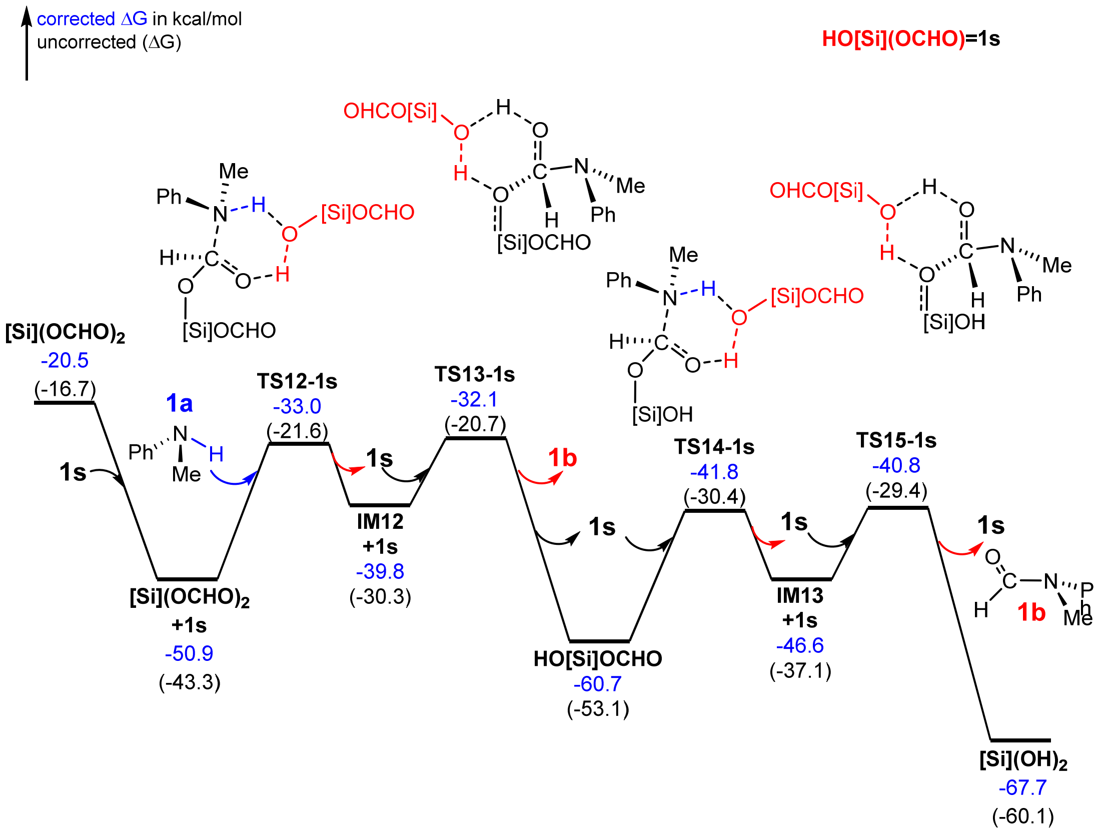

Figure S9. HO[Si]OCHO -aided aminolysis pathway for eq 4 ([Si]$(OCHO)_2$ + 2***1a** → 2***1b** + [Si]$(OH)_2$) through mode A. Free energies are relative to **[NHP]**H, 1a, [Si]H_2, and CO_2, and are mass balanced.

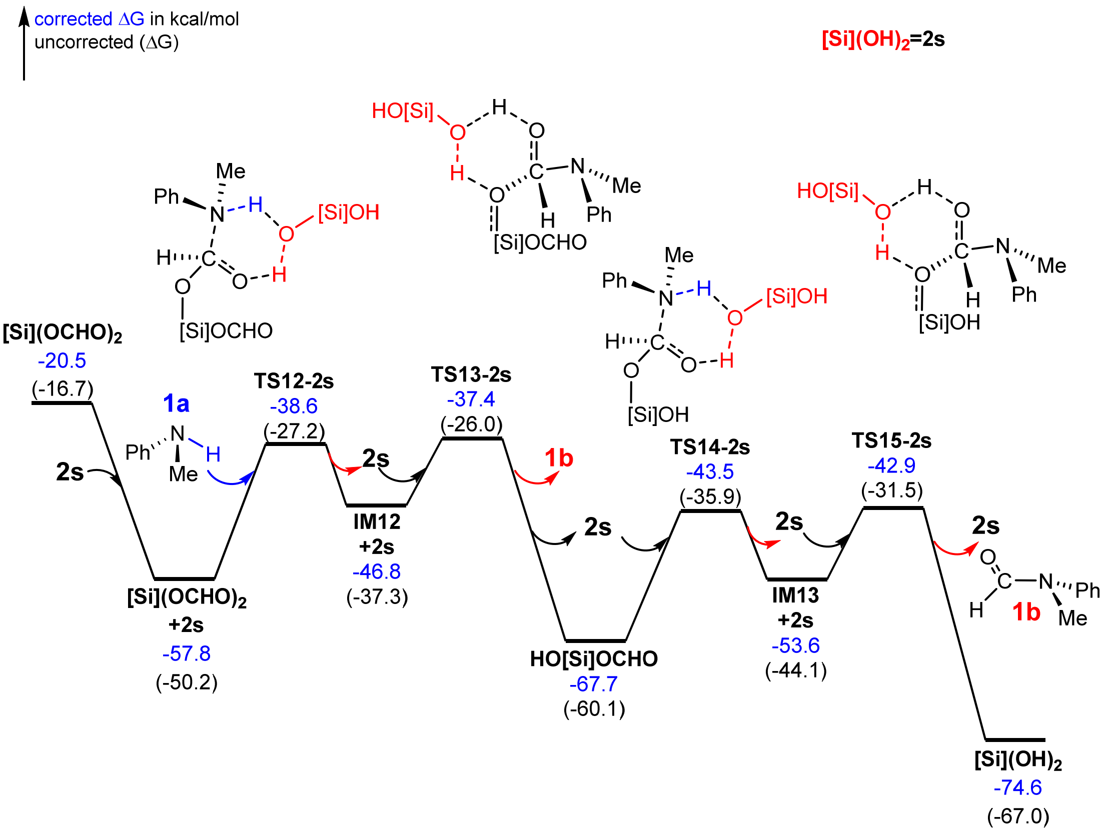

Figure S10. [Si](OH)$_2$-aided aminolysis pathway for eq 4 ([Si](OCHO)$_2$ + 2***1a** → 2***1b** + [Si](OH)$_2$) through mode A. Free energies are relative to **[NHP]**H, 1a, [Si]H$_2$, and CO_2, and are mass balanced.

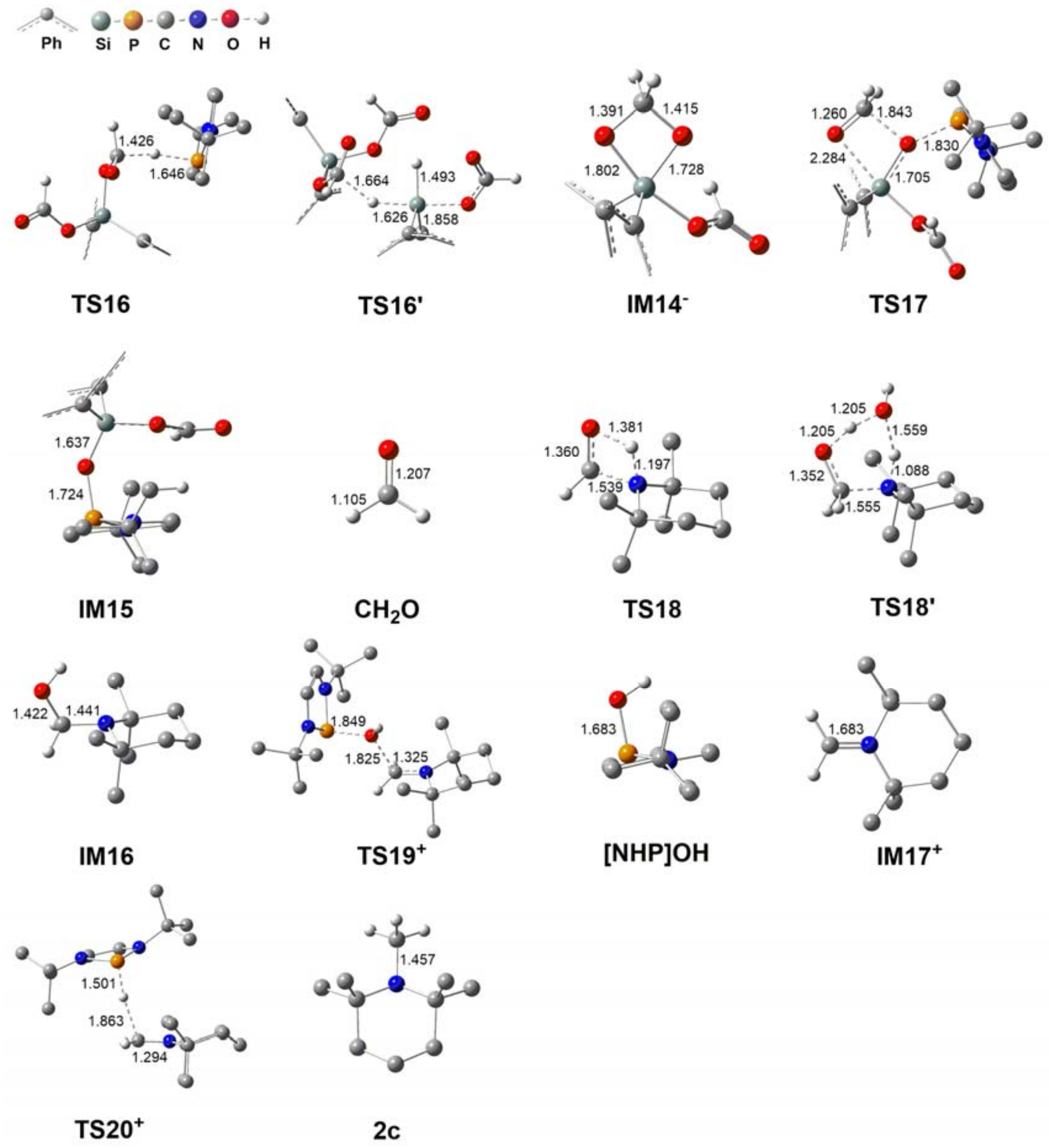

Figure S11. M06-2X/6-31G(d,p) optimized structures of stationary points not shown in Figure 5, along with key bond lengths in angstroms. Trivial H atoms are omitted for clarity.

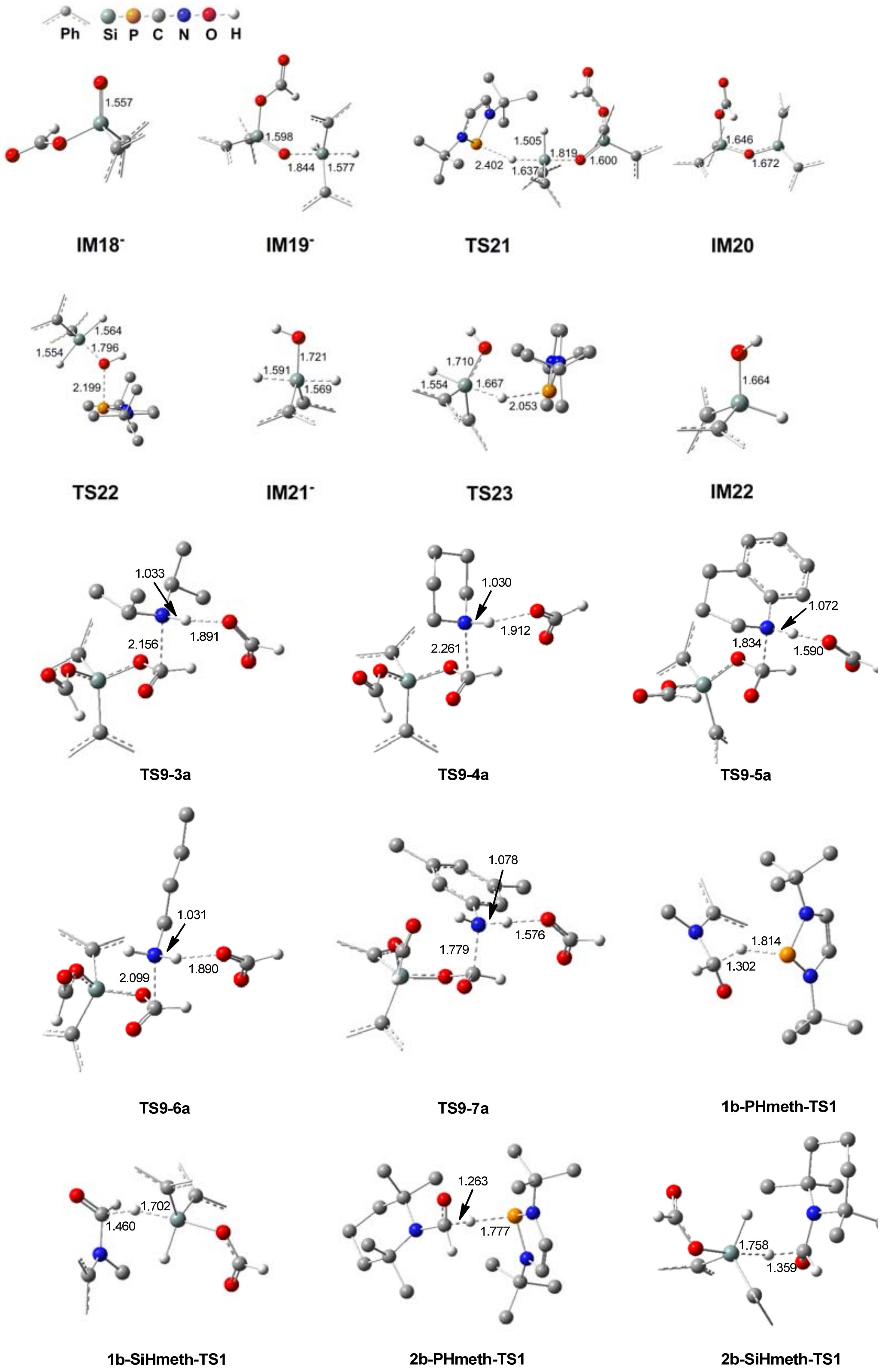

Figure S12. M06-2X/6-31G(d,p) optimized structures of stationary points shown in Figure S13 of SI 2 and Table 2, along with key bond lengths in angstroms. Trivial H atoms are omitted for clarity.

Table S1: Comparisons of the barriers for the conversion of the second formyloxy group of $[Si](OCHO)_2$.

Substrate		Formylation	Methylation	
			Hydride source	
			**[NHP]**H	HCO_2^--[Si]H_2
		ΔG≠	ΔG≠	ΔG≠
**1a**	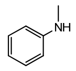	27.3 (29.2)	29.0 (29.0)	28.7 (30.6)
**2a**	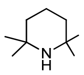	31.0 (32.9)		
**3a**	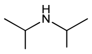	24.7 (26.6)		

SI 2: Regeneration of **[NHP]**H catalyst from **[NHP]**O[Si]OCHO and **[NHP]**OH

According to our computed methylation pathway in Figure 5 in main text, methylation consumes catalyst, resulting in **[NHP]**O[Si]OCHO (i.e. **IM15**) and **[NHP]**OH species, which seemingly contradicts to the fact that the reactions could run catalytically. However, the two species can be recovered to **[NHP]**H feasibly. Figure S13(A) describes the mechanism to recover **[NHP]**O[Si]OCHO. First, the species dissociates to $\mathbf{[NHP]}^+$ and OCHO[Si]=O^-($\mathbf{IM18^-}$) at an energy cost of 16.7 kcal/mol. Subsequently, the OCHO[Si]=O^- anion associates with [Si]H_2 to activate a Si–H bond of [Si]H_2, reaching $\mathbf{IM19^-}$. Finally, $\mathbf{[NHP]}^+$ grabs the activated $H^{\delta-}$ from $\mathbf{IM19^-}$ via **TS21**, regenerating **[NHP]**H and giving **IM20**. Overall, the recovery crosses an overall barrier of 19.1kcal/mol and is exergonic by 5.3 kcal/mol. Thus **[NHP]**H can be regenerated from **[NHP]**O[Si]OCHO easily.

IM20 featuring a Si–O–Si linkage, we reasoned that a side reaction via the pathway connecting the path from [Si]$(OCHO)_2$ + **IM3** to **IM15** in Figure 5 with Figure S13(A) could be the route to produce the experimentally-observed siloxane by-product via the pathway sketched in Figure S14. Because amine is not involved in this pathway, the side reaction could take place in the reaction of [Si]H_2 with CO_2 in the presence of **[NHP]**H (without amines). Consistently, siloxane by-product was observed under this condition.

Figure S13(B) displays the mechanism to convert **[NHP]**OH to **[NHP]**H. First, [Si]H_2 grabs the hydroxyl group from **[NHP]**OH via **TS22**, resulting in an ion pair of [Si]$H_2(OH)^-$ ($\mathbf{IM21^-}$)/$\mathbf{[NHP]}^+$. Subsequently, $\mathbf{[NHP]}^+$ takes a $H^{\delta-}$ from the anionic $\mathbf{IM21^-}$, giving HO[Si]H and **[NHP]**H. The former species can be further reacted to give [Si]$(OH)_2$. The catalyst recovery is also energetically feasible with an overall barrier of 23.5 kcal/mol and is exergonic by 8.3 kcal/mol.

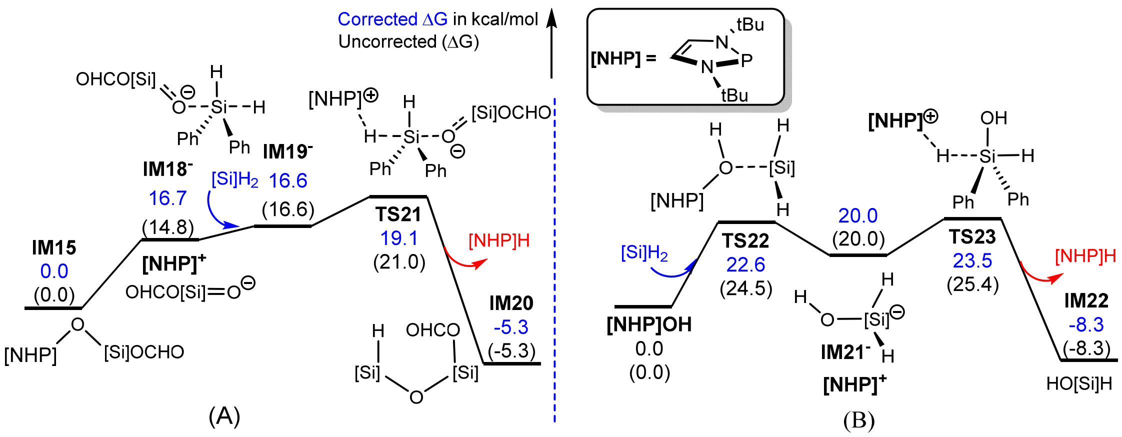

Figure S13: Free energy profiles for the recoveries of **[NHP]**O[Si]OCHO (**A**) and **[NHP]**OH (B) to **[NHP]**H. Optimized structures of all stationary points are displayed in Figure S12. Values in parentheses are uncorrected relative free energies.

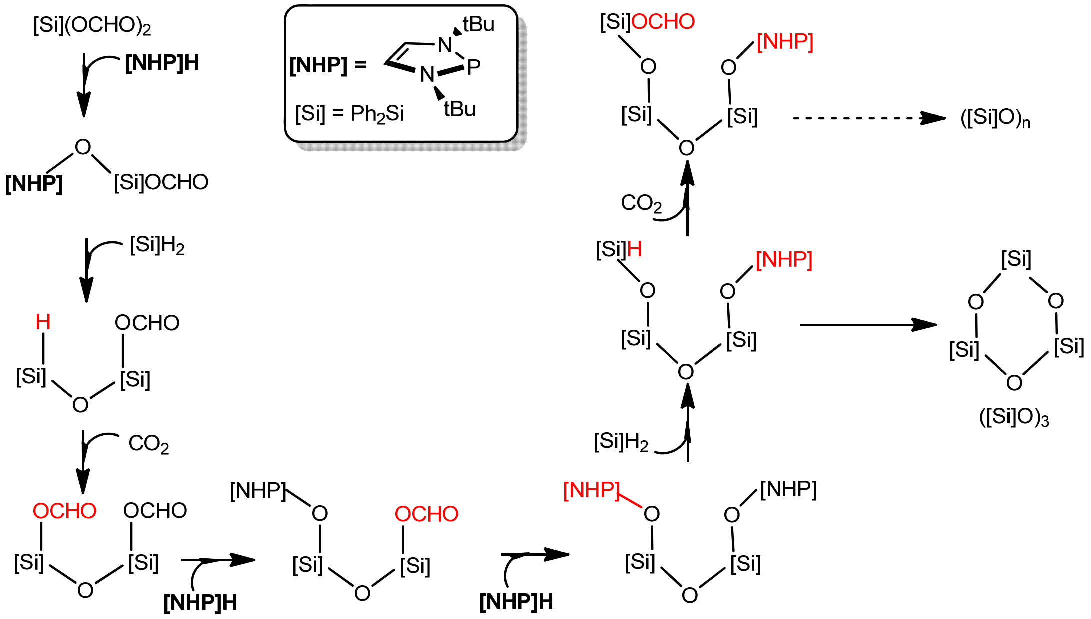

Figure S14. Schematic description to form byproduct siloxane. The sites in red represent the sites at which the next step take place.

SI 3: Experimental details and results

General information

Unless otherwise indicated, all reactions were carried out under N_2 with glovebox techniques; CD_3CN and $CDCl_3$ were purchased from Acros Organics. Amine substrates were commercially available and dried using CaH_2. All ^1H NMR (300 MHz, 400 MHz and 500 MHz), and ^{13}C NMR (125 MHz) spectra were recorded on a spectrometer in CD_3CN, DMSO or $CDCl_3$ reported in parts per million (ppm, δ). ^1H NMR Spectroscopy splitting patterns were designated as singlet (s), doublet (d), triplet (t), quartet (q). Splitting patterns that could not be interpreted or easily visualized were designated as multiplet (m) or broad (br). Infrared spectra were recorded on a JASCO FT/IR-480 spectrophotometer and reported as wave number (cm^{-1}). The N-formylation/methylation reaction was carried out in a SKY-100C shaker incubator (Shanghai Sukun Industry & Commerce Co., Ltd.). HRMS was recorded on a commercial apparatus (ESI). The catalyst 1,3,2-diazaphospholene (**[NHP]**H) was prepared according to literatures.[1]

Procedure for N-formylation of amine using CO_2 and Ph_2SiH_2.

1,3,2-Diazaphospholene(**[NHP]**H, 5 mg, 0.025 mmol), Ph_2SiH_2 (278 μL, 1.5 mmol), amine (0.5 mmol), 1,3,5-trimethoxybenzene (16.8 mg, 0.1 mmol, internal standard for NMR yield) and CD_3CN (0.40 mL) were loaded in a dried J-Young Tube in glovebox with N_2 atmosphere. After two cycles of freeze-pump-thaw, the J-Young Tube was then filled with CO_2 at –196 °C. Approximately 45 mg of CO_2 was introduced into the reaction mixture by measuring the mass of the J-Young Tube before and after the introduction of CO_2.The reaction mixture was shaken in a shaker incubator at room temperature for 5-6 hours and monitored by NMR spectroscopy. The reaction mixture was concentrated under reduced pressure, and the residue was purified by column chromatography on silica gel (petroleum ether/EtOAc) to furnish the corresponding product.

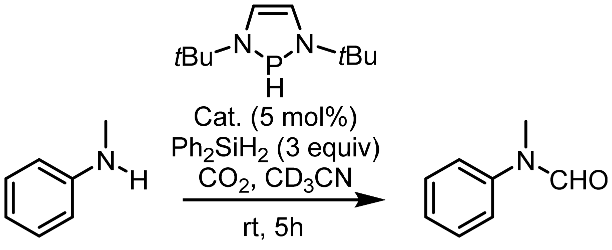

1a **1b**

NMR yield 96%

***N*-Methyl-*N*-phenylformamide (GZH-538)**

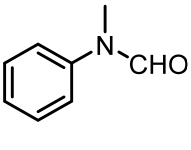

1b (96%). Purified by column chromatography on silica gel (petroleum ether/EtOAc = 20:1-5:1)**.** ^{1}H NMR (500 MHz, $CDCl_3$):δ = 8.48 (s, 1H), 7.42 (t, 2H, J = 7.5 Hz), 7.28 (t, 1H, J = 7.5 Hz), 7.18 (2H, d, J = 8.0 Hz), 3.33 (s, 3H); ^{13}C NMR (125 MHz, $CDCl_3$): δ = 162.4, 142.2, 129.6, 126.4, 122.4, 32.1. IR (KBr) 2920, 1678, 1597, 1497, 1350, 1116; HRMS *m/z* calcd for C8H10NO:[(*M*+*H*)]$^+$ 136.0757; found:136.0756.

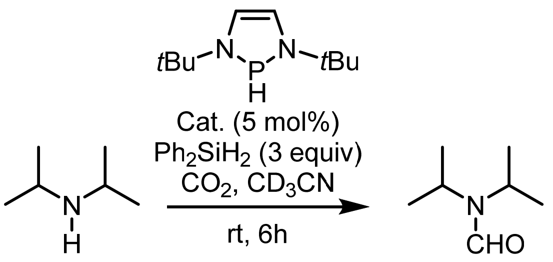

3a **3b**

NMR yield 56%

***N*,*N*-Diisopropylformamide (GZH-536)**

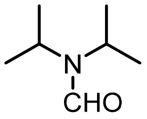

3b (56%). Purified by column chromatography on silica gel (petroleum ether/EtOAc = 12:1-5:1)**.** ^{1}H NMR (500 MHz, CD_3CN):δ = 8.14 (s, 1H), 4.03 (sept, 1H J = 6.5 Hz), 3.67 (sept, 1H, J = 7.0 Hz), 1.26-1.24 (m, 12H);^{13}C NMR (125 MHz, CD_3CN): δ = 162.3, 47.7, 44.4, 23.1, 20.4; IR (KBr) 2973, 1668, 1437, 1305, 1207; HRMS (ESI): *m/z* calcd for C7H16NO: 130.1226 [(*M*+*H*)]$^+$; found: 130.1226.

Procedure for N-methylation of amine using CO_2 and Ph_2SiH_2:

1,3,2-Diazaphospholene(**[NHP]**H, 20 mg, 0.1 mmol), Ph_2SiH_2 (278 µL, 1.5 mmol), 2,2,6,6-teramethylpiperidineamine (70.5 mg, 0.5 mmol), 1,3,5-trimethoxybenzene (16.8 mg, 0.1 mmol, internal standard) and CD_3CN (0.40 mL) were loaded in a dried J-Young Tube in glovebox under N_2 atmosphere. After two cycles of freeze-pump-thaw, the J-Young Tube was then filled with CO_2 at –196 ^{o}C. Approximately 45 mg of CO_2 was introduced into the reaction mixture by measuring the mass of the J-Young Tube before and after the introduction of CO_2.The reaction mixture was shaken in a shaker incubator at 50 ^{o}C for 12 hours and monitored by NMR spectroscopy. The reaction mixture was concentrated under reduced pressure, and the residue was purified by column chromatography on silica gel (petroleum ether/EtOAc) to furnish the corresponding product.

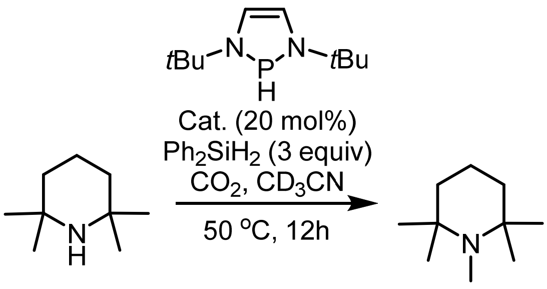

2a **2c**

NMR yield 65%

1,2,2,6,6-Pentamethylpiperidine (GZH-541)

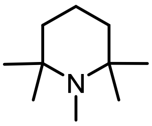

2c (65%). Purified by column chromatography on silica gel (petroleum ether/EtOAc = 15:1-3:1)**.** ^{1}H NMR (500 MHz, $CDCl_3$):δ = 2.24 (s, 3H), 1.56-1.50 (m, 2H), 1.46-1.44 (m, 4H), 1.05 (s, 12H); ^{13}C NMR (125 MHz, $CDCl_3$): δ = 54.1, 41.6, 28.9, 26.7, 18.3; IR (KBr) 2966, 2929, 1451, 1375, 1360, 1267, 1122; HRMS *m/z* calcd for C10H22N[$(M+H)]^+$: 156.1747; found: 156.1746.

References

(1) (a) Gudat, D.; Haghverdi, A.; Nieger, M., *Angew. Chem. Int. Ed.* **2000**, *39*, 3084. (b) Burck, S.; Gudat, D.; Nieger, M.; Du Mont, W.-W., *J. Am. Chem. Soc.* **2006**, *128*, 3946.

NMR spectra

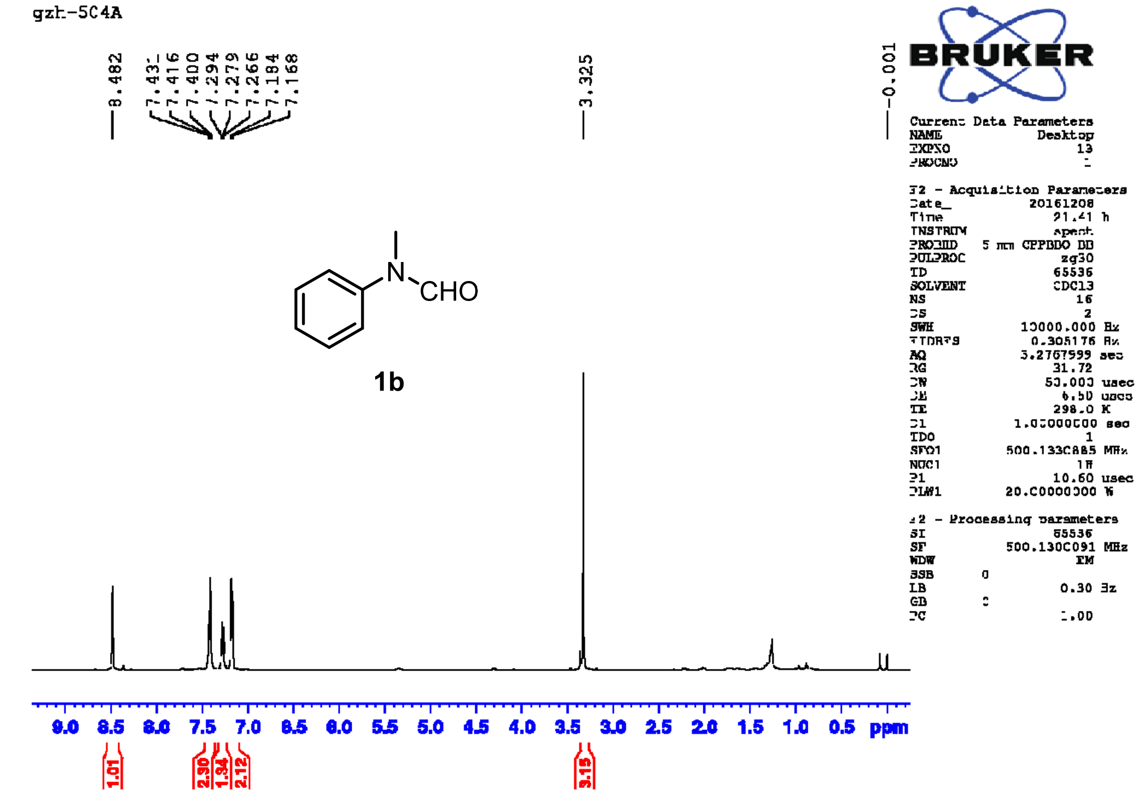
gzh-5C4A
8.482
7.416
7.400
7.294
7.279
7.266
7.184
7.168
3.325
-0.001
N
CHO
1b
Current Data Parameters
F2 - Acquisition Parameters
F2 - Processing parameters
9.0 8.5 8.0 7.5 7.0 6.5 6.0 5.5 5.0 4.5 4.0 3.5 3.0 2.5 2.0 1.5 1.0 0.5 ppm
1.01
2.30
1.34
2.12
3.15

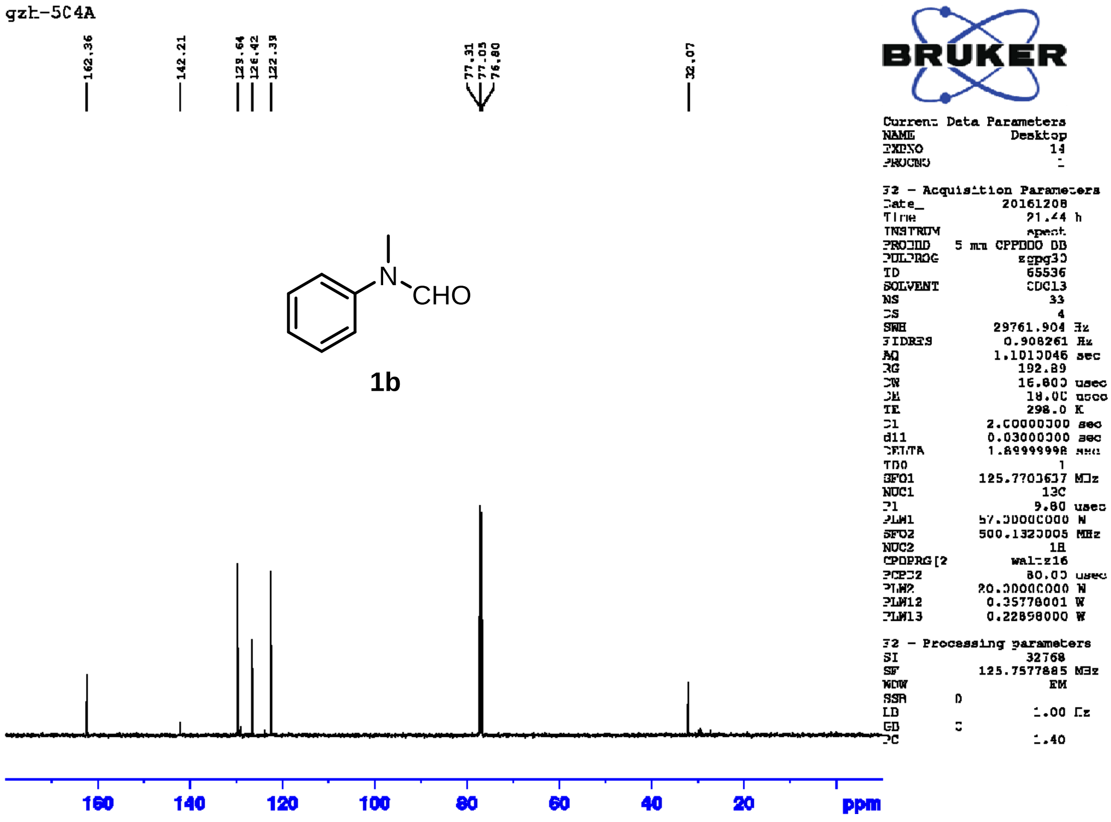
gzh-5C4A
162.36
142.21
129.64
126.42
122.39
77.31
77.05
76.80
32.07
N
CHO
1b
Current Data Parameters
F2 - Acquisition Parameters
F2 - Processing parameters
160 140 120 100 80 60 40 20 ppm

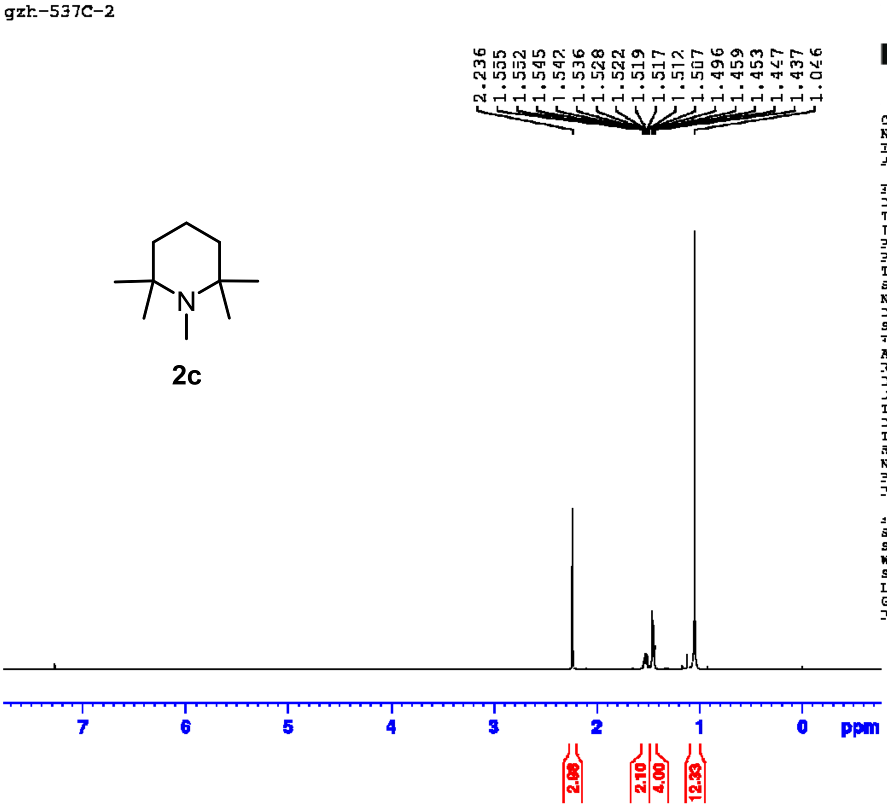

Current Data Parameters
NAME Desktop
EXPNO 23
PROCNO 1

F2 - Acquisition Parameters
Date_ 20161216
Time 22.16 h
INSTRUM spect
PROBHD 5 mm CPPBBO BB
PULPROG zg30
TD 65536
SOLVENT CDCl3
NS 16
DS 2
SWH 10000.000 Hz
FIDRES 0.305176 Hz
AQ 3.2767999 sec
RG 25.06
DW 50.000 usec
DE 6.50 usec
TE 298.1 K
D1 1.00000000 sec
TD0 1
SFO1 500.1330885 MHz
NUC1 1H
P1 10.60 usec
PLW1 20.00000000 W

F2 - Processing parameters
SI 65536
SF 500.1300071 MHz
WDW EM
SSB 0
LB 0.30 Hz
GB 0
PC 1.00

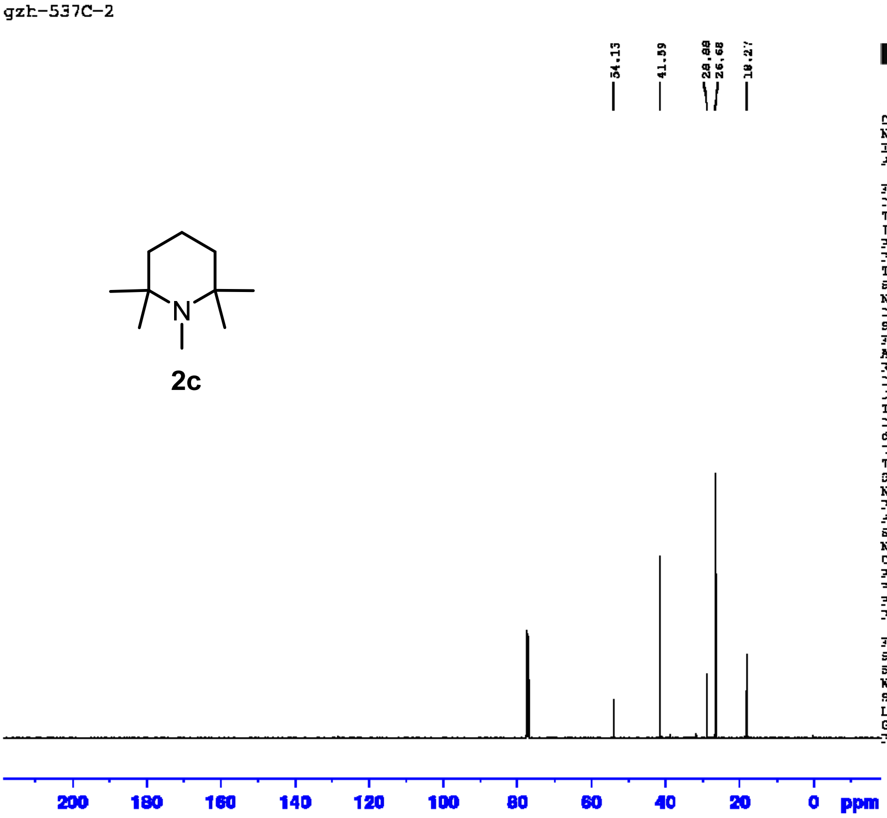

Current Data Parameters
NAME Desktop
EXPNO 24
PROCNO 1

F2 - Acquisition Parameters
Date_ 20161216
Time 22.11 h
INSTRUM spect
PROBHD 5 mm CPPBBO BB
PULPROG zgpg30
TD 65536
SOLVENT CDCl3
NS 35
DS 4
SWH 29761.904 Hz
FIDRES 0.908261 Hz
AQ 1.1010048 sec
RG 192.89
DW 16.800 usec
DE 18.00 usec
TE 298.0 K
D1 2.00000000 sec
d11 0.03000000 sec
DELTA 1.89999998 sec
TD0 1
SFO1 125.7703637 MHz
NUC1 13C
P1 9.80 usec
PLW1 57.00000000 W
SFO2 500.1320005 MHz
NUC2 1H
CPDPRG[2 waltz16
PCPD2 80.00 usec
PLW2 20.00000000 W
PLW12 0.35770001 W
PLW13 0.22898000 W

F2 - Processing parameters
SI 32768
SF 125.7577439 MHz
WDW EM
SSB 0
LB 1.00 Hz
GB 0
PC 1.40

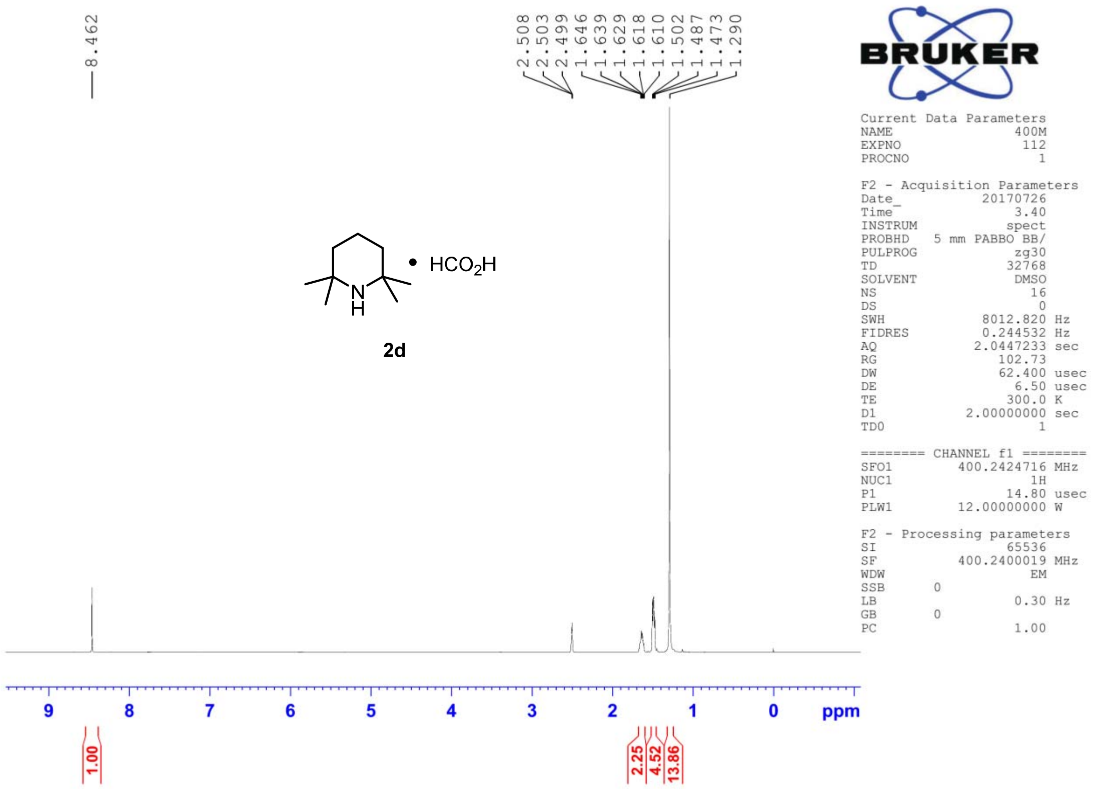
8.462
2.508 2.503 2.499 1.646 1.639 1.629 1.618 1.610 1.502 1.487 1.473 1.290
HCO2H
N H
2d
BRUKER
Current Data Parameters
NAME 400M
EXPNO 112
PROCNO 1
F2 - Acquisition Parameters
Date_ 20170726
Time 3.40
INSTRUM spect
PROBHD 5 mm PABBO BB/
PULPROG zg30
TD 32768
SOLVENT DMSO
NS 16
DS 0
SWH 8012.820 Hz
FIDRES 0.244532 Hz
AQ 2.0447233 sec
RG 102.73
DW 62.400 usec
DE 6.50 usec
TE 300.0 K
D1 2.00000000 sec
TD0 1
======== CHANNEL f1 ========
SFO1 400.2424716 MHz
NUC1 1H
P1 14.80 usec
PLW1 12.00000000 W
F2 - Processing parameters
SI 65536
SF 400.2400019 MHz
WDW EM
SSB 0
LB 0.30 Hz
GB 0
PC 1.00
9 8 7 6 5 4 3 2 1 0 ppm
1.00 2.25 4.52 13.86

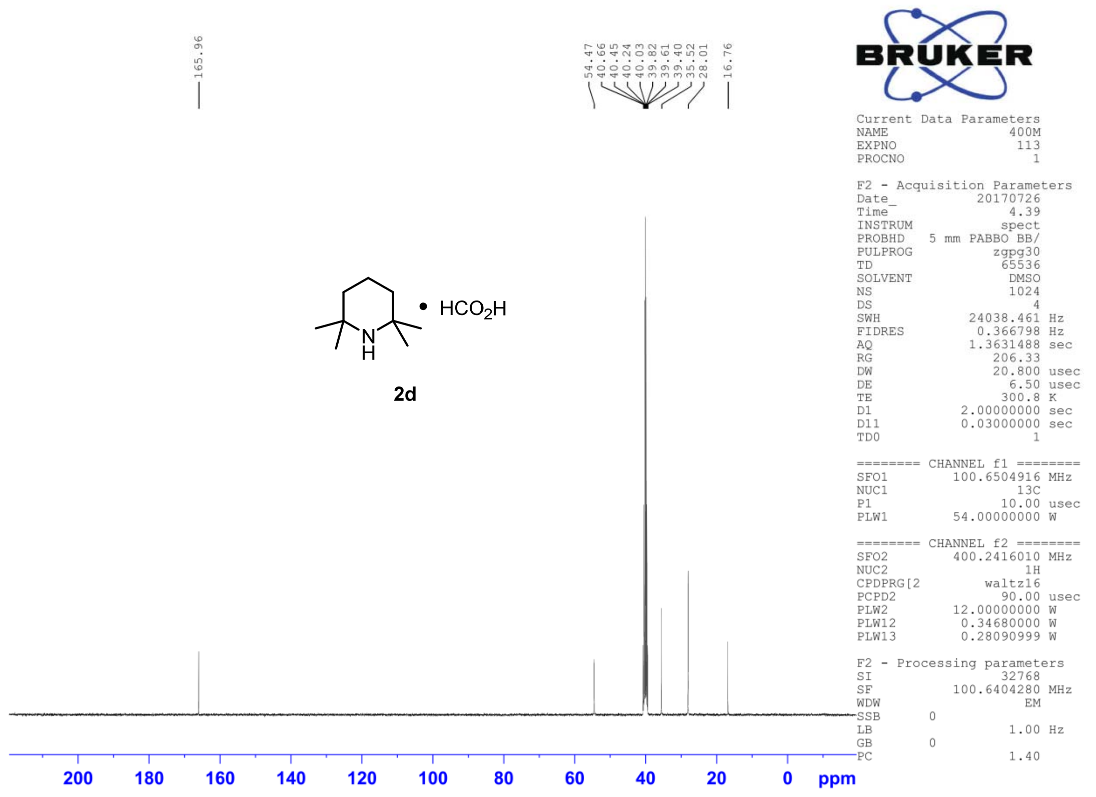
165.96
54.47 40.66 40.45 40.24 40.03 39.82 39.61 39.40 35.52 28.01 16.76
HCO2H
N H
2d
BRUKER
Current Data Parameters
NAME 400M
EXPNO 113
PROCNO 1
F2 - Acquisition Parameters
Date_ 20170726
Time 4.39
INSTRUM spect
PROBHD 5 mm PABBO BB/
PULPROG zgpg30
TD 65536
SOLVENT DMSO
NS 1024
DS 4
SWH 24038.461 Hz
FIDRES 0.366798 Hz
AQ 1.3631488 sec
RG 206.33
DW 20.800 usec
DE 6.50 usec
TE 300.8 K
D1 2.00000000 sec
D11 0.03000000 sec
TD0 1
======== CHANNEL f1 ========
SFO1 100.6504916 MHz
NUC1 13C
P1 10.00 usec
PLW1 54.00000000 W
======== CHANNEL f2 ========
SFO2 400.2416010 MHz
NUC2 1H
CPDPRG[2 waltz16
PCPD2 90.00 usec
PLW2 12.00000000 W
PLW12 0.34680000 W
PLW13 0.28090999 W
F2 - Processing parameters
SI 32768
SF 100.6404280 MHz
WDW EM
SSB 0
LB 1.00 Hz
GB 0
PC 1.40
200 180 160 140 120 100 80 60 40 20 0 ppm

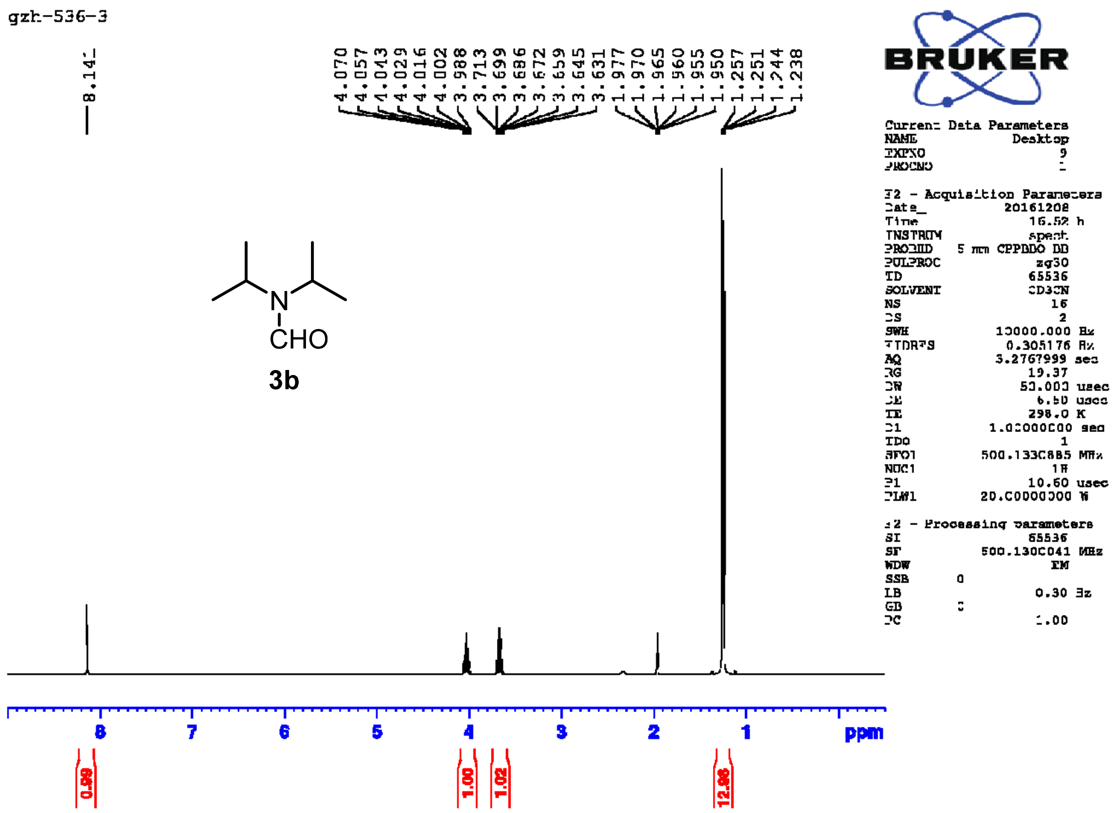
gzh-536-3
8.141
4.070 4.057 4.043 4.029 4.016 4.002 3.988 3.713 3.699 3.686 3.672 3.659 3.645 3.631 1.977 1.970 1.965 1.960 1.955 1.950 1.257 1.251 1.244 1.238
N
CHO
3b
BRUKER
Current Data Parameters
NAME Desktop
EXPNO 9
PROCNO 1
F2 - Acquisition Parameters
Date_ 20161208
Time 16.52 h
INSTRUM spect
PROBHD 5 mm CPPBBO BB
PULPROG zg30
TD 65536
SOLVENT CD3CN
NS 16
DS 2
SWH 10000.000 Hz
FIDRES 0.305176 Hz
AQ 3.2767999 sec
RG 19.37
DW 50.000 usec
DE 6.50 usec
TE 298.0 K
D1 1.00000000 sec
TD0 1
SFO1 500.1330885 MHz
NUC1 1H
P1 10.60 usec
PLW1 20.00000000 W
F2 - Processing parameters
SI 65536
SF 500.1300041 MHz
WDW EM
SSB 0
LB 0.30 Hz
GB 0
PC 1.00
8 7 6 5 4 3 2 1 ppm
0.99 1.00 1.02 12.98

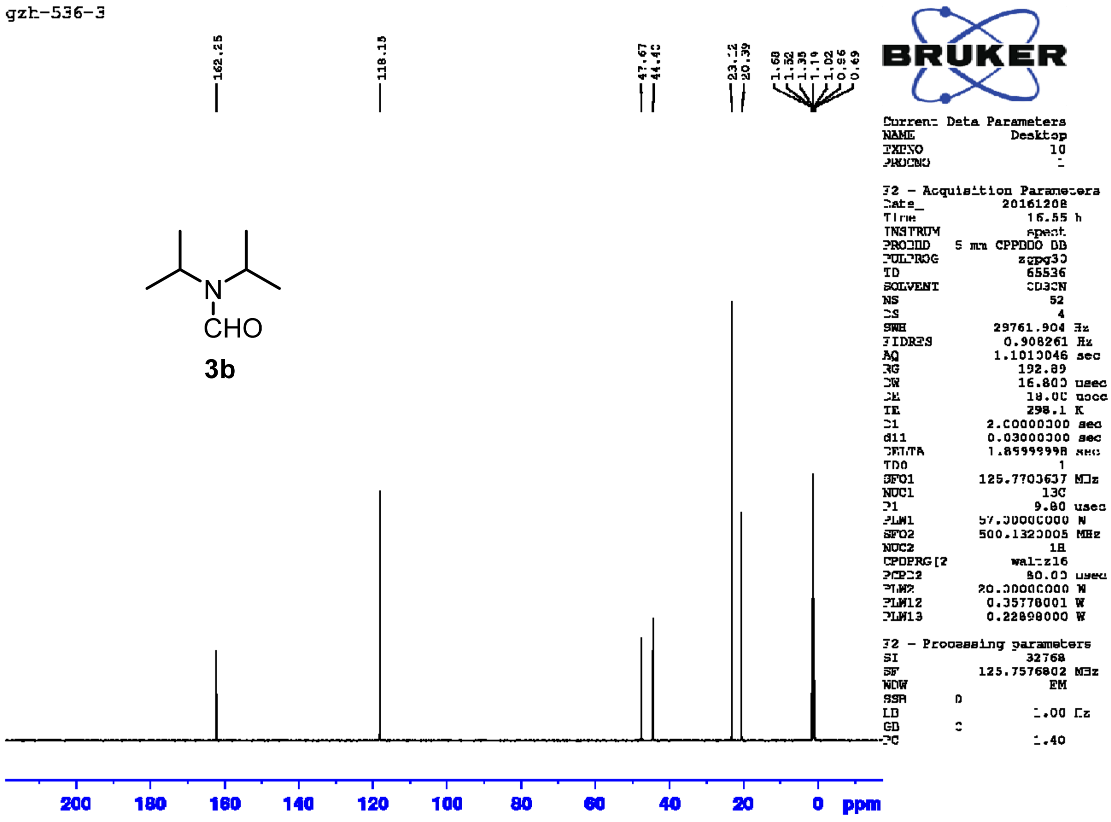
gzh-536-3
162.25
118.15
47.67 44.40
23.12 20.39
1.68 1.52 1.35 1.19 1.02 0.86 0.69
N
CHO
3b
BRUKER
Current Data Parameters
NAME Desktop
EXPNO 10
PROCNO 1
F2 - Acquisition Parameters
Date_ 20161208
Time 16.55 h
INSTRUM spect
PROBHD 5 mm CPPBBO BB
PULPROG zgpg30
TD 65536
SOLVENT CD3CN
NS 52
DS 4
SWH 29761.904 Hz
FIDRES 0.908261 Hz
AQ 1.1010048 sec
RG 192.89
DW 16.800 usec
DE 18.00 usec
TE 298.1 K
D1 2.00000000 sec
d11 0.03000000 sec
DELTA 1.89999998 sec
TD0 1
SFO1 125.7703637 MHz
NUC1 13C
P1 9.80 usec
PLW1 57.00000000 W
SFO2 500.1320005 MHz
NUC2 1H
CPDPRG[2 waltz16
PCPD2 80.00 usec
PLW2 20.00000000 W
PLW12 0.35770001 W
PLW13 0.22898000 W
F2 - Processing parameters
SI 32768
SF 125.7576802 MHz
WDW EM
SSB 0
LB 1.00 Hz
GB 0
PC 1.40
200 180 160 140 120 100 80 60 40 20 0 ppm

^{1}H NMR spectrum of the crude material leading to **3b** (in CD_3CN).

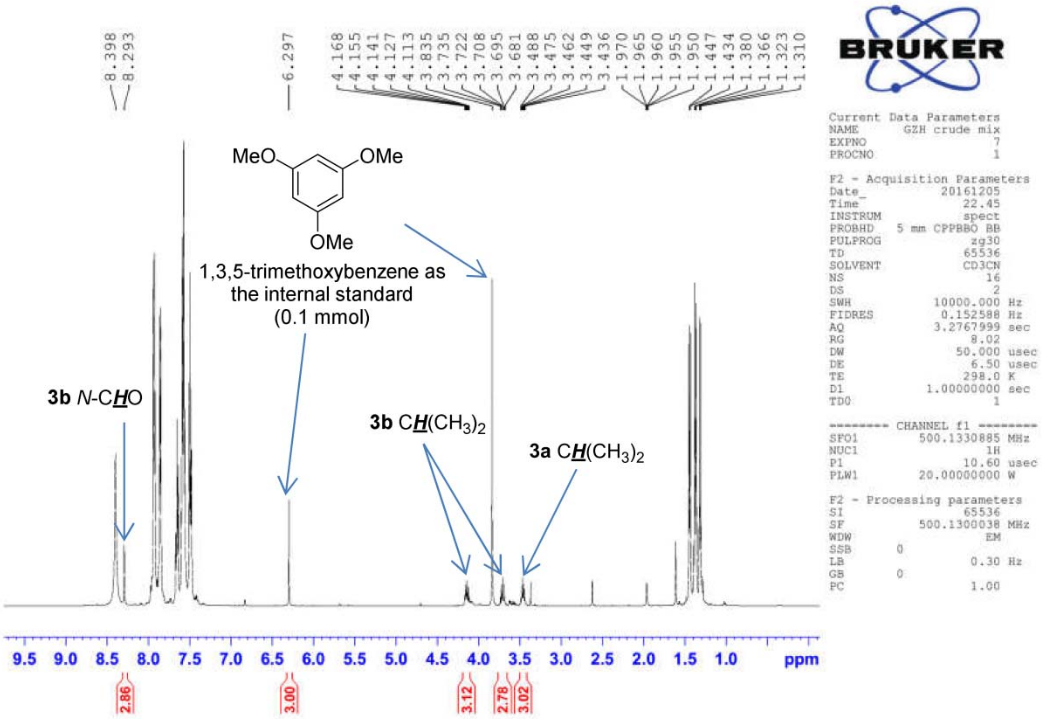

SI 4: Cartesian Coordinates in Å, SCF Energies and Free Energies (in a.u.) at 298.15 K and 1 atm for the Optimized Structures [BS1= 6-31G(d,p), BS2=6-311++G(d,p)]

[NHP]H

M06-2X/BS1 SCF energy in solution :
-844.300057 a.u.
M06-2X/BS2 SCF energy in solution:
-844.457126 a.u.
M06-2X/BS2 Free energy in solution:
-844.197712 a.u.

C	-0.670926	1.490938	-0.671944
C	0.671021	1.490908	-0.672028
H	-1.288065	2.381028	-0.693261
H	1.288195	2.380971	-0.693422
N	-1.282752	0.210162	-0.693478
N	1.282789	0.210083	-0.693554
P	-0.000022	-0.933592	-0.997021
H	-0.000058	-0.751499	-2.411081
C	2.375391	-0.077249	0.276298
C	-2.375403	-0.077241	0.276285
C	3.044233	-1.386966	-0.141473
H	3.432694	-1.311604	-1.161943
H	3.875024	-1.615018	0.532785
H	2.339525	-2.223484	-0.099038
C	3.405714	1.052410	0.198215
H	3.018140	1.988879	0.608789
H	4.289050	0.777771	0.781827
H	3.714353	1.223199	-0.837875
C	1.840164	-0.189339	1.706420
H	1.289413	0.716850	1.980899
H	1.169265	-1.049770	1.802839
H	2.661595	-0.319499	2.418526
C	-3.044834	-1.386416	-0.142232
H	-3.875481	-1.614703	0.532126
H	-3.433593	-1.310171	-1.162520
H	-2.340373	-2.223191	-0.100656
C	-3.405228	1.052918	0.199003
H	-4.288720	0.778194	0.782337
H	-3.017264	1.988889	0.610348
H	-3.713727	1.224661	-0.836972
C	-1.840106	-0.190432	1.706296
H	-1.169736	-1.051333	1.802201
H	-1.288778	0.715282	1.981185
H	-2.661555	-0.320458	2.418408

CO_2

M06-2X/BS1 SCF energy in solution :
-188.507564 a.u.
M06-2X/BS2 SCF energy in solution:
-188.575221 a.u.
M06-2X/BS2 Free energy in solution:
-188.584158 a.u.

C	0.000000	0.000000	0.000000
O	0.000000	0.000000	1.162854
O	0.000000	0.000000	-1.162854

TS1

M06-2X/BS1 SCF energy in solution :
-1032.794508 a.u.
M06-2X/BS2 SCF energy in solution:
-1033.018767 a.u.
M06-2X/BS2 Free energy in solution:
-1032.752301 a.u.

C	0.674592	-0.197233	-1.449318
C	-0.674375	-0.197328	-1.449347
H	1.321718	-0.101116	-2.310261
H	-1.321472	-0.101300	-2.310320
N	1.207360	-0.417924	-0.175987
N	-1.207165	-0.418089	-0.176034
P	0.000075	-0.276209	1.005349
H	-0.000044	1.321378	1.036961
C	-2.650221	-0.673024	0.045253

C	2.650429	-0.672745	0.045337
C	-3.466400	0.518299	-0.462207
H	-3.316716	0.674731	-1.534735
H	-4.531169	0.331184	-0.294022
H	-3.172313	1.430031	0.062320
C	-3.041073	-1.949613	-0.704326
H	-2.455287	-2.799319	-0.340764
H	-4.102636	-2.163999	-0.549333
H	-2.869615	-1.843594	-1.779762
C	-2.898905	-0.858997	1.540570
H	-2.343070	-1.714920	1.935656
H	-2.620093	0.038273	2.105050
H	-3.964286	-1.040418	1.704900
C	3.466473	0.518711	-0.462024
H	4.531205	0.332088	-0.293061
H	3.317442	0.674697	-1.534710
H	3.171642	1.430514	0.061973
C	3.041407	-1.949239	-0.704331
H	4.103107	-2.163263	-0.549775
H	2.456059	-2.799128	-0.340490
H	2.869455	-1.843316	-1.779698
C	2.899086	-0.858792	1.540651
H	2.620392	0.038491	2.105161
H	2.343115	-1.714651	1.935684
H	3.964436	-1.040400	1.704971
C	-0.000409	2.584251	0.150659
O	1.153874	2.841622	-0.012759
O	-1.154873	2.841319	-0.011908

IM1

M06-2X/BS1 SCF energy in solution :
-1032.841818 a.u.
M06-2X/BS2 SCF energy in solution:
-1033.060138 a.u.
M06-2X/BS2 Free energy in solution:
-1032.787115 a.u.

C	0.442252	-1.611970	-0.957444
C	-0.895515	-1.514884	-0.929965
H	1.038589	-2.267869	-1.574718
H	-1.604241	-2.082174	-1.516510
N	1.047135	-0.761638	-0.012614
N	-1.336920	-0.585141	0.025949
P	-0.057716	0.353793	0.616070
H	0.281298	3.461033	-1.358465
C	-2.774001	-0.254676	0.173590
C	2.519599	-0.710278	0.160263
C	-2.942594	0.760048	1.304041
H	-2.451981	1.710828	1.067987
H	-4.007484	0.961403	1.446793
H	-2.536330	0.376020	2.244999
C	-3.297593	0.342899	-1.136695
H	-3.211164	-0.376800	-1.956457
H	-4.352684	0.613672	-1.032579
H	-2.725431	1.237426	-1.398288
C	-3.540513	-1.532221	0.524212
H	-3.460040	-2.280777	-0.269345
H	-3.155733	-1.965566	1.452263
H	-4.601181	-1.301535	0.660111
C	2.847465	0.064147	1.437122
H	3.930982	0.061015	1.585002
H	2.517846	1.104945	1.365999
H	2.379850	-0.404859	2.308891
C	3.150806	-0.014686	-1.049872
H	4.241626	-0.014651	-0.960555
H	2.883177	-0.531370	-1.977103
H	2.809340	1.022869	-1.113052
C	3.054294	-2.138935	0.292565
H	2.554137	-2.662011	1.113298
H	2.916562	-2.715908	-0.625771
H	4.126709	-2.104462	0.504396
C	0.571743	2.691746	-0.622954
O	-0.147549	1.598810	-0.744704
O	1.460642	2.875215	0.185895

TS2

M06-2X/BS1 SCF energy in solution :
-1032.832195 a.u.
M06-2X/BS2 SCF energy in solution:
-1033.051006 a.u.
M06-2X/BS2 Free energy in solution:
-1032.778574 a.u.

C	0.423657	-1.688892	-0.916442
C	-0.910894	-1.550355	-0.931461
H	1.020530	-2.349569	-1.528515
H	-1.618877	-2.081200	-1.551784
N	1.022010	-0.876733	0.061731

N	-1.351770	-0.623400	0.027453
P	-0.069679	0.248719	0.698550
H	-0.297793	3.428904	0.235412
C	-2.778870	-0.231133	0.121863
C	2.497867	-0.756725	0.164443
C	-2.950306	0.774898	1.259949
H	-2.404856	1.704458	1.061457
H	-4.009838	1.025747	1.356084
H	-2.607534	0.359554	2.212783
C	-3.215833	0.408210	-1.199815
H	-2.590429	1.278056	-1.421042
H	-3.128133	-0.302555	-2.027063
H	-4.259872	0.729414	-1.136466
C	-3.614613	-1.477532	0.420667
H	-3.533826	-2.218488	-0.379841
H	-3.288327	-1.939587	1.357178
H	-4.668677	-1.200976	0.515831
C	2.850939	0.114630	1.370265
H	3.937819	0.135193	1.486199
H	2.513716	1.148291	1.233920
H	2.414012	-0.284469	2.291082
C	3.042659	-0.107011	-1.111670
H	4.131585	-0.014386	-1.051190
H	2.800684	-0.711769	-1.991423
H	2.609018	0.890041	-1.235830
C	3.092242	-2.152918	0.361380
H	2.678652	-2.623118	1.258648
H	2.893661	-2.802477	-0.495566
H	4.177150	-2.075903	0.476446
C	0.472459	2.780909	-0.219388
O	0.039354	1.572619	-0.559448
O	1.601772	3.176195	-0.401502

IM2

M06-2X/BS1 SCF energy in solution :
-1032.839207 a.u.
M06-2X/BS2 SCF energy in solution:
-1033.059526 a.u.
M06-2X/BS2 Free energy in solution:
-1032.788131 a.u.

C	0.670470	-1.590439	-1.008027
C	-0.670322	-1.590494	-1.008015
H	1.325662	-2.152425	-1.658230
H	-1.325477	-2.152521	-1.658219
N	1.195986	-0.777686	0.013402
N	-1.195886	-0.777769	0.013415
P	0.000023	0.184570	0.724577
C	-2.654111	-0.538508	0.135462
C	2.654193	-0.538345	0.135459
C	-2.920701	0.348752	1.351540
H	-2.488050	1.348315	1.227098
H	-4.000230	0.470286	1.473032
H	-2.519712	-0.099118	2.266077
C	-3.161283	0.160223	-1.130324
H	-2.997917	-0.463654	-2.014213
H	-4.233975	0.359771	-1.047305
H	-2.636558	1.109786	-1.273613
C	-3.360183	-1.882332	0.329129
H	-3.207806	-2.544685	-0.527523
H	-2.986955	-2.383294	1.227271
H	-4.436311	-1.721048	0.439947
C	2.920688	0.349069	1.351443
H	4.000204	0.470747	1.472912
H	2.487906	1.348564	1.226906
H	2.519768	-0.098761	2.266031
C	3.161344	0.160285	-1.130391
H	4.234015	0.359935	-1.047357
H	2.998066	-0.463704	-2.014216
H	2.636536	1.109784	-1.273800
C	3.360336	-1.882111	0.329288
H	2.987132	-2.382984	1.227490
H	3.207985	-2.544575	-0.527283
H	4.436457	-1.720764	0.440085
O	0.000055	1.559856	-0.456269
C	-0.000246	2.787248	0.041429
H	-0.000479	2.835076	1.146393
O	-0.000358	3.781995	-0.644959

TS3

M06-2X/BS1 SCF energy in solution :
-1032.834376 a.u.
M06-2X/BS2 SCF energy in solution:
-1033.055657 a.u.
M06-2X/BS2 Free energy in solution:
-1032.782200 a.u.

C	0.593209	-0.994135	-1.396443

C	-0.746257	-1.038527	-1.349081
H	1.229877	-1.308979	-2.210894
H	-1.413843	-1.380309	-2.127581
N	1.146013	-0.500567	-0.196592
N	-1.254498	-0.598712	-0.115353
P	-0.046442	0.139634	0.813630
C	-2.712814	-0.479123	0.119954
C	2.569250	-0.751931	0.147204
C	-2.950104	-0.001778	1.552225
H	-2.548507	1.005115	1.711657
H	-4.025811	0.034855	1.743148
H	-2.496162	-0.683739	2.278141
C	-3.308276	0.533279	-0.864237
H	-3.182938	0.195778	-1.897747
H	-4.379054	0.655774	-0.674928
H	-2.814243	1.502239	-0.750894
C	-3.357354	-1.854291	-0.068547
H	-3.210579	-2.231540	-1.084704
H	-2.931641	-2.575551	0.635543
H	-4.434408	-1.785575	0.109943
C	2.872442	-0.197100	1.538044
H	3.929917	-0.363650	1.759663
H	2.683294	0.880618	1.595492
H	2.280705	-0.699752	2.308905
C	3.468859	-0.066551	-0.885110
H	4.518246	-0.265015	-0.648169
H	3.276817	-0.441126	-1.894730
H	3.311341	1.015990	-0.881027
C	2.815254	-2.264089	0.141429
H	2.150770	-2.758025	0.857177
H	2.637119	-2.690312	-0.850190
H	3.851423	-2.476884	0.420269
O	-0.303741	1.800666	0.105051
C	0.686940	2.638223	-0.131590
H	1.696186	2.223912	0.037303
O	0.521317	3.774678	-0.515087

IM3

M06-2X/BS1 SCF energy in solution :
-1032.840037 a.u.
M06-2X/BS2 SCF energy in solution:
-1033.060799 a.u.
M06-2X/BS2 Free energy in solution:
-1032.789906 a.u.

C	0.667880	-0.949007	-1.389264
H	1.323113	-1.177235	-2.217613
C	-0.673315	-0.946719	-1.389736
H	-1.328819	-1.172878	-2.218428
C	2.655386	-0.527329	0.096976
C	3.306206	-1.858524	-0.288188
H	2.878752	-2.675578	0.300586
H	4.381644	-1.813019	-0.093772
H	3.167511	-2.084526	-1.349085
C	3.224659	0.612354	-0.754698
H	3.025665	0.445772	-1.817882
H	4.308183	0.678830	-0.617691
H	2.781054	1.569925	-0.462804
C	2.925061	-0.246025	1.574763
H	2.502909	0.715888	1.885680
H	4.005361	-0.203256	1.736510
H	2.514017	-1.035797	2.211124
C	-2.660022	-0.518644	0.096245
C	-3.315709	-1.845720	-0.294798
H	-3.179405	-2.066993	-1.356982
H	-4.390718	-1.797493	-0.098702
H	-2.890295	-2.667155	0.289345
C	-3.224653	0.626928	-0.750503
H	-2.778344	1.581521	-0.453070
H	-4.308159	0.696166	-0.614703
H	-3.024573	0.465024	-1.814230
C	-2.929109	-0.242844	1.575175
H	-2.520980	-1.036842	2.208138
H	-4.009296	-0.196912	1.736795
H	-2.503715	0.716204	1.890513
C	0.002269	2.276867	-0.448594
H	-0.026073	1.587032	-1.309416
N	1.192405	-0.622570	-0.126497
N	-1.197364	-0.618086	-0.127185
O	0.003864	1.685605	0.747217
O	0.028990	3.476346	-0.580476
P	-0.001858	-0.095181	0.962885

[Si]H$_2$

M06-2X/BS1 SCF energy in solution :
-753.770148 a.u.
M06-2X/BS2 SCF energy in solution:
-753.909651 a.u.

M06-2X/BS2 Free energy in solution:
-753.748736 a.u.

Si	-0.000022	1.578871	0.000048
H	-0.108491	2.454113	1.195280
H	0.108436	2.454162	-1.195149
C	-1.539676	0.507309	-0.060975
C	-1.573420	-0.656032	-0.846126
C	-2.689080	0.850855	0.665417
C	-2.719253	-1.445621	-0.909530
H	-0.692507	-0.955931	-1.410152
C	-3.837952	0.063238	0.605763
H	-2.689494	1.741195	1.290066
C	-3.853780	-1.085254	-0.183155
H	-2.727119	-2.342336	-1.521658
H	-4.717839	0.343409	1.176889
H	-4.746919	-1.700933	-0.229119
C	1.539661	0.507348	0.061015
C	2.688924	0.850764	-0.665662
C	1.573571	-0.655834	0.846397
C	3.837817	0.063172	-0.606069
H	2.689206	1.740980	-1.290485
C	2.719426	-1.445395	0.909742
H	0.692770	-0.955634	1.410652
C	3.853810	-1.085160	0.183078
H	4.717591	0.343239	-1.177420
H	2.727422	-2.341985	1.522051
H	4.746965	-1.700818	0.228996

TS4

M06-2X/BS1 SCF energy in solution :
-1786.607434 a.u.
M06-2X/BS2 SCF energy in solution:
-1786.968641 a.u.
M06-2X/BS2 Free energy in solution:
-1786.512986 a.u.

C	3.639255	1.398036	-0.096607
C	4.202995	0.228317	0.288934
H	3.989899	2.397583	0.116598
H	5.101799	0.091509	0.872476
P	2.091458	-0.424797	-0.996176
N	2.495765	1.188960	-0.840688
N	3.474601	-0.855564	-0.163410
C	1.619719	2.316045	-1.290061
C	3.875126	-2.262621	0.152778
C	0.498979	1.743767	-2.154499
H	-0.116039	1.036587	-1.585838
H	-0.150204	2.562247	-2.475867
H	0.894329	1.248387	-3.047454
C	1.028864	2.977441	-0.043465
H	0.464812	2.240567	0.535877
H	1.816004	3.403057	0.586393
H	0.356485	3.786266	-0.345015
C	2.462119	3.298057	-2.102153
H	3.245741	3.759003	-1.494807
H	2.925269	2.795197	-2.956023
H	1.815600	4.096627	-2.475792
C	2.882749	-3.220393	-0.501234
H	1.867037	-3.068380	-0.118637
H	2.878838	-3.115387	-1.590910
H	3.178585	-4.244491	-0.261509
C	3.847040	-2.443477	1.671309
H	2.838178	-2.279550	2.061484
H	4.151620	-3.464871	1.915515
H	4.534855	-1.754268	2.169046
C	5.277931	-2.496996	-0.406977
H	5.293552	-2.318839	-1.486053
H	6.015165	-1.844998	0.069530
H	5.570516	-3.533284	-0.218434
O	0.411757	-0.307496	0.881041
C	0.926854	-0.181834	2.053239
H	1.989112	0.159564	2.038553
O	0.387531	-0.391614	3.135892
C	-2.084417	0.924160	0.653017
C	-2.618968	1.687286	-0.395685
C	-1.864492	1.563420	1.882173
C	-2.890691	3.046675	-0.240308
H	-2.822089	1.216446	-1.356859
C	-2.157229	2.916612	2.052352
H	-1.434212	0.997352	2.704477
C	-2.661526	3.664341	0.988529
H	-3.286882	3.620457	-1.073285
H	-1.984412	3.391727	3.014036
H	-2.879238	4.720501	1.117398
C	-3.428638	-1.528237	-0.223506
C	-3.637900	-1.975507	-1.535599
C	-4.540248	-1.534676	0.636957

C	-4.891022	-2.408527	-1.975860
H	-2.802767	-1.988249	-2.235079
C	-5.795511	-1.965982	0.213489
H	-4.424660	-1.192836	1.665819
C	-5.973842	-2.404387	-1.099940
H	-5.021615	-2.748616	-2.999578
H	-6.635639	-1.961071	0.902567
H	-6.950491	-2.740812	-1.435371
Si	-1.701626	-0.907836	0.378906
H	-0.927491	-1.250582	-0.845678
H	-1.530506	-1.725178	1.602302

IM4

M06-2X/BS1 SCF energy in solution :
-1786.608088 a.u.
M06-2X/BS2 SCF energy in solution:
-1786.968254 a.u.
M06-2X/BS2 Free energy in solution:
-1786.514400 a.u.

C	3.540001	1.448582	0.095736
C	4.146165	0.283357	0.446783
H	3.806737	2.447008	0.411951
H	4.997191	0.159049	1.101101
P	2.218488	-0.384205	-1.087319
N	2.483297	1.231631	-0.752581
N	3.534268	-0.797389	-0.141316
C	1.592285	2.344087	-1.218516
C	3.986929	-2.203457	0.116091
C	0.518791	1.755507	-2.129550
H	-0.086825	1.013345	-1.596428
H	-0.146805	2.560478	-2.450964
H	0.955128	1.297571	-3.023765
C	0.940304	2.967857	0.016689
H	0.399540	2.200633	0.577937
H	1.686048	3.432998	0.667990
H	0.234667	3.740851	-0.301891
C	2.442636	3.356997	-1.982828
H	3.202481	3.811416	-1.340987
H	2.936347	2.881056	-2.834919
H	1.795204	4.155353	-2.355298
C	3.121746	-3.160398	-0.699257
H	2.067484	-3.097947	-0.406951
H	3.212478	-2.971904	-1.773965
H	3.460337	-4.181048	-0.506788
C	3.814450	-2.491554	1.607516
H	2.763256	-2.395103	1.895687
H	4.139259	-3.515350	1.811642
H	4.414700	-1.814187	2.221293
C	5.448943	-2.319938	-0.312334
H	5.560017	-2.061604	-1.369304
H	6.095430	-1.669066	0.282169
H	5.781334	-3.351081	-0.166987
O	0.277395	-0.331148	0.924931
C	0.764302	-0.211065	2.114344
H	1.852050	0.028308	2.109795
O	0.178939	-0.328745	3.183800
C	-2.139901	0.911903	0.623984
C	-2.675738	1.608195	-0.470397
C	-2.004580	1.610446	1.833711
C	-3.031019	2.953882	-0.377593
H	-2.814797	1.093054	-1.420184
C	-2.383579	2.949070	1.943052
H	-1.575597	1.102189	2.693590
C	-2.888785	3.628019	0.834881
H	-3.425954	3.472844	-1.246548
H	-2.276904	3.466272	2.892737
H	-3.173156	4.673105	0.915779
C	-3.335801	-1.584343	-0.218097
C	-3.568749	-1.883681	-1.568323
C	-4.411657	-1.782013	0.664290
C	-4.805641	-2.347048	-2.023991
H	-2.760366	-1.754544	-2.288547
C	-5.652346	-2.245190	0.228931
H	-4.277185	-1.568916	1.725747
C	-5.852796	-2.529142	-1.123132
H	-4.951752	-2.569374	-3.077849
H	-6.463092	-2.387883	0.938580
H	-6.816515	-2.891634	-1.469015
Si	-1.613685	-0.903998	0.419786
H	-0.888598	-1.247949	-0.844786
H	-1.557256	-1.759871	1.637439

TS5

M06-2X/BS1 SCF energy in solution :
-1786.602552 a.u.
M06-2X/BS2 SCF energy in solution:
-1786.958506 a.u.

M06-2X/BS2 Free energy in solution:
-1786.501714 a.u.

H	0.000505	-1.284451	3.382397
C	-0.596000	-0.593299	2.763833
O	-1.750255	-0.326143	3.029097
O	0.087229	-0.131309	1.740792
Si	-0.463120	1.286417	0.793977
H	-0.706698	2.056825	2.093897
H	-0.126321	0.274464	-0.464097
C	0.871094	2.486941	0.136796
C	1.296728	2.464082	-1.200268
C	1.455274	3.439883	0.984331
C	2.257241	3.355052	-1.676415
H	0.871323	1.726903	-1.880158
C	2.433126	4.322901	0.525330
H	1.142342	3.488517	2.025350
C	2.833648	4.283345	-0.809025
H	2.563166	3.321398	-2.718276
H	2.879697	5.042402	1.205759
H	3.591772	4.971093	-1.171878
C	-2.224921	1.530521	0.098728
C	-2.480794	1.484667	-1.279707
C	-3.305572	1.791391	0.952730
C	-3.759719	1.699316	-1.791176
H	-1.660839	1.275289	-1.965806
C	-4.594303	1.984451	0.454262
H	-3.132597	1.823792	2.025815
C	-4.822328	1.942514	-0.920502
H	-3.930850	1.670751	-2.863539
H	-5.419972	2.169534	1.135617
H	-5.823404	2.097196	-1.312258
C	1.625280	-2.664140	0.938800
C	0.353860	-3.094354	0.787370
H	2.311448	-2.913901	1.734729
H	-0.191330	-3.768704	1.432261
N	2.001142	-1.813654	-0.086912
N	-0.235884	-2.560274	-0.344045
P	0.746307	-1.456991	-1.143372
C	-1.657560	-2.843454	-0.695148
C	3.363635	-1.206488	-0.136007
C	-1.842607	-4.356935	-0.806081
H	-1.664041	-4.859009	0.148504
H	-2.871128	-4.570461	-1.109719
H	-1.163407	-4.773008	-1.555832
C	-2.548613	-2.255307	0.399248
H	-2.406184	-1.173037	0.471505
H	-3.597694	-2.455635	0.162219
H	-2.325502	-2.699344	1.373860
C	-1.976047	-2.185411	-2.035190
H	-1.851102	-1.097907	-1.984165
H	-1.349100	-2.586540	-2.837898
H	-3.021025	-2.384438	-2.285736
C	4.399720	-2.325857	-0.015937
H	5.400127	-1.896720	-0.117351
H	4.348458	-2.828707	0.953019
H	4.256852	-3.067790	-0.807084
C	3.492133	-0.210213	1.016696
H	4.496508	0.223235	1.013830
H	2.760364	0.595781	0.907228
H	3.330693	-0.701335	1.980873
C	3.544438	-0.490105	-1.471756
H	3.433139	-1.181605	-2.313038
H	2.834257	0.335716	-1.585218
H	4.550747	-0.065173	-1.507786

H[Si](OCHO)

M06-2X/BS1 SCF energy in solution :
-942.319982 a.u.
M06-2X/BS2 SCF energy in solution:
-942.521488 a.u.
M06-2X/BS2 Free energy in solution:
-942.347106 a.u.

H	-0.889652	3.784167	1.314740
C	-1.007701	2.998542	0.556301
O	-1.954489	2.915565	-0.187209
O	0.022685	2.151448	0.555232
Si	0.014840	0.831566	-0.546573
H	-0.149308	1.388663	-1.905980
C	-1.368857	-0.356226	-0.167323
C	-2.600814	-0.249685	-0.830973
C	-1.209423	-1.369861	0.790188
C	-3.643728	-1.126942	-0.544668
H	-2.747936	0.530456	-1.573526
C	-2.252538	-2.246160	1.082487
H	-0.260639	-1.481230	1.310281
C	-3.469646	-2.124480	0.414115

H	-4.590865	-1.033453	-1.066911
H	-2.115627	-3.024012	1.827216
H	-4.282547	-2.808311	0.638928
C	1.688041	0.060029	-0.268962
C	1.937656	-1.253077	-0.698270
C	2.732169	0.774834	0.336330
C	3.192718	-1.833600	-0.530693
H	1.143090	-1.835411	-1.160442
C	3.987910	0.195504	0.507663
H	2.562224	1.790659	0.682736
C	4.218827	-1.108546	0.073416
H	3.368805	-2.850933	-0.866050
H	4.784832	0.760369	0.981393
H	5.196617	-1.560986	0.207788

TS6

M06-2X/BS1 SCF energy in solution :
-1975.154262 a.u.
M06-2X/BS2 SCF energy in solution:
-1975.577337 a.u.
M06-2X/BS2 Free energy in solution:
-1975.107422 a.u.

C	-4.268869	0.230866	-0.342749
C	-3.997327	-1.051967	-0.651877
H	-5.056966	0.852452	-0.741859
H	-4.520987	-1.685088	-1.353419
P	-2.161709	-0.356088	1.000762
N	-3.379761	0.724373	0.611486
N	-2.903879	-1.531621	0.064092
C	-3.446854	2.135397	1.091031
C	-2.341521	-2.892249	-0.177201
C	-2.450852	2.320094	2.234058
H	-1.421035	2.125183	1.913797
H	-2.499307	3.356101	2.577937
H	-2.689128	1.665934	3.078747
C	-3.089723	3.058561	-0.075196
H	-2.074749	2.852101	-0.430183
H	-3.786207	2.925089	-0.908471
H	-3.140079	4.101251	0.251194
C	-4.861254	2.415988	1.600372
H	-5.603451	2.355234	0.800132
H	-5.131186	1.706612	2.388008
H	-4.897895	3.427571	2.013698
C	-1.229046	-3.146628	0.837437
H	-0.439283	-2.391908	0.751474
H	-1.611346	-3.140667	1.863070
H	-0.780013	-4.123193	0.639353
C	-1.773654	-2.940759	-1.597989
H	-0.941983	-2.238272	-1.708519
H	-1.400619	-3.947094	-1.807590
H	-2.546604	-2.700092	-2.334488
C	-3.453350	-3.925312	0.005935
H	-3.903931	-3.833624	0.998415
H	-4.236760	-3.815882	-0.748939
H	-3.030639	-4.928684	-0.094935
O	-0.591070	0.197263	-0.423358
C	-0.954732	0.433120	-1.663188
H	-2.048396	0.568613	-1.783851
O	-0.211709	0.509709	-2.620170
C	1.829732	1.620225	-0.565756
C	0.970974	2.690648	-0.278001
C	2.963952	1.883113	-1.346668
C	1.225683	3.973053	-0.758926
H	0.082961	2.514689	0.322240
C	3.211898	3.157884	-1.854124
H	3.667547	1.084148	-1.569042
C	2.344465	4.207481	-1.557415
H	0.548203	4.786844	-0.516398
H	4.087169	3.333491	-2.472488
H	2.541385	5.203529	-1.942467
C	3.301072	-0.508506	0.820691
C	4.320920	-1.076406	0.038388
C	3.620429	-0.173941	2.144717
C	5.599815	-1.292260	0.549517
H	4.117024	-1.360146	-0.993451
C	4.896693	-0.383895	2.668006
H	2.854448	0.257121	2.788700
C	5.890793	-0.944223	1.868412
H	6.368664	-1.733490	-0.078261
H	5.113940	-0.116852	3.698250
H	6.885222	-1.113055	2.270677
Si	1.547827	-0.088124	0.164824
H	0.800754	-0.162136	1.443902
O	1.377790	-1.384028	-0.960474
C	1.695479	-2.652017	-0.709796
H	2.044926	-2.852906	0.316648
O	1.595177	-3.520030	-1.540741

IM5

M06-2X/BS1 SCF energy in solution :
-1975.159819 a.u.
M06-2X/BS2 SCF energy in solution:
-1975.582589 a.u.
M06-2X/BS2 Free energy in solution:
-1975.112476 a.u.

C	-4.314124	0.262909	-0.606284
C	-3.935839	-1.002733	-0.933366
H	-5.029647	0.887342	-1.122297
H	-4.294770	-1.599979	-1.759607
P	-2.566750	-0.398258	1.133890
N	-3.650531	0.711615	0.507726
N	-2.990207	-1.484656	-0.066076
C	-3.832293	2.104824	1.032397
C	-2.357204	-2.832738	-0.246693
C	-2.993809	2.272547	2.296401
H	-1.925070	2.139015	2.094496
H	-3.136008	3.288106	2.672978
H	-3.304458	1.574867	3.080988
C	-3.350627	3.070021	-0.050903
H	-2.299186	2.878921	-0.288511
H	-3.943189	2.970398	-0.964425
H	-3.447853	4.096634	0.312061
C	-5.311691	2.309314	1.353113
H	-5.934881	2.226552	0.459123
H	-5.648794	1.575299	2.090429
H	-5.449083	3.311037	1.768334
C	-1.318461	-3.029448	0.853351
H	-0.566065	-2.232696	0.824650
H	-1.778315	-3.055045	1.846903
H	-0.809037	-3.982348	0.690058
C	-1.674403	-2.858803	-1.614736
H	-0.886505	-2.102060	-1.666749
H	-1.214013	-3.839421	-1.760439
H	-2.391295	-2.694106	-2.423927
C	-3.452067	-3.892231	-0.143566
H	-3.961136	-3.824429	0.822051
H	-4.190939	-3.784281	-0.942465
H	-2.998370	-4.882860	-0.232375
O	-0.262563	0.260427	-0.451869
C	-0.617071	0.507748	-1.683061
H	-1.705302	0.711390	-1.770723
O	0.100417	0.533142	-2.664604
C	2.049556	1.664431	-0.523127
C	1.210298	2.776605	-0.344833
C	3.290620	1.889757	-1.136092
C	1.590389	4.053205	-0.754761
H	0.237320	2.639170	0.120123
C	3.666435	3.157003	-1.581671
H	3.981821	1.061735	-1.272756
C	2.818891	4.245374	-1.385365
H	0.925407	4.896285	-0.589587
H	4.625166	3.296041	-2.073019
H	3.114631	5.236166	-1.717456
C	3.297380	-0.550925	0.810421
C	4.297858	-1.229525	0.092796
C	3.630280	-0.139505	2.109585
C	5.559382	-1.477243	0.635675
H	4.093229	-1.576720	-0.919705
C	4.886163	-0.379020	2.668921
H	2.883154	0.381041	2.710007
C	5.858052	-1.050916	1.929300
H	6.309102	-2.004339	0.051670
H	5.105930	-0.048439	3.680538
H	6.837024	-1.244362	2.357912
Si	1.530378	-0.053370	0.117626
H	0.889964	-0.106002	1.471271
O	1.449850	-1.448863	-0.949457
C	1.682640	-2.690686	-0.564438
H	1.898409	-2.814679	0.511210
O	1.648766	-3.635416	-1.319794

TS7

M06-2X/BS1 SCF energy in solution :
-1975.164971 a.u.
M06-2X/BS2 SCF energy in solution:
-1975.584713 a.u.
M06-2X/BS2 Free energy in solution:
-1975.114413 a.u.

H	-1.396476	-4.244340	2.364804
C	-1.131887	-3.679971	1.453713
O	-1.111600	-4.201832	0.358489
O	-0.857854	-2.423442	1.721899
Si	-0.368193	-1.241418	0.439464

H	0.098886	0.023192	-0.557351
C	-2.003866	-1.296088	-0.512953
C	-2.038226	-0.926900	-1.865503
C	-3.215465	-1.672609	0.089947
C	-3.226081	-0.946963	-2.596259
H	-1.120958	-0.609327	-2.358398
C	-4.412672	-1.659630	-0.622967
H	-3.227470	-1.977158	1.133362
C	-4.419372	-1.302130	-1.970841
H	-3.221844	-0.670608	-3.646537
H	-5.339645	-1.936168	-0.129266
H	-5.350125	-1.301113	-2.530075
C	1.206528	-2.143756	-0.073654
C	1.691023	-2.044071	-1.384655
C	1.974499	-2.857902	0.857710
C	2.896931	-2.633924	-1.759257
H	1.123774	-1.481039	-2.124555
C	3.196975	-3.425010	0.499309
H	1.620209	-2.962742	1.880264
C	3.659804	-3.315031	-0.811224
H	3.248391	-2.549322	-2.783533
H	3.786950	-3.955067	1.241317
H	4.609986	-3.758740	-1.092909
C	0.254715	2.965127	1.217427
C	1.556395	2.629200	1.131820
H	-0.254536	3.409281	2.059987
H	2.318711	2.760986	1.886029
N	-0.434909	2.656719	0.049628
N	1.863822	2.061380	-0.100006
P	0.503106	1.802686	-1.052245
C	3.258617	1.675166	-0.454956
C	-1.863107	3.040653	-0.135751
C	4.159016	2.901013	-0.286846
H	4.198702	3.234556	0.753438
H	5.176092	2.645235	-0.596658
H	3.801396	3.727065	-0.908552
C	3.707671	0.539063	0.465944
H	3.057085	-0.331556	0.344079
H	4.732684	0.247399	0.218369
H	3.684510	0.850926	1.514444
C	3.294099	1.217610	-1.910970
H	2.646619	0.350370	-2.075694
H	2.996069	2.023661	-2.589024
H	4.315173	0.918840	-2.161992
C	-2.005669	4.534631	0.164751
H	-3.034575	4.843546	-0.038731
H	-1.788726	4.760607	1.211982
H	-1.333415	5.119982	-0.469451
C	-2.728706	2.213846	0.814985
H	-3.772158	2.532522	0.732653
H	-2.672294	1.151413	0.555733
H	-2.410435	2.347354	1.853242
C	-2.272895	2.774229	-1.581682
H	-1.672228	3.363306	-2.281800
H	-2.185886	1.712356	-1.835226
H	-3.321147	3.057365	-1.707417
O	-0.145753	-0.070656	1.702055
C	-0.998875	0.166063	2.688856
H	-1.956507	-0.375577	2.611912
O	-0.758267	0.929676	3.593521

[Si](OCHO)$_2$

M06-2X/BS1 SCF energy in solution :

-1130.862460 a.u.

M06-2X/BS2 SCF energy in solution:

-1131.128211 a.u.

M06-2X/BS2 Free energy in solution:

-1130.943678 a.u.

Si	0.000178	0.478808	-0.000115
C	1.558634	-0.504303	-0.118879
C	2.641209	-0.067963	-0.898378
C	1.680613	-1.703401	0.600884
C	3.817771	-0.810714	-0.952589
H	2.562979	0.853902	-1.468524
C	2.857146	-2.446242	0.545784
H	0.849341	-2.065909	1.201828
C	3.925068	-1.998713	-0.230617
H	4.648879	-0.465322	-1.559236
H	2.939874	-3.373511	1.103658
H	4.841992	-2.578314	-0.275400
C	-1.558245	-0.504247	0.119589
C	-2.639808	-0.068689	0.900902
C	-1.681350	-1.702329	-0.601689
C	-3.816507	-0.811216	0.955434
H	-2.560685	0.852422	1.472133
C	-2.858012	-2.444930	-0.546284
H	-0.850847	-2.064238	-1.204070

C	-3.924946	-1.998172	0.231934
H	-4.646847	-0.466425	1.563475
H	-2.941634	-3.371399	-1.105354
H	-4.841988	-2.577570	0.276922
O	-0.023173	1.500215	1.344526
O	0.023182	1.498422	-1.346196
C	1.040362	2.103807	1.910649
H	2.011663	1.815047	1.479415
C	-1.040760	2.101934	-1.911633
H	-2.011580	1.815065	-1.478050
O	0.916969	2.880978	2.814310
O	-0.918315	2.877134	-2.817119

[NHP]$^+$

M06-2X/BS1 SCF energy in solution :
-843.603412 a.u.
M06-2X/BS2 SCF energy in solution:
-843.752167 a.u.
M06-2X/BS2 Free energy in solution:
-843.496873 a.u.

C	-0.682943	1.462481	0.015801
C	0.682943	1.462481	0.015800
H	-1.333621	2.325641	0.012597
H	1.333621	2.325641	0.012597
N	-1.184226	0.195256	0.017412
N	1.184226	0.195256	0.017411
P	0.000000	-0.995914	0.021955
C	2.663013	-0.073191	-0.004457
C	-2.663013	-0.073191	-0.004457
C	2.894845	-1.579759	0.044503
H	2.463540	-2.084518	-0.826299
H	3.972057	-1.761070	0.033940
H	2.489769	-2.021142	0.961025
C	3.221290	0.505004	-1.304546
H	3.072683	1.586727	-1.358062
H	4.294911	0.304748	-1.348741
H	2.740696	0.036640	-2.168114
C	3.284047	0.594544	1.221397
H	3.160843	1.680234	1.195760
H	2.833562	0.205703	2.138777
H	4.354684	0.375129	1.236397
C	-2.894845	-1.579760	0.044481
H	-3.972057	-1.761071	0.033919
H	-2.463543	-2.084506	-0.826330
H	-2.489766	-2.021156	0.960995
C	-3.221292	0.505023	-1.304536
H	-4.294913	0.304770	-1.348732
H	-3.072683	1.586747	-1.358036
H	-2.740701	0.036672	-2.168112
C	-3.284044	0.594526	1.221408
H	-2.833557	0.205672	2.138782
H	-3.160842	1.680216	1.195787
H	-4.354681	0.375109	1.236407

HCO_2^-

M06-2X/BS1 SCF energy in solution :
-189.195715 a.u.
M06-2X/BS2 SCF energy in solution:
-189.286441 a.u.
M06-2X/BS2 Free energy in solution:
-189.288620 a.u.

H	0.000000	0.000000	1.455418
C	0.000000	0.000000	0.316163
O	0.000000	1.131672	-0.209525
O	0.000000	-1.131672	-0.209525

IM6$^-$

M06-2X/BS1 SCF energy in solution :
-942.987975 a.u.
M06-2X/BS2 SCF energy in solution:
-943.199042 a.u.
M06-2X/BS2 Free energy in solution:
-943.018213 a.u.

H	0.485721	3.705221	-0.999711
C	0.785496	2.939040	-0.253446
O	1.678592	3.184630	0.546522
O	0.107942	1.846102	-0.367871
Si	-0.041903	0.513335	1.029784
H	0.020027	1.667392	1.984162
H	-0.189325	-0.520435	2.194193
C	1.483066	-0.425711	0.338035
C	1.529113	-1.816884	0.523944
C	2.547134	0.155640	-0.369122
C	2.566985	-2.597882	0.016984
H	0.728259	-2.301783	1.079606

C	3.606897	-0.610870	-0.855413
H	2.563173	1.229776	-0.522276
C	3.616497	-1.992573	-0.671912
H	2.562155	-3.673958	0.167083
H	4.425375	-0.129099	-1.383428
H	4.436059	-2.591488	-1.058577
C	-1.742774	0.003556	0.288239
C	-2.587230	-0.831695	1.038638
C	-2.202977	0.381324	-0.986316
C	-3.827307	-1.256568	0.561319
H	-2.265360	-1.159069	2.024094
C	-3.432263	-0.051937	-1.482068
H	-1.583992	1.030554	-1.595309
C	-4.253373	-0.869830	-0.707134
H	-4.456419	-1.893669	1.176823
H	-3.752696	0.253394	-2.474518
H	-5.214188	-1.203037	-1.088808

TS8

M06-2X/BS1 SCF energy in solution :
-1786.605886 a.u.
M06-2X/BS2 SCF energy in solution:
-1786.964415 a.u.
M06-2X/BS2 Free energy in solution:
-1786.506538 a.u.

H	3.384304	-2.721743	3.098618
C	2.420140	-2.346906	2.698019
O	1.415451	-2.373274	3.394218
O	2.525725	-1.924066	1.478981
Si	1.248274	-0.816293	0.596798
H	0.937706	-0.197288	1.922594
H	0.185306	0.127029	-0.132597
C	0.345754	-2.345753	-0.107081
C	-0.090614	-2.311620	-1.441019
C	0.027220	-3.485648	0.645529
C	-0.808355	-3.364010	-2.007132
H	0.125018	-1.433347	-2.049178
C	-0.722256	-4.529579	0.099186
H	0.345981	-3.545980	1.682657
C	-1.138422	-4.474329	-1.229812
H	-1.122678	-3.312603	-3.046086
H	-0.978148	-5.390227	0.711192
H	-1.715639	-5.289025	-1.657186
C	2.659229	-0.053155	-0.455498
C	2.339267	0.647608	-1.631215
C	4.019563	-0.115223	-0.107699
C	3.316242	1.249185	-2.424536
H	1.296726	0.730442	-1.931605
C	5.003628	0.497869	-0.883247
H	4.309731	-0.656448	0.786257
C	4.655914	1.180530	-2.047523
H	3.030652	1.776414	-3.330570
H	6.045094	0.440137	-0.579155
H	5.421452	1.655015	-2.654449
C	-2.663690	1.108725	1.413295
C	-1.782114	2.112249	1.670872
H	-3.311636	0.611576	2.121173
H	-1.575198	2.567813	2.628800
N	-2.686056	0.784657	0.079760
N	-1.152699	2.539847	0.529708
P	-1.567164	1.643127	-0.821554
C	-0.100898	3.605418	0.554664
C	-3.594499	-0.277665	-0.460974
C	-0.703641	4.859136	1.187230
H	-0.983062	4.692349	2.230703
H	0.040342	5.659531	1.161572
H	-1.587158	5.183443	0.630054
C	1.085188	3.083581	1.366733
H	1.506978	2.191367	0.893338
H	1.857821	3.856236	1.407842
H	0.789117	2.837580	2.390492
C	0.337414	3.904066	-0.876230
H	0.793207	3.026911	-1.349256
H	-0.499211	4.256123	-1.488463
H	1.093466	4.692407	-0.848367
C	-5.036431	0.164367	-0.213406
H	-5.715799	-0.587993	-0.623067
H	-5.245687	0.265307	0.855163
H	-5.234608	1.120999	-0.705251
C	-3.282655	-1.590318	0.258627
H	-3.891250	-2.387862	-0.176553
H	-2.226657	-1.847923	0.140416
H	-3.516173	-1.529099	1.325163
C	-3.339877	-0.428287	-1.957803
H	-3.561880	0.496592	-2.500427
H	-2.305771	-0.728933	-2.158090
H	-3.995488	-1.211838	-2.345341

IM7⁻

M06-2X/BS1 SCF energy in solution :
-1131.546945 a.u.
M06-2X/BS2 SCF energy in solution:
-1131.822771 a.u.
M06-2X/BS2 Free energy in solution:
-1131.629339 a.u.

H	-0.114864	-2.575011	-2.229757
C	-0.201870	-3.066701	-1.246585
O	-0.125422	-2.251255	-0.211417
O	-0.353160	-4.262797	-1.134472
Si	0.083375	-0.484916	-0.303689
H	0.164650	-0.587309	-1.834638
C	-1.469535	0.631864	-0.374536
C	-2.217998	0.688532	-1.560065
C	-1.893435	1.450421	0.687733
C	-3.345686	1.500486	-1.682213
H	-1.911731	0.080070	-2.408719
C	-3.004363	2.283744	0.570146
H	-1.341361	1.441233	1.623851
C	-3.738997	2.305188	-0.615164
H	-3.912040	1.510190	-2.609079
H	-3.300475	2.914176	1.403757
H	-4.610617	2.946686	-0.706030
C	1.803521	0.341863	-0.179612
C	2.566591	0.478306	-1.350333
C	2.353947	0.868759	1.003276
C	3.815472	1.099727	-1.351146
H	2.174566	0.085737	-2.285808
C	3.596671	1.499365	1.012889
H	1.797525	0.776160	1.929633
C	4.333618	1.615079	-0.165605
H	4.381214	1.182468	-2.274803
H	3.993492	1.899856	1.941613
H	5.304131	2.102697	-0.158472
O	0.015688	-0.586356	1.533171
C	-0.962407	-1.151903	2.186675
H	-1.774165	-1.562446	1.556541
O	-1.015951	-1.236351	3.399066

1a

M06-2X/BS1 SCF energy in solution :
-326.777906 a.u.
M06-2X/BS2 SCF energy in solution:
-326.862226 a.u.
M06-2X/BS2 Free energy in solution:
-326.746502 a.u.

C	1.348251	1.351630	0.011246
C	-0.013309	1.054923	0.056403
C	-0.446451	-0.281773	0.061093
C	0.525390	-1.300427	0.006515
C	1.876187	-0.988773	-0.042698
C	2.304361	0.341493	-0.039225
H	1.658585	2.392766	0.009743
H	-0.736716	1.862308	0.088143
H	0.199429	-2.337503	0.005751
H	2.604189	-1.793989	-0.085218
H	3.361660	0.581375	-0.077215
N	-1.785244	-0.618891	0.148922
H	-1.999513	-1.548494	-0.187297
C	-2.811923	0.370854	-0.105004
H	-2.686791	0.880932	-1.071167
H	-3.782802	-0.125586	-0.096022
H	-2.816371	1.132863	0.680850

IM8⁻

M06-2X/BS1 SCF energy in solution :
-515.993812 a.u.
M06-2X/BS2 SCF energy in solution:
-516.158119 a.u.
M06-2X/BS2 Free energy in solution:
-516.028768 a.u.

H	-1.310630	0.799156	-0.023832
N	-0.403366	1.222474	-0.279515
C	-0.324017	2.650957	-0.090687
H	-1.333370	3.062533	-0.154004
H	0.103549	2.937232	0.883044
H	0.282888	3.128936	-0.869394
H	-4.429125	-0.973883	0.352308
C	-3.347598	-0.743453	0.137087
O	-2.969467	0.394033	0.515023
O	-2.700341	-1.639517	-0.434927
C	0.706723	0.430295	-0.120399
C	2.008699	0.957375	-0.014945

C	0.547877	-0.974823	-0.084985
C	3.105345	0.105133	0.113250
H	2.163741	2.031130	-0.034343
C	1.651390	-1.803108	0.048694
H	-0.461438	-1.376879	-0.174673
C	2.944804	-1.276828	0.148543
H	4.099662	0.537138	0.190763
H	1.504064	-2.879864	0.074926
H	3.803338	-1.932228	0.251688

TS9^{-}

M06-2X/BS1 SCF energy in solution :
-1646.869078 a.u.
M06-2X/BS2 SCF energy in solution:
-1647.290395 a.u.
M06-2X/BS2 Free energy in solution:
-1646.945369 a.u.

C	1.037775	-1.315095	-0.188973
O	0.532372	-2.226228	0.508556
O	0.228933	-0.182638	-0.449009
H	3.166949	-1.268187	0.543270
H	1.628189	-1.537805	-1.091574
Si	-1.272776	-0.057182	0.230547
N	2.385827	-0.549713	0.707170
C	2.051421	-0.560530	2.131944
H	1.860758	-1.596344	2.410472
H	2.878984	-0.156975	2.721315
H	1.144902	0.016733	2.324744
C	-1.691755	1.749616	0.085291
C	-2.732715	2.327123	0.829015
C	-0.941009	2.576498	-0.763848
C	-3.021866	3.685545	0.720808
H	-3.323004	1.712963	1.505963
C	-1.224076	3.937191	-0.871263
H	-0.118488	2.152278	-1.334524
C	-2.266684	4.491434	-0.130907
H	-3.830496	4.116737	1.302997
H	-0.628530	4.564029	-1.528282
H	-2.488794	5.551168	-0.213032
H	4.869851	-3.875993	-0.786070
C	4.277047	-2.928766	-0.707290
O	4.242215	-2.425722	0.450864
O	3.750240	-2.506013	-1.750757
C	2.740316	0.706818	0.129525
C	2.367689	1.924775	0.701760
C	3.420293	0.685132	-1.093468
C	2.672062	3.116754	0.045869
H	1.840120	1.952460	1.649049
C	3.716335	1.882449	-1.738350
H	3.694756	-0.277924	-1.521591
C	3.343161	3.103145	-1.174940
H	2.375868	4.060393	0.494419
H	4.242929	1.861600	-2.687894
H	3.575624	4.034286	-1.681857
C	-2.542792	-1.172924	-0.568432
C	-2.268261	-2.513287	-0.892456
C	-3.815032	-0.663984	-0.875176
C	-3.236572	-3.314334	-1.493376
H	-1.288242	-2.922629	-0.663085
C	-4.785393	-1.465395	-1.474442
H	-4.055456	0.372659	-0.651972
C	-4.496670	-2.792601	-1.783596
H	-3.007525	-4.347546	-1.736629
H	-5.763119	-1.052404	-1.703037
H	-5.250131	-3.418412	-2.252457
O	-1.241604	-0.320033	1.906091
C	-1.239160	-1.523886	2.504705
H	-1.632451	-2.342724	1.887820
O	-0.882067	-1.659852	3.644219

IM9^{-}

M06-2X/BS1 SCF energy in solution :
-1646.896038 a.u.
M06-2X/BS2 SCF energy in solution:
-1647.308833 a.u.
M06-2X/BS2 Free energy in solution:
-1646.963721 a.u.

C	1.036818	-0.469561	0.063242
O	0.108254	-0.818238	1.045319
O	0.271255	0.289655	-0.796209
H	2.605222	-0.691772	1.891451
H	1.480208	-1.355236	-0.410542
Si	-1.207130	-0.050549	0.202421
N	2.165242	0.281294	0.676663
C	1.709990	1.489510	1.369892
H	1.001062	1.188682	2.143061

H	2.567397	1.969610	1.846383
H	1.218411	2.200635	0.698168
C	-1.785361	1.762002	0.304207
C	-2.828372	2.195981	1.140407
C	-1.207004	2.724133	-0.543715
C	-3.265395	3.520773	1.138618
H	-3.302227	1.484045	1.807492
C	-1.641672	4.048591	-0.556958
H	-0.391403	2.424210	-1.195102
C	-2.673756	4.452465	0.288343
H	-4.069732	3.825456	1.802347
H	-1.173024	4.766600	-1.224146
H	-3.012871	5.484316	0.285006
H	2.886704	-3.153263	3.341032
C	2.750750	-2.528476	2.443565
O	2.815564	-1.250308	2.731800
O	2.575989	-3.003744	1.339480
C	3.247037	0.469376	-0.244189
C	3.602065	1.724244	-0.745280
C	3.982046	-0.658701	-0.634032
C	4.676254	1.844858	-1.627045
H	3.048566	2.609735	-0.453476
C	5.042987	-0.530237	-1.523366
H	3.715835	-1.628390	-0.221177
C	5.398021	0.723002	-2.023602
H	4.943802	2.826483	-2.006272
H	5.604691	-1.412352	-1.815565
H	6.231681	0.822685	-2.711252
C	-2.133857	-1.060307	-1.118157
C	-1.493585	-1.473745	-2.297693
C	-3.490761	-1.400002	-0.974147
C	-2.164928	-2.204212	-3.278353
H	-0.450680	-1.209599	-2.445085
C	-4.174977	-2.115784	-1.955168
H	-4.027053	-1.097113	-0.078219
C	-3.510214	-2.525398	-3.110323
H	-1.639490	-2.518228	-4.175886
H	-5.225055	-2.357533	-1.817636
H	-4.038262	-3.088772	-3.874164
O	-2.300395	-0.528593	1.567826
C	-2.369294	-1.756608	2.018227
H	-1.744907	-2.489748	1.474914
O	-3.061482	-2.095428	2.956945

TS10$^-$

M06-2X/BS1 SCF energy in solution :
-1646.891892 a.u.
M06-2X/BS2 SCF energy in solution:
-1647.305379 a.u.
M06-2X/BS2 Free energy in solution:
-1646.959322 a.u.

C	-1.044505	-0.237743	0.158047
O	-0.204772	-0.603754	-0.911776
O	-0.148497	0.416902	0.983241
H	-1.860595	-0.584075	-2.115666
H	-1.491609	-1.120425	0.631293
Si	1.226389	-0.001091	-0.122772
N	-2.138116	0.596360	-0.341334
C	-1.698769	1.909902	-0.790758
H	-0.806702	1.783962	-1.407620
H	-2.479329	2.362441	-1.406994
H	-1.452651	2.589557	0.034724
C	1.953571	1.758950	-0.185162
C	2.975864	2.143337	-1.070233
C	1.509040	2.727486	0.732939
C	3.520589	3.427238	-1.046773
H	3.346773	1.424934	-1.793036
C	2.052237	4.010978	0.767942
H	0.714949	2.465648	1.425240
C	3.060736	4.366395	-0.126070
H	4.305767	3.694404	-1.748538
H	1.686071	4.735217	1.490243
H	3.483985	5.366533	-0.105635
H	-2.997792	-2.725787	-3.554658
C	-2.591944	-2.232526	-2.659514
O	-2.248012	-0.983794	-2.926405
O	-2.486163	-2.777818	-1.585228
C	-3.369270	0.530147	0.345408
C	-4.055227	1.682804	0.754291
C	-3.967678	-0.721933	0.580252
C	-5.290468	1.582150	1.394307
H	-3.628709	2.664014	0.581346
C	-5.189949	-0.809680	1.234237
H	-3.482456	-1.619584	0.210541
C	-5.863824	0.340706	1.648389
H	-5.800963	2.490672	1.700344
H	-5.630893	-1.787958	1.402631

H	-6.823253	0.267473	2.149966
C	2.130794	-1.168125	1.079479
C	1.555048	-1.521861	2.311171
C	3.402804	-1.696148	0.794270
C	2.204328	-2.370641	3.207553
H	0.579889	-1.117552	2.565153
C	4.065337	-2.535072	1.688947
H	3.889941	-1.446083	-0.144640
C	3.464098	-2.879271	2.898776
H	1.728246	-2.632483	4.148294
H	5.049301	-2.922808	1.440999
H	3.974831	-3.537657	3.595456
O	2.184773	-0.499324	-1.578698
C	2.088779	-1.685590	-2.126261
H	1.365942	-2.367221	-1.641751
O	2.734806	-2.036150	-3.093252

IM10⁻

M06-2X/BS1 SCF energy in solution :
-1646.899184 a.u.
M06-2X/BS2 SCF energy in solution:
-1647.312429 a.u.
M06-2X/BS2 Free energy in solution:
-1646.964309 a.u.

C	0.935446	0.257686	-1.103386
O	0.590726	-0.241382	0.197666
O	-0.287615	0.736559	-1.498807
H	1.484795	-0.643405	1.422753
H	1.292148	-0.576060	-1.724458
Si	-1.115097	0.082250	-0.015757
N	1.974598	1.251240	-1.075595
C	1.617197	2.596183	-0.646460
H	0.530866	2.676443	-0.638716
H	2.001898	2.816418	0.357055
H	2.004913	3.350371	-1.338446
C	-2.066296	1.677306	0.384006
C	-2.704648	1.907604	1.614661
C	-2.216875	2.662190	-0.609146
C	-3.444485	3.066671	1.847632
H	-2.616419	1.168142	2.403133
C	-2.962130	3.819023	-0.387426
H	-1.730363	2.519731	-1.569609
C	-3.577389	4.026061	0.845931
H	-3.919711	3.219682	2.812453
H	-3.059834	4.560379	-1.175378
H	-4.154830	4.928342	1.025227
H	2.856315	-2.460858	3.147765
C	2.346256	-2.157596	2.220253
O	1.970375	-0.897515	2.272524
O	2.171650	-2.914199	1.288306
C	3.282049	0.815629	-0.847106
C	4.344210	1.739155	-0.870514
C	3.587043	-0.534237	-0.580901
C	5.655070	1.322970	-0.665047
H	4.142552	2.787571	-1.060491
C	4.905131	-0.934924	-0.382079
H	2.802043	-1.275708	-0.482928
C	5.952622	-0.017599	-0.425495
H	6.451817	2.060755	-0.694562
H	5.104916	-1.981777	-0.170446
H	6.976612	-0.336964	-0.262509
C	-2.151273	-1.235308	-0.909919
C	-1.934135	-1.536937	-2.264098
C	-3.180498	-1.933704	-0.253864
C	-2.691647	-2.501090	-2.929000
H	-1.158873	-0.999524	-2.802045
C	-3.953295	-2.887163	-0.914020
H	-3.386972	-1.726917	0.793201
C	-3.705486	-3.178441	-2.254786
H	-2.493179	-2.721019	-3.974138
H	-4.745493	-3.406200	-0.382180
H	-4.300449	-3.926620	-2.770375
O	-1.389108	-0.578522	1.645390
C	-0.940900	-1.754750	2.020535
H	-0.376696	-2.308718	1.247392
O	-1.117613	-2.223267	3.125977

TS11⁻

M06-2X/BS1 SCF energy in solution :
-1646.876345 a.u.
M06-2X/BS2 SCF energy in solution:
-1647.293483 a.u.
M06-2X/BS2 Free energy in solution:
-1646.953604 a.u.

C	1.016805	0.447805	-1.217165
O	0.495556	-0.237767	0.499204

O	-0.177850	0.881969	-1.424602
H	0.985042	-0.625418	1.514891
H	1.316226	-0.543156	-1.566412
Si	-1.103319	0.027884	0.117481
N	2.050619	1.301399	-1.054612
C	1.793798	2.714519	-0.791369
H	0.716303	2.857945	-0.724321
H	2.265968	3.008612	0.149343
H	2.184748	3.337675	-1.599673
C	-2.005150	1.662521	0.434226
C	-2.312709	2.097365	1.732526
C	-2.406453	2.482525	-0.633655
C	-2.976122	3.303825	1.958398
H	-2.032382	1.479347	2.580198
C	-3.088619	3.678245	-0.418586
H	-2.173192	2.181007	-1.651240
C	-3.370161	4.095596	0.882072
H	-3.191103	3.621675	2.974658
H	-3.395398	4.287991	-1.263617
H	-3.894190	5.031236	1.054112
H	2.417956	-2.535884	3.409092
C	1.987120	-2.169913	2.454027
O	1.485423	-0.987887	2.548261
O	2.024400	-2.881920	1.454274
C	3.347821	0.779962	-0.815386
C	4.469703	1.574383	-1.073920
C	3.513382	-0.508500	-0.289677
C	5.745445	1.075217	-0.828066
H	4.346134	2.576424	-1.471222
C	4.796781	-0.995520	-0.056760
H	2.657703	-1.122654	-0.027517
C	5.917895	-0.212952	-0.324235
H	6.608840	1.699507	-1.036163
H	4.909239	-1.992902	0.357968
H	6.914686	-0.596887	-0.133014
C	-2.014239	-1.180990	-1.041746
C	-1.623797	-1.407204	-2.372807
C	-3.135566	-1.895723	-0.581228
C	-2.298557	-2.310562	-3.193289
H	-0.781500	-0.858074	-2.779862
C	-3.828174	-2.786888	-1.399658
H	-3.482161	-1.751884	0.437993
C	-3.406115	-3.003457	-2.709517
H	-1.962244	-2.468344	-4.214098
H	-4.694615	-3.315067	-1.012065
H	-3.937242	-3.703108	-3.348189
O	-1.718050	-0.705213	1.645794
C	-1.318457	-1.894080	2.042909
H	-0.573830	-2.381185	1.386367
O	-1.727060	-2.439691	3.046788

1b

M06-2X/BS1 SCF energy in solution :
-440.078430 a.u.
M06-2X/BS2 SCF energy in solution:
-440.198314 a.u.
M06-2X/BS2 Free energy in solution:
-440.074776 a.u.

C	-2.330141	1.047942	-0.322316
C	-0.944517	1.167943	-0.264350
C	-0.159275	0.054037	0.050865
C	-0.777575	-1.171876	0.325263
C	-2.162282	-1.284097	0.243334
C	-2.946080	-0.177652	-0.078350
H	-2.928463	1.919957	-0.567249
H	-0.472986	2.122613	-0.472743
H	-0.181048	-2.026594	0.626596
H	-2.630247	-2.240171	0.456104
H	-4.026232	-0.267568	-0.127481
N	1.250143	0.180287	0.113870
C	1.852294	1.446657	0.513200
H	2.856347	1.241176	0.881924
H	1.921707	2.141979	-0.329489
H	1.253675	1.898909	1.305062
C	2.071149	-0.813346	-0.334271
H	1.525588	-1.689193	-0.716740
O	3.288651	-0.747594	-0.331666

IM11⁻

M06-2X/BS1 SCF energy in solution :
-1206.816315 a.u.
M06-2X/BS2 SCF energy in solution:
-1207.122147 a.u.
M06-2X/BS2 Free energy in solution:
-1206.924555 a.u.

O	0.247829	-1.317635	-1.634473

H	1.259182	-1.454564	-1.858011
Si	-0.153511	-0.587397	-0.252784
C	0.730157	1.041539	0.023208
C	0.344702	1.915913	1.050197
C	1.762247	1.442491	-0.837023
C	0.977243	3.146103	1.223557
H	-0.460361	1.636082	1.728325
C	2.392018	2.674940	-0.674624
H	2.080330	0.773863	-1.633998
C	2.001672	3.526984	0.358048
H	0.670222	3.807105	2.028599
H	3.189776	2.970918	-1.349607
H	2.494294	4.486129	0.487652
H	4.362869	-1.788404	-1.070087
C	3.261603	-1.615721	-1.025362
O	2.674570	-1.626150	-2.150163
O	2.748975	-1.436090	0.089141
C	-2.003244	-0.294266	-0.284734
C	-2.656193	-0.025706	-1.497352
C	-2.767853	-0.275573	0.891462
C	-4.021149	0.254216	-1.535854
H	-2.089898	-0.044361	-2.425664
C	-4.133000	0.005490	0.861367
H	-2.292020	-0.493519	1.844929
C	-4.761156	0.271946	-0.354223
H	-4.508793	0.455942	-2.485087
H	-4.706970	0.012701	1.783321
H	-5.824684	0.489956	-0.381081
O	0.003282	-1.556199	1.141424
C	0.940426	-1.452339	2.086947
H	1.539383	-0.533943	2.037014
O	1.079220	-2.287733	2.943944

HO[Si]OCHO

M06-2X/BS1 SCF energy in solution :
-1017.577579 a.u.
M06-2X/BS2 SCF energy in solution:
-1017.808088 a.u.
M06-2X/BS2 Free energy in solution:
-1017.631104 a.u.

Si	0.027469	0.612747	0.618018
C	-1.415253	-0.455193	0.151777
C	-2.591104	-0.457877	0.917839
C	-1.357551	-1.263818	-0.993621
C	-3.680255	-1.243032	0.546780
H	-2.652584	0.155349	1.813171
C	-2.445530	-2.049678	-1.366753
H	-0.452225	-1.288287	-1.596544
C	-3.607094	-2.038446	-0.595940
H	-4.583725	-1.236977	1.148539
H	-2.386794	-2.672148	-2.254072
H	-4.455111	-2.652109	-0.884331
C	1.671488	-0.138690	0.204822
C	2.713927	0.615957	-0.353406
C	1.895435	-1.498359	0.473739
C	3.947539	0.030029	-0.628996
H	2.558261	1.667296	-0.579539
C	3.127625	-2.086132	0.199115
H	1.097604	-2.108458	0.892654
C	4.154449	-1.320253	-0.351702
H	4.745534	0.624944	-1.062306
H	3.286471	-3.138962	0.410289
H	5.115010	-1.777688	-0.568388
O	0.028031	2.059377	-0.278389
C	-1.063401	2.765238	-0.608606
H	-2.018346	2.286058	-0.337858
O	-0.993492	3.827228	-1.164136
O	-0.189740	0.977136	2.211264
H	0.501292	1.494128	2.645924

H_2O

M06-2X/BS1 SCF energy in solution :
-76.391032 a.u.
M06-2X/BS2 SCF energy in solution:
-76.429719 a.u.
M06-2X/BS2 Free energy in solution:
-76.425739 a.u.

O	0.000000	0.000000	0.119090
H	0.000000	0.758321	-0.476361
H	0.000000	-0.758321	-0.476361

TS12

M06-2X/BS1 SCF energy in solution :
-1534.036399 a.u.
M06-2X/BS2 SCF energy in solution:
-1534.410517 a.u.

M06-2X/BS2 Free energy in solution:
-1534.067102 a.u.

Si	-0.952351	0.100943	0.495845
C	-1.822468	1.691766	0.088751
C	-2.232003	2.559800	1.112946
C	-2.118067	2.036303	-1.241522
C	-2.914902	3.738478	0.818386
H	-2.015612	2.313565	2.148563
C	-2.797526	3.215978	-1.535371
H	-1.799092	1.382189	-2.049286
C	-3.197840	4.066853	-0.505751
H	-3.225676	4.399109	1.621878
H	-3.016413	3.471541	-2.567598
H	-3.730340	4.984809	-0.735761
C	-1.908243	-1.429219	0.058253
C	-1.304927	-2.545429	-0.538242
C	-3.273655	-1.488962	0.379589
C	-2.050231	-3.693783	-0.801981
H	-0.254518	-2.514131	-0.816661
C	-4.018136	-2.636057	0.116910
H	-3.764260	-0.630866	0.835978
C	-3.404116	-3.740200	-0.473734
H	-1.574705	-4.552310	-1.266705
H	-5.073517	-2.669128	0.369158
H	-3.982614	-4.635518	-0.680858
O	0.144461	0.007541	-2.261130
O	-0.803589	0.150239	2.188091
C	0.937103	0.515105	-1.311592
H	0.958632	1.616022	-1.305309
C	-0.434147	-0.887410	2.951205
H	-0.264872	-1.827949	2.401250
O	0.627432	0.043811	-0.017085
O	-0.305890	-0.790434	4.141234
C	4.572933	1.393336	1.157691
C	3.745434	1.353713	0.037690
C	3.255097	0.126177	-0.399954
C	3.571409	-1.052247	0.269396
C	4.400515	-1.003209	1.386849
C	4.902901	0.218606	1.831452
H	4.958187	2.346768	1.504315
H	3.484026	2.269609	-0.482933
H	3.167039	-1.995140	-0.087251
H	4.652003	-1.920295	1.909671
H	5.549787	0.255669	2.702000
N	2.382696	0.042249	-1.556986
H	2.148801	-1.063380	-1.886576
C	2.918418	0.712827	-2.764903
H	3.910837	0.313763	-2.974543
H	2.244376	0.494823	-3.593468
H	2.973090	1.792960	-2.616069
O	1.356013	-2.040861	-2.375811
H	1.623070	-2.317840	-3.261641
H	0.573112	-1.100139	-2.429874

IM12

M06-2X/BS1 SCF energy in solution :
-1457.651874 a.u.
M06-2X/BS2 SCF energy in solution:
-1457.996682 a.u.
M06-2X/BS2 Free energy in solution:
-1457.669562 a.u.

Si	-0.865566	-0.150343	0.366550
C	-1.264177	1.662802	0.326059
C	-0.831260	2.540554	1.330596
C	-2.015090	2.181946	-0.742277
C	-1.133854	3.899583	1.267767
H	-0.258138	2.169458	2.177083
C	-2.317971	3.539083	-0.806515
H	-2.366543	1.522192	-1.532763
C	-1.876231	4.398270	0.199491
H	-0.793498	4.566945	2.053272
H	-2.899134	3.926751	-1.637296
H	-2.113731	5.456592	0.151424
C	-2.341354	-1.242039	0.134317
C	-2.222732	-2.464393	-0.544134
C	-3.598368	-0.864718	0.630111
C	-3.328702	-3.294436	-0.710618
H	-1.259669	-2.766135	-0.948025
C	-4.705540	-1.693227	0.464334
H	-3.717570	0.086204	1.144926
C	-4.569478	-2.908670	-0.205189
H	-3.224754	-4.237988	-1.237242
H	-5.673307	-1.390040	0.851325
H	-5.432816	-3.553645	-0.337657
O	0.063290	0.299415	-2.835787
O	-0.204772	-0.510437	1.886029

C	0.862462	0.245979	-1.707608
H	0.932755	1.282810	-1.337793
C	1.054855	-0.228764	2.266324
H	1.685378	0.216021	1.478225
O	0.294211	-0.583599	-0.733092
O	1.442005	-0.446678	3.381328
C	4.532970	0.191376	0.843813
C	3.809051	0.452753	-0.321053
C	2.971311	-0.526911	-0.860827
C	2.873361	-1.769429	-0.225878
C	3.589852	-2.023871	0.938649
C	4.422224	-1.042247	1.479254
H	5.180497	0.959846	1.255015
H	3.897134	1.421681	-0.802850
H	2.221400	-2.521751	-0.657367
H	3.501249	-2.990590	1.424886
H	4.982044	-1.241040	2.387653
N	2.175722	-0.293592	-2.033563
C	2.827498	0.538163	-3.048271
H	3.846247	0.177441	-3.199136
H	2.282386	0.451214	-3.988746
H	2.866442	1.603679	-2.776081
H	0.040661	-0.600120	-3.203515

TS13

M06-2X/BS1 SCF energy in solution :
-1534.051803 a.u.
M06-2X/BS2 SCF energy in solution:
-1534.426329 a.u.
M06-2X/BS2 Free energy in solution:
-1534.076509 a.u.

Si	0.815621	0.122867	0.211082
C	0.056987	1.832599	0.239312
C	-0.363805	2.489232	-0.926958
C	-0.118297	2.487401	1.467147
C	-0.955252	3.749501	-0.867866
H	-0.252316	1.998638	-1.891111
C	-0.712445	3.747686	1.534430
H	0.198151	1.996907	2.385469
C	-1.135195	4.377737	0.364961
H	-1.283470	4.239448	-1.779871
H	-0.848138	4.235651	2.495031
H	-1.602951	5.356532	0.412994
C	2.652042	0.185748	-0.155765
C	3.584526	-0.048867	0.864075
C	3.134543	0.489918	-1.437776
C	4.955393	0.018162	0.615418
H	3.233978	-0.293300	1.864613
C	4.501912	0.557379	-1.693361
H	2.433713	0.667718	-2.251614
C	5.414655	0.322534	-0.664349
H	5.663298	-0.168400	1.417498
H	4.857628	0.790317	-2.692618
H	6.481147	0.374469	-0.861950
O	-0.841519	-2.785533	0.786297
O	0.088896	-0.505719	-1.228691
C	-1.115283	-1.874181	1.679925
H	-0.804769	-2.025030	2.709421
C	0.395117	-1.671353	-1.768119
H	-0.205528	-1.873799	-2.666443
O	0.504305	-0.734278	1.529484
O	1.235491	-2.444151	-1.359944
N	-2.195057	-1.102112	1.516809
H	1.491623	-2.175356	1.578613
O	1.616156	-3.128630	1.339550
H	1.953568	-3.071281	0.430502
H	0.099619	-3.128514	0.983083
C	-2.498349	-0.172432	2.604703
H	-3.566872	0.044854	2.598153
H	-2.235722	-0.642915	3.552739
H	-1.932175	0.757474	2.498492
C	-2.763552	-0.846137	0.228516
C	-3.091700	-1.899965	-0.629265
C	-3.019901	0.472014	-0.154219
C	-3.634039	-1.624434	-1.880677
H	-2.926995	-2.923187	-0.317077
C	-3.570389	0.734659	-1.406579
H	-2.775510	1.295584	0.508733
C	-3.870905	-0.308885	-2.277606
H	-3.883074	-2.447168	-2.543521
H	-3.755160	1.763727	-1.699080
H	-4.296361	-0.101124	-3.254112

TS14

M06-2X/BS1 SCF energy in solution :
-1420.754723 a.u.
M06-2X/BS2 SCF energy in solution:

-1421.092992 a.u.

M06-2X/BS2 Free energy in solution:

-1420.754333 a.u.

Si	0.766094	0.063483	0.262078
C	2.381520	-0.868131	0.341259
C	3.181470	-0.807403	1.492409
C	2.844481	-1.616052	-0.750995
C	4.405559	-1.470183	1.551346
H	2.843722	-0.239709	2.357011
C	4.069134	-2.279971	-0.698248
H	2.238614	-1.683034	-1.652066
C	4.850206	-2.207100	0.454033
H	5.010857	-1.415568	2.451141
H	4.412565	-2.855898	-1.552353
H	5.803474	-2.725145	0.497964
C	1.036861	1.866034	-0.119924
C	1.942494	2.266013	-1.113866
C	0.318144	2.856319	0.565392
C	2.115926	3.612731	-1.425056
H	2.523838	1.518069	-1.650321
C	0.491540	4.205726	0.261285
H	-0.383597	2.564108	1.342530
C	1.388855	4.583576	-0.736389
H	2.818247	3.906501	-2.199196
H	-0.071075	4.961459	0.801333
H	1.524467	5.634099	-0.975571
O	-0.102367	-0.546971	-1.042102
C	-0.800048	-1.736283	-0.930713
H	-0.561131	-2.373193	-1.798118
O	-0.644450	-2.344753	0.262260
N	-2.314289	-1.438005	-1.129592
H	-2.706973	-2.504363	-0.742185
O	-2.634180	-3.616139	-0.018423
H	-2.544718	-4.407505	-0.564876
H	-1.522756	-3.136455	0.256069
C	-2.776651	-0.402130	-0.222622
C	-2.715412	0.944161	-0.580255
C	-3.239011	-0.780848	1.037858
C	-3.134142	1.913536	0.328228
H	-2.344857	1.239638	-1.555788
C	-3.648143	0.197449	1.940970
H	-3.287700	-1.833091	1.298795
C	-3.597360	1.544577	1.589936
H	-3.086831	2.961556	0.048832
H	-4.011462	-0.098320	2.920022
H	-3.920980	2.304193	2.294378
C	-2.619000	-1.202056	-2.554244
H	-2.451621	-2.134471	-3.096028
H	-3.663142	-0.906051	-2.655281
H	-1.970566	-0.425537	-2.965587
O	-0.068847	0.001056	1.681161
H	-0.553463	-0.839338	1.731757

IM13

M06-2X/BS1 SCF energy in solution :

-1344.362529 a.u.

M06-2X/BS2 SCF energy in solution:

-1344.671291 a.u.

M06-2X/BS2 Free energy in solution:

-1344.352118 a.u.

Si	0.928392	0.022064	-0.879074
C	1.896908	1.528636	-0.371857
C	1.914508	2.661435	-1.200166
C	2.617500	1.571536	0.831632
C	2.625887	3.803303	-0.836803
H	1.372240	2.646617	-2.142012
C	3.332057	2.710328	1.197028
H	2.617529	0.706888	1.490449
C	3.334806	3.827805	0.362942
H	2.629499	4.671294	-1.489041
H	3.886372	2.727024	2.130407
H	3.891565	4.715866	0.646753
C	1.755684	-1.575342	-0.385422
C	0.999724	-2.673960	0.051750
C	3.148175	-1.719870	-0.475551
C	1.613350	-3.880094	0.384551
H	-0.082370	-2.584986	0.135073
C	3.766833	-2.923282	-0.142596
H	3.760025	-0.881161	-0.801969
C	2.998438	-4.004609	0.286709
H	1.013671	-4.720256	0.721165
H	4.846081	-3.017464	-0.214282
H	3.479339	-4.942533	0.547396
O	0.069016	-0.107504	1.921114
C	-0.915580	0.346445	1.044711
H	-0.912338	1.444698	1.144947

O	-0.632066	-0.002599	-0.273594
C	-5.282045	0.978838	-0.354430
C	-4.264071	0.885229	0.595290
C	-3.247431	-0.064443	0.451262
C	-3.276054	-0.922912	-0.656075
C	-4.284378	-0.816467	-1.606617
C	-5.295436	0.134822	-1.460297
H	-6.064069	1.720962	-0.225201
H	-4.268181	1.556696	1.447346
H	-2.499917	-1.673774	-0.753985
H	-4.290483	-1.490405	-2.458035
H	-6.088216	0.209614	-2.197855
N	-2.205510	-0.220029	1.420899
C	-2.563130	0.206271	2.774949
H	-3.539905	-0.206567	3.031973
H	-1.824800	-0.179246	3.478272
H	-2.600869	1.300226	2.887564
H	-0.017865	-1.074936	1.968283
O	0.766679	0.158678	-2.519086
H	0.250287	-0.527867	-2.960035

TS15

M06-2X/BS1 SCF energy in solution :
-1420.751633 a.u.
M06-2X/BS2 SCF energy in solution:
-1421.093568 a.u.
M06-2X/BS2 Free energy in solution:
-1420.752315 a.u.

Si	-0.831958	-0.077840	-0.002333
C	-0.212373	1.667319	-0.329535
C	0.210276	2.504126	0.714389
C	-0.137203	2.161978	-1.640014
C	0.699304	3.784618	0.460991
H	0.178247	2.138422	1.738715
C	0.353145	3.441033	-1.903746
H	-0.453016	1.528479	-2.466540
C	0.774234	4.253073	-0.851149
H	1.028324	4.414931	1.282202
H	0.410040	3.803363	-2.926056
H	1.159982	5.248135	-1.052240
C	-2.677151	-0.080731	0.360816
C	-3.592908	-0.502602	-0.614449
C	-3.185886	0.342996	1.598599
C	-4.965637	-0.505230	-0.366417
H	-3.224074	-0.840260	-1.580955
C	-4.555519	0.344411	1.854552
H	-2.500351	0.668156	2.378249
C	-5.448116	-0.080320	0.870001
H	-5.657145	-0.839223	-1.134442
H	-4.928417	0.673080	2.820287
H	-6.515941	-0.081420	1.067730
O	0.906589	-2.947711	-0.277365
C	1.139469	-2.176999	-1.300937
H	0.795140	-2.471925	-2.288125
O	-0.530801	-1.034190	-1.271531
N	2.190310	-1.351142	-1.280070
H	-1.345751	-2.367824	-1.108707
O	-1.490924	-3.345063	-0.865636
H	-2.126401	-3.352268	-0.137254
H	-0.044946	-3.319961	-0.402326
C	2.406510	-0.544457	-2.481541
H	3.453024	-0.242248	-2.521930
H	2.175742	-1.149896	-3.358366
H	1.762925	0.340392	-2.477413
C	2.762531	-0.898689	-0.049704
C	3.177934	-1.816320	0.921415
C	2.920249	0.472617	0.169501
C	3.712534	-1.352832	2.119861
H	3.081944	-2.878515	0.735008
C	3.463089	0.922721	1.371308
H	2.605348	1.188596	-0.582242
C	3.854134	0.015015	2.351654
H	4.029208	-2.068725	2.871434
H	3.569356	1.990138	1.537605
H	4.272242	0.369045	3.288236
O	-0.181826	-0.572565	1.454392
H	0.773749	-0.716859	1.457825

[Si](OH)$_2$

M06-2X/BS1 SCF energy in solution :
-904.289538 a.u.
M06-2X/BS2 SCF energy in solution:
-904.485462 a.u.
M06-2X/BS2 Free energy in solution:
-904.313887 a.u.

Si	0.000000	0.000000	1.197767

C	0.000000	1.538115	0.145372
C	-0.761404	2.664920	0.488749
C	0.792533	1.598714	-1.011397
C	-0.729514	3.816982	-0.295121
H	-1.390327	2.637185	1.374800
C	0.828870	2.747974	-1.797912
H	1.382128	0.733770	-1.309355
C	0.067312	3.859179	-1.438196
H	-1.326542	4.680229	-0.016925
H	1.445805	2.776765	-2.690850
H	0.091930	4.755687	-2.050337
C	0.000000	-1.538115	0.145372
C	0.761404	-2.664920	0.488749
C	-0.792533	-1.598714	-1.011397
C	0.729514	-3.816982	-0.295121
H	1.390327	-2.637185	1.374800
C	-0.828870	-2.747974	-1.797912
H	-1.382128	-0.733770	-1.309355
C	-0.067312	-3.859179	-1.438196
H	1.326542	-4.680229	-0.016925
H	-1.445805	-2.776765	-2.690850
H	-0.091930	-4.755687	-2.050337
O	-1.362601	0.156746	2.129335
H	-1.556450	-0.578523	2.724644
O	1.362601	-0.156746	2.129335
H	1.556450	0.578523	2.724644

TS16

M06-2X/BS1 SCF energy in solution :
-1975.158670 a.u.
M06-2X/BS2 SCF energy in solution:
-1975.577694 a.u.
M06-2X/BS2 Free energy in solution:
-1975.109282 a.u.

C	-3.966441	1.282859	-0.982538
C	-3.174722	2.368466	-0.935664
H	-4.902284	1.179225	-1.513301
H	-3.339607	3.317224	-1.426842
N	-3.487348	0.244541	-0.177558
N	-2.075189	2.186551	-0.089692
P	-1.935414	0.570807	0.418315
H	-1.063379	0.010607	-0.860938
C	-0.947860	3.152753	-0.081604
C	-4.141818	-1.087861	-0.165019
C	0.130981	2.644003	0.872046
H	0.563159	1.706003	0.503358
H	0.939714	3.377670	0.930168
H	-0.264532	2.481616	1.880237
C	-0.364161	3.245249	-1.495396
H	-1.097035	3.646022	-2.202021
H	0.508218	3.906024	-1.496885
H	-0.051090	2.252048	-1.837271
C	-1.461484	4.512791	0.390908
H	-2.238365	4.901415	-0.273588
H	-1.872245	4.436607	1.401992
H	-0.637581	5.232319	0.400467
C	-3.362683	-2.013972	0.765716
H	-3.877661	-2.976884	0.824324
H	-2.352912	-2.193216	0.376878
H	-3.291665	-1.601566	1.777839
C	-4.117832	-1.667992	-1.582475
H	-4.599022	-2.650836	-1.586665
H	-4.654184	-1.024468	-2.286240
H	-3.082246	-1.785880	-1.919619
C	-5.575586	-0.922743	0.339899
H	-5.576758	-0.497741	1.348153
H	-6.160276	-0.269906	-0.314823
H	-6.069648	-1.897996	0.370933
Si	1.917522	-0.927717	-0.357983
C	3.357419	0.237307	-0.211359
C	4.484699	-0.099730	0.554798
C	3.318813	1.496847	-0.827045
C	5.543740	0.793277	0.696259
H	4.538471	-1.067049	1.050833
C	4.375460	2.394298	-0.684061
H	2.452498	1.776909	-1.421372
C	5.488313	2.041818	0.076984
H	6.410063	0.518650	1.290108
H	4.330060	3.366377	-1.165829
H	6.311834	2.740484	0.189577
C	1.089125	-1.210544	1.282057
C	0.211074	-2.285558	1.490223
C	1.307419	-0.312012	2.338174
C	-0.446421	-2.442399	2.708168
H	0.025380	-2.986421	0.682078
C	0.643065	-0.460302	3.554094
H	1.998121	0.519103	2.210903

C	-0.240199	-1.522837	3.736587
H	-1.129758	-3.274697	2.851184
H	0.815542	0.250403	4.356550
H	-0.760148	-1.640126	4.682806
O	0.945249	-0.231382	-1.518107
C	-0.320666	-0.799527	-1.770040
H	-0.743011	-0.323439	-2.670890
O	-0.499422	-2.003300	-1.503267
O	2.453456	-2.442298	-0.893032
C	2.259985	-2.953843	-2.118193
H	1.835161	-2.247757	-2.845044
O	2.552905	-4.087336	-2.387392

TS16'

M06-2X/BS1 SCF energy in solution :
-2073.858630 a.u.
M06-2X/BS2 SCF energy in solution:
-2074.331037 a.u.
M06-2X/BS2 Free energy in solution:
-2073.939171 a.u.

H	4.491192	-0.741600	3.547123
C	4.000284	-1.093671	2.619270
O	3.920517	-2.283360	2.367267
O	3.568090	-0.106874	1.888838
Si	2.332964	-0.312424	0.516581
H	1.621087	-1.372050	1.290850
H	1.197255	-0.553677	-0.622154
C	3.653502	-0.665775	-0.825387
C	3.347179	-0.365246	-2.163292
C	4.937201	-1.169945	-0.563779
C	4.273211	-0.543151	-3.190164
H	2.359303	0.023753	-2.404852
C	5.864847	-1.372104	-1.585987
H	5.217542	-1.427134	0.452579
C	5.537731	-1.053919	-2.902886
H	4.007316	-0.289003	-4.212349
H	6.846780	-1.774777	-1.353269
H	6.261644	-1.203895	-3.698524
C	1.806069	1.524507	0.652587
C	0.828076	2.026609	-0.222931
C	2.334244	2.423814	1.595330
C	0.388948	3.349162	-0.160704
H	0.388206	1.362621	-0.963569
C	1.906266	3.749855	1.662383
H	3.091020	2.076250	2.290049
C	0.928799	4.217631	0.785633
H	-0.382167	3.695046	-0.845648
H	2.333873	4.418442	2.404549
H	0.589678	5.248021	0.841646
H	0.669202	-1.787137	-2.056559
C	0.058888	-1.642863	-1.156254
O	-0.092984	-2.498319	-0.290050
O	-0.921978	-0.696015	-1.391039
Si	-2.027548	-0.340298	-0.191120
C	-3.428775	-1.568357	-0.162821
C	-3.266676	-2.899950	-0.577257
C	-4.697295	-1.152478	0.272023
C	-4.339701	-3.788005	-0.551688
H	-2.290846	-3.242790	-0.909208
C	-5.770126	-2.040683	0.302915
H	-4.855363	-0.121877	0.583347
C	-5.590966	-3.359649	-0.110265
H	-4.200212	-4.814937	-0.875359
H	-6.744267	-1.703355	0.643117
H	-6.426422	-4.052957	-0.091005
C	-2.588840	1.396312	-0.524686
C	-3.010648	2.237375	0.515535
C	-2.588783	1.897722	-1.835050
C	-3.423116	3.542577	0.254543
H	-3.003784	1.876842	1.541478
C	-3.002569	3.201952	-2.099514
H	-2.249619	1.269481	-2.655231
C	-3.419718	4.024967	-1.053637
H	-3.741852	4.183912	1.070434
H	-2.995197	3.577621	-3.118081
H	-3.738633	5.042565	-1.257940
O	-1.277595	-0.264337	1.319975
C	-1.106023	-1.285738	2.178110
H	-1.515407	-2.245338	1.833630
O	-0.570824	-1.132545	3.242052

IM14-

M06-2X/BS1 SCF energy in solution :
-1131.585047 a.u.
M06-2X/BS2 SCF energy in solution:
-1131.854338 a.u.
M06-2X/BS2 Free energy in solution:

-1131.654025 a.u.

H	-0.935066	0.578163	3.542945
Si	0.074496	0.408156	0.677945
C	1.730316	-0.169779	-0.067731
C	2.102754	0.106576	-1.395709
C	2.620282	-0.944666	0.694425
C	3.296582	-0.368502	-1.935736
H	1.448599	0.713535	-2.013828
C	3.823680	-1.414244	0.167234
H	2.353584	-1.179835	1.720026
C	4.164237	-1.129827	-1.153170
H	3.553447	-0.141233	-2.966708
H	4.493695	-2.004907	0.785944
H	5.097668	-1.497224	-1.569927
C	-1.445947	-0.513355	-0.019675
C	-2.178126	-1.398104	0.788896
C	-1.872773	-0.367227	-1.352013
C	-3.288285	-2.089870	0.303693
H	-1.859304	-1.543396	1.816799
C	-2.969773	-1.066723	-1.852301
H	-1.336053	0.305860	-2.016016
C	-3.686151	-1.927678	-1.021675
H	-3.840194	-2.758947	0.958137
H	-3.269248	-0.937561	-2.888601
H	-4.546261	-2.468381	-1.405951
O	0.201723	-0.540404	2.204556
C	-0.005545	0.616922	2.948286
H	0.837359	0.843801	3.623247
O	-0.106443	1.590265	1.925932
O	-0.033409	1.756545	-0.553411
C	-1.038917	2.589628	-0.578361
H	-1.808754	2.418295	0.197966
O	-1.155175	3.495935	-1.381106

TS17

M06-2X/BS1 SCF energy in solution :
-1975.167793 a.u.
M06-2X/BS2 SCF energy in solution:
-1975.587795 a.u.
M06-2X/BS2 Free energy in solution:
-1975.113816 a.u.

C	1.670403	-1.964927	1.556219
C	2.613375	-1.023691	1.398823
H	1.348428	-2.420396	2.481640
H	3.207179	-0.554882	2.170823
N	1.082780	-2.327623	0.336385
N	2.766244	-0.658776	0.051871
P	1.592006	-1.353269	-0.946562
H	-0.496480	-0.684870	-3.033497
C	3.795869	0.322086	-0.371780
C	0.124095	-3.458360	0.244483
C	3.776620	0.458798	-1.893282
H	2.811278	0.836513	-2.248648
H	4.545916	1.175194	-2.192855
H	3.986659	-0.495579	-2.385657
C	3.492944	1.683426	0.260645
H	3.493543	1.623608	1.353697
H	4.253939	2.410502	-0.038735
H	2.514983	2.049545	-0.071514
C	5.170632	-0.182667	0.074066
H	5.233306	-0.267511	1.162495
H	5.375593	-1.164028	-0.364099
H	5.945279	0.516200	-0.254336
C	-0.410864	-3.554729	-1.183732
H	-1.131307	-4.375192	-1.237651
H	-0.929839	-2.633524	-1.475404
H	0.388434	-3.758531	-1.902867
C	-1.045434	-3.213464	1.201231
H	-1.765209	-4.033355	1.117200
H	-0.712394	-3.164751	2.241921
H	-1.550022	-2.275402	0.955289
C	0.855489	-4.751622	0.611667
H	1.690099	-4.925515	-0.074495
H	1.247926	-4.704117	1.632080
H	0.168946	-5.601213	0.550411
Si	-0.961249	0.709217	-0.213637
C	-0.813097	2.572158	-0.050513
C	-1.387233	3.195579	1.069104
C	-0.094030	3.368830	-0.952554
C	-1.266616	4.569527	1.270599
H	-1.937992	2.604957	1.799394
C	0.052165	4.738447	-0.741701
H	0.350002	2.913688	-1.833114
C	-0.540817	5.342300	0.366479
H	-1.730453	5.033354	2.135779
H	0.622262	5.336574	-1.446232

H	-0.436124	6.411340	0.525286
C	-2.647168	-0.118044	-0.254460
C	-3.376619	-0.197045	0.945245
C	-3.209162	-0.706527	-1.398188
C	-4.616318	-0.830953	0.999551
H	-2.961830	0.224179	1.856201
C	-4.435697	-1.365968	-1.341912
H	-2.688416	-0.637835	-2.346662
C	-5.145140	-1.425456	-0.144151
H	-5.162713	-0.869978	1.937036
H	-4.841805	-1.825955	-2.237803
H	-6.104760	-1.931896	-0.102546
O	-1.244293	1.134645	-2.439600
C	-0.297443	0.360590	-2.740871
H	0.646843	0.760332	-3.143616
O	0.319075	-0.042831	-1.051792
O	-0.591235	0.258151	1.420139
C	0.330406	0.825515	2.193671
H	1.078393	1.429629	1.654245
O	0.341002	0.701398	3.393244

CH_2O

M06-2X/BS1 SCF energy in solution :
-114.450935 a.u.
M06-2X/BS2 SCF energy in solution:
-114.492352 a.u.
M06-2X/BS2 Free energy in solution:
-114.486705 a.u.

C	-0.000011	0.530565	0.000000
H	0.937947	1.114778	0.000000
H	-0.937788	1.115108	0.000000
O	-0.000011	-0.676659	0.000000

IM15

M06-2X/BS1 SCF energy in solution :
-1860.739181 a.u.
M06-2X/BS2 SCF energy in solution:
-1861.121961 a.u.
M06-2X/BS2 Free energy in solution:
-1860.678555 a.u.

C	2.225556	-1.304441	1.339759
C	2.836769	-0.134940	1.096835
H	2.142443	-1.812335	2.290284
H	3.341487	0.497210	1.814441
N	1.661352	-1.855130	0.177361
N	2.775109	0.220775	-0.261395
P	1.683191	-0.763543	-1.131820
C	3.292997	1.525967	-0.728503
C	1.136641	-3.240196	0.167428
C	3.108257	1.634684	-2.242271
H	2.048559	1.625769	-2.519588
H	3.536108	2.579986	-2.586736
H	3.616610	0.817665	-2.763685
C	2.525308	2.659495	-0.038501
H	2.670121	2.629110	1.046289
H	2.875883	3.631921	-0.398763
H	1.454171	2.574987	-0.251732
C	4.785858	1.614753	-0.399849
H	4.965845	1.550399	0.677080
H	5.332155	0.804518	-0.892169
H	5.187676	2.570386	-0.749478
C	0.606954	-3.577633	-1.225254
H	0.201753	-4.593148	-1.214735
H	-0.201237	-2.897326	-1.518114
H	1.398891	-3.533945	-1.978902
C	-0.004209	-3.364813	1.182392
H	-0.367063	-4.397077	1.206898
H	0.324964	-3.097871	2.190971
H	-0.836426	-2.710121	0.907747
C	2.272475	-4.203052	0.526711
H	3.096047	-4.100628	-0.186719
H	2.656765	-4.003994	1.531702
H	1.913297	-5.236157	0.500156
Si	-1.035561	0.345122	0.066125
C	-1.517643	2.144793	0.009073
C	-2.193030	2.744482	1.083365
C	-1.245416	2.922391	-1.126539
C	-2.579418	4.081850	1.027876
H	-2.417596	2.166360	1.977938
C	-1.631044	4.260026	-1.186198
H	-0.716908	2.478859	-1.967245
C	-2.297761	4.840034	-0.107873
H	-3.095968	4.533198	1.869315
H	-1.409377	4.850172	-2.070122
H	-2.596288	5.883038	-0.151649
C	-2.421027	-0.826380	-0.337229

C	-3.353152	-1.225762	0.632906
C	-2.570355	-1.305592	-1.647157
C	-4.399083	-2.085183	0.304519
H	-3.252861	-0.875013	1.657337
C	-3.616282	-2.164769	-1.979251
H	-1.855588	-1.012465	-2.413063
C	-4.530415	-2.555117	-1.002114
H	-5.108949	-2.391985	1.066485
H	-3.716117	-2.531265	-2.996280
H	-5.344486	-3.226612	-1.257591
O	0.215015	0.124098	-0.967352
O	-0.669697	-0.014092	1.681860
C	0.117873	0.727097	2.478122
H	0.592799	1.588514	1.980695
O	0.262051	0.476695	3.644184

2a

M06-2X/BS1 SCF energy in solution :
-408.984510 a.u.
M06-2X/BS2 SCF energy in solution:
-409.085399 a.u.
M06-2X/BS2 Free energy in solution:
-408.847465 a.u.

C	-1.251302	1.221097	-0.456297
C	-1.274392	-0.262104	-0.040874
C	1.274390	-0.262109	-0.040873
C	1.251310	1.221084	-0.456319
C	0.000013	1.944127	0.041581
H	-1.270591	1.270501	-1.554424
H	-2.160622	1.717197	-0.095224
H	2.160642	1.717179	-0.095270
H	1.270575	1.270475	-1.554447
H	0.000026	1.993333	1.137484
H	0.000017	2.979912	-0.314151
N	-0.000002	-0.946466	-0.360059
H	-0.000001	-1.081344	-1.371197
C	-2.356651	-0.994910	-0.836105
H	-2.409200	-2.046472	-0.535742
H	-3.335529	-0.535900	-0.667469
H	-2.143643	-0.954575	-1.910533
C	-1.613834	-0.398147	1.449347
H	-2.663293	-0.132176	1.613363
H	-1.465200	-1.431767	1.776806
H	-1.011084	0.251948	2.086136
C	2.356659	-0.994930	-0.836081
H	3.335537	-0.535924	-0.667436
H	2.409197	-2.046489	-0.535703
H	2.143668	-0.954608	-1.910512
C	1.613810	-0.398127	1.449353
H	1.465195	-1.431746	1.776823
H	2.663260	-0.132128	1.613384
H	1.011031	0.251961	2.086123

TS18

M06-2X/BS1 SCF energy in solution :
-523.413533 a.u.
M06-2X/BS2 SCF energy in solution:
-523.553548 a.u.
M06-2X/BS2 Free energy in solution:
-523.288416 a.u.

C	1.240838	-1.473712	-0.780648
C	1.304190	-0.193364	0.065837
C	-1.304186	-0.193414	0.065839
C	-1.240779	-1.473755	-0.780656
C	0.000042	-2.315653	-0.512865
H	1.250500	-1.185675	-1.840166
H	2.156882	-2.043569	-0.591279
H	-2.156805	-2.043648	-0.591307
H	-1.250437	-1.185705	-1.840171
H	0.000046	-2.690986	0.516698
H	0.000061	-3.193879	-1.165591
N	-0.000018	0.549812	-0.085326
H	-0.000031	1.288519	-1.027100
C	2.419302	0.691670	-0.507539
H	2.611081	1.575652	0.104913
H	3.340324	0.102646	-0.542186
H	2.178442	1.018767	-1.522206
C	1.626154	-0.520901	1.528187
H	2.674163	-0.828894	1.593931
H	1.493155	0.352730	2.172411
H	1.019532	-1.336765	1.923273
C	-2.419339	0.691580	-0.507525
H	-3.340371	0.102565	-0.542038
H	-2.611052	1.575626	0.104855
H	-2.178559	1.018573	-1.522245
C	-1.626120	-0.520957	1.528196

H	-1.493350	0.352744	2.172371
H	-2.674059	-0.829192	1.593921
H	-1.019314	-1.336649	1.923352
C	-0.000028	1.988461	0.460256
H	0.888144	2.126141	1.099266
H	-0.888180	2.126120	1.099300
O	-0.000060	2.638307	-0.734651

TS18'

M06-2X/BS1 SCF energy in solution :
-599.848530 a.u.
M06-2X/BS2 SCF energy in solution:
-600.020679 a.u.
M06-2X/BS2 Free energy in solution:
-599.731379 a.u.

C	-1.734623	-0.860875	-0.978319
C	-0.781438	-1.166652	0.188498
C	-0.080126	1.351590	0.121166
C	-1.033555	1.528348	-1.068989
C	-2.226788	0.580434	-1.022983
H	-1.202638	-1.082798	-1.913501
H	-2.572674	-1.562462	-0.910543
H	-1.358160	2.574132	-1.083634
H	-0.468634	1.350556	-1.994500
H	-2.862751	0.799041	-0.158156
H	-2.848447	0.726701	-1.911354
N	0.323844	-0.117831	0.170718
H	0.833165	-0.296051	-0.773802
C	-0.136125	-2.532811	-0.093776
H	0.349401	-2.959974	0.786337
H	-0.928065	-3.223921	-0.396330
H	0.593321	-2.459173	-0.905585
C	-1.516013	-1.222551	1.528471
H	-2.080717	-2.158880	1.566520
H	-0.816115	-1.223727	2.369448
H	-2.224548	-0.405457	1.665508
C	1.193074	2.163003	-0.142164
H	0.901524	3.196426	-0.349276
H	1.868703	2.156588	0.715791
H	1.736558	1.770355	-1.004417
C	-0.732042	1.852545	1.413873
H	-0.194011	1.528786	2.307770
H	-0.709489	2.946160	1.395600
H	-1.774915	1.551985	1.510865
C	1.488207	-0.391637	1.163760
H	1.261512	-1.373497	1.603613
H	1.383486	0.352724	1.965695
O	2.682216	-0.320542	0.534445
O	1.994339	-0.749772	-1.709826
H	2.496950	-0.574941	-0.628720
H	2.293775	-0.053600	-2.306528

IM16

M06-2X/BS1 SCF energy in solution :
-523.463967 a.u.
M06-2X/BS2 SCF energy in solution:
-523.602275 a.u.
M06-2X/BS2 Free energy in solution:
-523.334126 a.u.

C	0.547401	-1.854597	-0.779710
C	1.091410	-0.683776	0.053288
C	-1.286028	0.317587	0.047584
C	-1.734401	-0.902519	-0.774526
C	-0.917269	-2.155513	-0.493450
H	0.651689	-1.599486	-1.842763
H	1.177886	-2.731121	-0.591945
H	-2.799458	-1.071205	-0.579538
H	-1.634615	-0.653572	-1.839525
H	-1.050572	-2.479760	0.545344
H	-1.264266	-2.979440	-1.125708
N	0.184471	0.485004	-0.083876
H	0.780116	2.401902	-1.330546
C	2.458801	-0.304539	-0.533577
H	2.973130	0.451775	0.065387
H	3.095700	-1.194256	-0.569181
H	2.344029	0.086994	-1.548108
C	1.299991	-1.141408	1.510279
H	2.118184	-1.867983	1.556032
H	1.567978	-0.301725	2.159146
H	0.410874	-1.618960	1.926991
C	-1.978234	1.541204	-0.575312
H	-3.052742	1.348027	-0.653634
H	-1.855283	2.451144	0.018257
H	-1.589955	1.725314	-1.582479
C	-1.766934	0.171951	1.503664
H	-1.353168	0.963683	2.136699

H	-2.858801	0.247356	1.546750
H	-1.484447	-0.789954	1.937184
C	0.713230	1.711696	0.457773
H	1.495327	1.532802	1.203364
H	-0.080825	2.272732	0.961660
O	1.318764	2.526273	-0.537791

TS19$^+$

M06-2X/BS1 SCF energy in solution :
-1367.055879 a.u.
M06-2X/BS2 SCF energy in solution:
-1367.340308 a.u.
M06-2X/BS2 Free energy in solution:
-1366.798341 a.u.

C	4.096292	1.129900	-1.201960
C	3.096329	1.296830	-0.050012
C	3.108181	-1.358802	0.108550
C	4.106992	-1.320367	-1.055924
C	4.994712	-0.087067	-1.054823
H	3.538751	1.039295	-2.142992
H	4.676579	2.056417	-1.257857
H	4.695403	-2.241777	-1.002906
H	3.548544	-1.346443	-2.000395
H	5.590257	-0.029875	-0.136613
H	5.699984	-0.133195	-1.889814
N	2.439344	-0.018762	0.286292
C	2.036083	2.300881	-0.513126
H	1.339581	2.592209	0.277980
H	2.545258	3.210278	-0.845116
H	1.472983	1.904191	-1.364028
C	3.805577	1.831192	1.200053
H	4.205064	2.829036	0.996180
H	3.108318	1.909121	2.040084
H	4.635058	1.186077	1.500295
C	2.052531	-2.415269	-0.233595
H	2.566208	-3.350364	-0.475049
H	1.367371	-2.621191	0.593218
H	1.470755	-2.103956	-1.105289
C	3.820523	-1.734372	1.413152
H	3.120447	-1.727507	2.254467
H	4.235591	-2.742958	1.325975
H	4.638396	-1.046252	1.641576
C	1.339716	0.017579	1.023820
H	1.068899	0.963502	1.477524
H	1.089895	-0.860080	1.608919
O	-0.161027	-0.142855	-0.002646
H	-0.041136	0.235500	-0.894835
C	-3.248497	-0.590788	-1.315583
C	-3.171898	0.748548	-1.310477
H	-3.695800	-1.220079	-2.071564
H	-3.553365	1.432461	-2.054932
N	-2.679362	-1.151194	-0.161248
N	-2.530856	1.230773	-0.156067
P	-1.840730	-0.009351	0.758087
C	-2.437198	2.685730	0.125151
C	-2.617244	-2.620764	0.035910
C	-1.708222	2.898935	1.450268
H	-0.685220	2.506661	1.413068
H	-1.646592	3.971310	1.652297
H	-2.240354	2.425270	2.280770
C	-1.658015	3.363678	-1.004996
H	-2.174977	3.260837	-1.963555
H	-1.546581	4.431160	-0.793696
H	-0.661779	2.920367	-1.097511
C	-3.851649	3.261767	0.225154
H	-4.401726	3.134012	-0.711552
H	-4.407726	2.766713	1.026830
H	-3.800792	4.332394	0.443661
C	-1.983381	-2.919523	1.393670
H	-1.970441	-4.001298	1.549335
H	-0.947124	-2.563817	1.440194
H	-2.552814	-2.462589	2.208999
C	-1.765984	-3.240972	-1.074959
H	-1.688857	-4.322568	-0.929421
H	-2.208930	-3.061135	-2.059088
H	-0.759864	-2.811183	-1.060256
C	-4.040003	-3.182662	0.004978
H	-4.649936	-2.717447	0.784982
H	-4.519658	-3.010164	-0.962763
H	-4.013459	-4.262292	0.178392

IM17$^+$

M06-2X/BS1 SCF energy in solution :
-447.515226 a.u.
M06-2X/BS2 SCF energy in solution:
-447.617860 a.u.
M06-2X/BS2 Free energy in solution:

-447.361328 a.u.

C	-1.237733	1.293520	-0.662553
C	-1.329566	-0.070759	0.033764
C	1.329531	-0.070843	0.033638
C	1.237786	1.293259	-0.663007
C	0.000167	2.094420	-0.291764
H	-1.250038	1.128107	-1.747017
H	-2.153630	1.833918	-0.404533
H	2.153889	1.833499	-0.405364
H	1.249657	1.127698	-1.747453
H	0.000352	2.344002	0.774686
H	0.000163	3.041251	-0.838620
N	-0.000043	-0.805992	-0.137578
C	-2.458664	-0.866034	-0.621186
H	-2.734205	-1.757776	-0.052569
H	-3.334713	-0.214012	-0.645538
H	-2.213294	-1.144782	-1.649906
C	-1.625039	0.062423	1.532047
H	-2.667129	0.376128	1.636127
H	-1.503542	-0.901336	2.034583
H	-1.003468	0.806167	2.029224
C	2.458326	-0.866716	-0.621070
H	3.334445	-0.214822	-0.646133
H	2.733905	-1.758062	-0.051843
H	2.212624	-1.146225	-1.649505
C	1.625270	0.063184	1.531859
H	1.500507	-0.899382	2.035872
H	2.668421	0.373428	1.635647
H	1.006311	0.809971	2.027792
C	-0.000053	-2.062758	-0.353138
H	-0.931205	-2.607004	-0.441954
H	0.931101	-2.607003	-0.441983

[NHP]OH

M06-2X/BS1 SCF energy in solution :
-919.546208 a.u.
M06-2X/BS2 SCF energy in solution:
-919.731162 a.u.
M06-2X/BS2 Free energy in solution:
-919.465252 a.u.

O	-0.000903	1.716106	1.059575
H	-0.000737	1.134081	1.839123
C	-0.670397	-1.482333	0.505225
C	0.670454	-1.482055	0.506154
H	-1.320148	-2.243625	0.913885
H	1.320109	-2.242989	0.915608
N	-1.199595	-0.353108	-0.151440
N	1.199736	-0.352408	-0.149777
P	0.000066	0.855010	-0.386898
C	2.654751	-0.089260	-0.148181
C	-2.654528	-0.089288	-0.148217
C	2.943832	1.168964	-0.968408
H	2.504711	2.060512	-0.508447
H	4.025448	1.320199	-1.022244
H	2.557639	1.073215	-1.987913
C	3.147249	0.116075	1.289746
H	2.968570	-0.777159	1.896513
H	4.221598	0.325067	1.300527
H	2.622573	0.959376	1.749667
C	3.376635	-1.276605	-0.792609
H	3.219114	-2.200773	-0.229320
H	3.018581	-1.429284	-1.815284
H	4.453328	-1.084955	-0.823648
C	-2.944191	1.168374	-0.969117
H	-4.025883	1.319049	-1.023141
H	-2.505664	2.060430	-0.509614
H	-2.557767	1.072244	-1.988504
C	-3.145478	0.117057	1.290090
H	-4.219878	0.325730	1.301968
H	-2.965740	-0.775728	1.897209
H	-2.620658	0.960853	1.748931
C	-3.377535	-1.276875	-0.790990
H	-3.020511	-1.430679	-1.813853
H	-3.220027	-2.200614	-0.227001
H	-4.454154	-1.084670	-0.821222

$TS20^{+}$

M06-2X/BS1 SCF energy in solution :
-1291.818134 a.u.
M06-2X/BS2 SCF energy in solution:
-1292.076815 a.u.
M06-2X/BS2 Free energy in solution:
-1291.540568 a.u.

C	-3.669169	0.141543	-1.329148
C	-2.794106	-1.102159	-1.125890

C	-2.521079	-0.264786	1.400350
C	-3.411390	0.914614	0.991268
C	-4.427543	0.572209	-0.085273
H	-3.031070	0.971052	-1.658955
H	-4.348361	-0.090297	-2.155112
H	-3.897000	1.268229	1.905870
H	-2.771683	1.730141	0.629321
H	-5.112247	-0.213816	0.252196
H	-5.037626	1.451219	-0.310972
N	-2.010778	-1.003464	0.173351
C	-1.820173	-1.182924	-2.305945
H	-1.304696	-2.143485	-2.377912
H	-2.400471	-1.056880	-3.223610
H	-1.079439	-0.378115	-2.257435
C	-3.647039	-2.372720	-1.058887
H	-4.137968	-2.517431	-2.025311
H	-3.018873	-3.245254	-0.856373
H	-4.418706	-2.310141	-0.288402
C	-1.332512	0.294458	2.185492
H	-1.722175	0.999062	2.925401
H	-0.774656	-0.471402	2.730435
H	-0.652818	0.830124	1.515307
C	-3.296159	-1.270798	2.258461
H	-2.669526	-2.136428	2.492945
H	-3.578041	-0.783658	3.196161
H	-4.206568	-1.619849	1.766571
C	-0.946002	-1.728381	0.298383
H	-0.674206	-2.430433	-0.480646
H	-0.456696	-1.810996	1.261580
C	2.839844	0.307783	1.353225
C	1.987728	1.345113	1.342859
H	3.463298	0.002812	2.182595
H	1.807530	2.033929	2.156003
N	2.922758	-0.359576	0.116404
N	1.295225	1.477595	0.121478
P	1.597137	0.113684	-0.871800
H	0.557106	-0.797794	-0.289127
C	0.857514	2.787433	-0.420474
C	3.577845	-1.679928	0.005337
C	-0.278935	2.533912	-1.412996
H	-1.084392	1.972676	-0.924849
H	-0.682248	3.484425	-1.773643
H	0.066414	1.966230	-2.283445
C	0.332873	3.659137	0.722095
H	1.128338	3.949695	1.413582
H	-0.091231	4.575497	0.302712
H	-0.452324	3.141306	1.282875
C	2.032145	3.486117	-1.112533
H	2.859290	3.622456	-0.408302
H	2.389408	2.889014	-1.958209
H	1.732064	4.469437	-1.488374
C	3.570580	-2.107350	-1.462785
H	4.122864	-3.044696	-1.569817
H	2.551885	-2.282059	-1.828673
H	4.045850	-1.349773	-2.093550
C	2.819745	-2.713356	0.847779
H	3.297188	-3.694381	0.764669
H	2.809591	-2.424415	1.903481
H	1.784249	-2.806366	0.502921
C	5.026276	-1.560257	0.485288
H	5.557681	-0.797829	-0.091893
H	5.083504	-1.298226	1.545405
H	5.536610	-2.518687	0.353309

2c

M06-2X/BS1 SCF energy in solution :
-448.264955 a.u.
M06-2X/BS2 SCF energy in solution:
-448.372771 a.u.
M06-2X/BS2 Free energy in solution:
-448.108715 a.u.

C	-1.237155	1.416481	-0.498877
C	-1.278672	-0.047875	-0.032012
C	1.278667	-0.047885	-0.032034
C	1.237171	1.416519	-0.498782
C	-0.000025	2.162103	-0.019592
H	-1.250281	1.425004	-1.597346
H	-2.153767	1.911987	-0.158839
H	2.153743	1.911992	-0.158583
H	1.250440	1.425177	-1.597248
H	-0.000080	2.244551	1.073909
H	-0.000024	3.185444	-0.409774
N	-0.000012	-0.711517	-0.405010
C	-2.426529	-0.724373	-0.800721
H	-2.721086	-1.681592	-0.361883
H	-3.307014	-0.074574	-0.778915
H	-2.143433	-0.888263	-1.845389

C	-1.628676	-0.108690	1.468379
H	-2.672242	0.192032	1.611233
H	-1.524833	-1.125501	1.860043
H	-1.010706	0.554615	2.076344
C	2.426471	-0.724375	-0.800827
H	3.306932	-0.074542	-0.779137
H	2.721108	-1.681552	-0.361946
H	2.143276	-0.888353	-1.845453
C	1.628775	-0.108761	1.468350
H	1.524383	-1.125456	1.860172
H	2.672523	0.191390	1.611080
H	1.011248	0.554993	2.076281
C	-0.000027	-2.141316	-0.125645
H	-0.000162	-2.401246	0.945038
H	0.874124	-2.613219	-0.576867
H	-0.874064	-2.613238	-0.577083

IM18⁻

M06-2X/BS1 SCF energy in solution :
-1017.082798 a.u.
M06-2X/BS2 SCF energy in solution:
-1017.324448 a.u.
M06-2X/BS2 Free energy in solution:
-1017.158058 a.u.

Si	-0.036464	0.598512	0.950858
C	1.342531	-0.475681	0.218236
C	1.635114	-0.492310	-1.155132
C	2.092032	-1.316359	1.052456
C	2.633878	-1.313318	-1.675315
H	1.076432	0.153181	-1.831768
C	3.093298	-2.143665	0.542778
H	1.888747	-1.312010	2.121045
C	3.365013	-2.142975	-0.824255
H	2.845555	-1.307044	-2.740803
H	3.662841	-2.785492	1.209072
H	4.144181	-2.784183	-1.225926
C	-1.670057	-0.024483	0.233487
C	-2.131754	0.329310	-1.043944
C	-2.452664	-0.917751	0.981304
C	-3.323242	-0.185060	-1.554065
H	-1.555065	1.028635	-1.644823
C	-3.644468	-1.439847	0.479791
H	-2.127116	-1.203154	1.979757
C	-4.081674	-1.073398	-0.792262
H	-3.662548	0.107713	-2.543745
H	-4.233108	-2.128347	1.079454
H	-5.010155	-1.475499	-1.187008
O	0.026674	0.796997	2.494079
O	0.121758	2.053690	-0.016270
C	1.280140	2.668294	-0.169872
H	2.135196	2.179038	0.330790
O	1.416150	3.688245	-0.804971

IM19⁻

M06-2X/BS1 SCF energy in solution :
-1770.881783 a.u.
M06-2X/BS2 SCF energy in solution:
-1771.255895 a.u.
M06-2X/BS2 Free energy in solution:
-1770.903896 a.u.

Si	-1.162408	0.154022	0.091188
C	-2.187098	-0.217937	-1.430849
C	-3.237602	0.613014	-1.848018
C	-1.928122	-1.381319	-2.172274
C	-4.009278	0.290263	-2.963841
H	-3.454575	1.528773	-1.301543
C	-2.695703	-1.709495	-3.287333
H	-1.107310	-2.032559	-1.877695
C	-3.739274	-0.872668	-3.682846
H	-4.817141	0.946209	-3.274110
H	-2.479092	-2.612037	-3.850955
H	-4.337698	-1.124687	-4.553227
C	-1.924931	-0.540499	1.645984
C	-3.294949	-0.416809	1.920255
C	-1.116613	-1.209029	2.576184
C	-3.840388	-0.936077	3.091978
H	-3.947072	0.087061	1.208811
C	-1.657132	-1.732223	3.750236
H	-0.054139	-1.319757	2.371091
C	-3.019692	-1.594274	4.008452
H	-4.902719	-0.831760	3.291107
H	-1.018115	-2.247045	4.461470
H	-3.443913	-2.001761	4.921269
O	0.356172	-0.327440	-0.030520
O	-1.325729	1.851948	0.308643
C	-0.879928	2.743206	-0.577248

H	-0.438781	2.307160	-1.491131
O	-0.970378	3.931775	-0.405785
Si	1.796435	0.069593	-1.112010
H	3.007840	0.399252	-2.065917
H	0.838150	0.182234	-2.278937
C	2.150887	1.671891	-0.102542
C	1.781655	1.836500	1.243264
C	2.790496	2.757280	-0.720693
C	2.040877	3.017952	1.937304
H	1.273857	1.021860	1.752625
C	3.024043	3.957895	-0.049679
H	3.111375	2.660066	-1.756234
C	2.653673	4.089119	1.287688
H	1.755694	3.108228	2.981969
H	3.501371	4.786518	-0.565521
H	2.842576	5.016840	1.820113
C	2.611429	-1.613542	-0.638445
C	1.948144	-2.633995	0.066964
C	3.942623	-1.867852	-1.006135
C	2.575414	-3.839453	0.381170
H	0.919341	-2.472223	0.371841
C	4.586962	-3.062985	-0.685536
H	4.489892	-1.108548	-1.559849
C	3.900964	-4.057599	0.008211
H	2.030334	-4.609721	0.920191
H	5.620696	-3.219571	-0.981213
H	4.393781	-4.993582	0.255149

TS21

M06-2X/BS1 SCF energy in solution :
-2614.499011 a.u.

M06-2X/BS2 SCF energy in solution:
-2615.021237 a.u.

M06-2X/BS2 Free energy in solution:
-2614.393840 a.u.

Si	-2.756711	-0.268686	0.470360
C	-3.026180	1.341953	1.385234
C	-3.525793	1.395189	2.696175
C	-2.760867	2.554757	0.729216
C	-3.748809	2.615614	3.331528
H	-3.741693	0.474774	3.235859
C	-2.978216	3.777655	1.361355
H	-2.373764	2.542629	-0.287359
C	-3.472331	3.808453	2.664510
H	-4.133491	2.636607	4.346660
H	-2.758624	4.703610	0.837841
H	-3.641130	4.759480	3.160685
C	-4.304544	-0.874698	-0.376055
C	-5.562073	-0.800985	0.240791
C	-4.223724	-1.430006	-1.660982
C	-6.703655	-1.275893	-0.400848
H	-5.655088	-0.363006	1.233069
C	-5.362955	-1.906014	-2.308288
H	-3.256024	-1.487603	-2.154146
C	-6.603406	-1.829936	-1.677089
H	-7.670007	-1.212356	0.090216
H	-5.284626	-2.334183	-3.303138
H	-7.492412	-2.199204	-2.179585
O	-1.556924	-0.222807	-0.587122
O	-2.503822	-1.449017	1.694539
C	-1.673987	-1.330224	2.731716
H	-1.127987	-0.373379	2.781139
O	-1.540270	-2.200259	3.554095
Si	0.221203	0.162713	-0.596194
H	1.829183	0.467135	-0.589893
H	0.260621	0.172761	0.908682
C	0.581977	-1.425103	-1.601084
C	-0.147042	-2.610904	-1.415017
C	1.636181	-1.461079	-2.526120
C	0.184083	-3.786499	-2.089951
H	-0.984552	-2.613172	-0.721931
C	1.954963	-2.619637	-3.234347
H	2.227625	-0.561176	-2.689842
C	1.237134	-3.793015	-3.003734
H	-0.381827	-4.695711	-1.907052
H	2.768475	-2.611798	-3.954368
H	1.493345	-4.704010	-3.536689
C	-0.087373	1.825820	-1.466115
C	0.466695	3.005492	-0.950826
C	-0.921984	1.936420	-2.587878
C	0.187932	4.250834	-1.514123
H	1.120120	2.950768	-0.080732
C	-1.181477	3.172508	-3.180538
H	-1.391137	1.042218	-2.992797
C	-0.634501	4.335337	-2.637456
H	0.612924	5.152360	-1.081384
H	-1.820951	3.232239	-4.056887

H	-0.847535	5.300481	-3.087464
C	3.336270	-0.139402	2.296354
C	3.944903	0.974826	1.808671
H	2.944558	-0.285175	3.293331
H	4.130305	1.900342	2.335576
N	3.299250	-1.148580	1.363401
N	4.371519	0.795283	0.515644
P	3.891590	-0.673315	-0.127939
C	5.083728	1.867461	-0.247256
C	2.827746	-2.531887	1.701743
C	5.450165	1.331802	-1.628823
H	4.558539	1.061630	-2.206045
H	5.973735	2.117784	-2.178337
H	6.115583	0.465076	-1.561890
C	4.150258	3.069283	-0.387064
H	3.846283	3.454778	0.590431
H	4.671481	3.867699	-0.922339
H	3.256947	2.793477	-0.954222
C	6.351395	2.231635	0.525250
H	6.116753	2.647329	1.508823
H	6.985475	1.350089	0.655996
H	6.909074	2.985523	-0.036754
C	2.977389	-3.422892	0.471112
H	2.591011	-4.416044	0.713587
H	2.402520	-3.038095	-0.377960
H	4.027780	-3.529767	0.180322
C	1.365126	-2.471818	2.137410
H	1.045162	-3.469715	2.449865
H	1.224493	-1.793394	2.983176
H	0.727973	-2.146907	1.309548
C	3.711907	-3.055575	2.835169
H	4.764935	-3.035047	2.540013
H	3.587349	-2.464635	3.746587
H	3.431004	-4.088143	3.058933

IM20

M06-2X/BS1 SCF energy in solution :
-1770.219120 a.u.

M06-2X/BS2 SCF energy in solution:
-1770.585931 a.u.

M06-2X/BS2 Free energy in solution:
-1770.242871 a.u.

Si	1.383417	0.095633	-0.177259
C	2.253701	0.913620	1.239457
C	1.997931	2.257323	1.555195
C	3.129963	0.185649	2.058432
C	2.598473	2.855263	2.660535
H	1.330491	2.842499	0.926879
C	3.733658	0.782524	3.163130
H	3.350869	-0.854716	1.829648
C	3.466309	2.116975	3.464351
H	2.393318	3.895544	2.893207
H	4.412469	0.208874	3.786400
H	3.936706	2.583202	4.324721
C	2.347933	-1.261320	-0.991786
C	3.731436	-1.137710	-1.193581
C	1.702894	-2.422927	-1.441849
C	4.450155	-2.146042	-1.831058
H	4.256075	-0.250935	-0.843714
C	2.419870	-3.432581	-2.080110
H	0.632543	-2.537613	-1.289172
C	3.793179	-3.293263	-2.274635
H	5.520152	-2.039625	-1.979618
H	1.909653	-4.327133	-2.423407
H	4.352921	-4.080481	-2.770447
O	-0.078027	-0.536915	0.238591
O	1.095421	1.377482	-1.255656
C	0.288480	1.308124	-2.327742
H	-0.226192	0.341576	-2.453842
O	0.159497	2.226972	-3.088813
Si	-1.475587	0.081261	0.916464
H	-1.113409	0.719731	2.206537
C	-2.192796	1.391757	-0.200975
C	-3.003051	1.050829	-1.295760
C	-1.853619	2.740748	-0.016116
C	-3.456870	2.026477	-2.180317
H	-3.286301	0.012607	-1.457140
C	-2.302790	3.719405	-0.900496
H	-1.231553	3.032672	0.828036
C	-3.104242	3.360952	-1.983037
H	-4.084313	1.748264	-3.021546
H	-2.031494	4.759061	-0.744696
H	-3.456485	4.122289	-2.672459
C	-2.626747	-1.371287	1.115410
C	-2.127242	-2.681757	1.129141
C	-4.008525	-1.179705	1.268634
C	-2.982387	-3.769851	1.293296

H	-1.060468	-2.852264	1.006113
C	-4.865817	-2.265008	1.435408
H	-4.423739	-0.174005	1.248913
C	-4.351914	-3.561243	1.447633
H	-2.581774	-4.778884	1.299331
H	-5.932758	-2.101094	1.550812
H	-5.019356	-4.408139	1.574635

TS22

M06-2X/BS1 SCF energy in solution :
-1673.301883 a.u.
M06-2X/BS2 SCF energy in solution:
-1673.623526 a.u.
M06-2X/BS2 Free energy in solution:
-1673.174511 a.u.

O	0.330353	0.498188	-0.565928
H	0.561376	0.479582	-1.509393
C	3.759104	0.212958	-1.039749
C	3.408559	-1.087870	-0.989006
H	4.455911	0.680992	-1.720115
H	3.759398	-1.889225	-1.623010
N	3.122777	0.953862	-0.047361
N	2.509362	-1.336189	0.044372
P	1.993809	0.061981	0.804732
C	1.895228	-2.680911	0.244953
C	3.291480	2.431642	0.059665
C	0.995129	-2.630975	1.477628
H	0.182651	-1.905382	1.355258
H	0.539052	-3.613709	1.622549
H	1.567022	-2.380397	2.377120
C	1.066470	-3.019874	-0.996470
H	1.703242	-3.118283	-1.880737
H	0.539339	-3.966466	-0.844174
H	0.325666	-2.236335	-1.182744
C	3.009967	-3.703885	0.461122
H	3.660183	-3.785788	-0.414171
H	3.618243	-3.430276	1.328106
H	2.565556	-4.686538	0.641981
C	2.491107	2.939861	1.257291
H	2.634782	4.019803	1.340752
H	1.417855	2.755269	1.133947
H	2.830026	2.477846	2.190002
C	2.766207	3.073556	-1.225900
H	2.863533	4.160761	-1.159582
H	3.331651	2.733145	-2.098032
H	1.711035	2.823672	-1.370677
C	4.775056	2.738415	0.261985
H	5.148113	2.242189	1.162628
H	5.374059	2.413459	-0.593137
H	4.909078	3.817613	0.375929
Si	-1.432469	0.293277	-0.293379
H	-1.034988	0.299342	1.208942
C	-2.283626	-1.428352	-0.163130
C	-2.649678	-2.159356	-1.302849
C	-2.564242	-2.007245	1.084276
C	-3.259018	-3.411016	-1.209083
H	-2.443943	-1.741157	-2.286818
C	-3.182229	-3.252151	1.195330
H	-2.282137	-1.470560	1.989198
C	-3.526736	-3.960575	0.044012
H	-3.523089	-3.958600	-2.109579
H	-3.389443	-3.674042	2.174934
H	-3.999777	-4.935045	0.123556
C	-2.637476	1.776149	-0.087573
C	-2.944890	2.293927	1.179981
C	-3.269406	2.368949	-1.191117
C	-3.846255	3.344905	1.345112
H	-2.465378	1.862022	2.056781
C	-4.170029	3.423352	-1.042539
H	-3.049553	1.995625	-2.189804
C	-4.460922	3.913714	0.229942
H	-4.067648	3.723654	2.339138
H	-4.643398	3.864399	-1.915439
H	-5.160490	4.735599	0.351671
H	-1.541092	0.310712	-1.853699

IM21^{-}

M06-2X/BS1 SCF energy in solution :
-829.682816 a.u.
M06-2X/BS2 SCF energy in solution:
-829.858886 a.u.
M06-2X/BS2 Free energy in solution:
-829.685342 a.u.

O	-0.001040	3.042756	0.187575
H	-0.001079	3.291815	1.120426
Si	-0.000030	1.324899	0.081015

H	0.000178	1.504926	-1.478082
C	-1.611469	0.250192	0.011364
C	-2.164529	-0.328301	1.164505
C	-2.271227	-0.007501	-1.200268
C	-3.307310	-1.126640	1.117872
H	-1.683608	-0.145647	2.124100
C	-3.418685	-0.798466	-1.265714
H	-1.873187	0.425345	-2.116493
C	-3.940479	-1.362517	-0.102200
H	-3.707770	-1.562088	2.029596
H	-3.907302	-0.974918	-2.220319
H	-4.834084	-1.978685	-0.145433
C	1.611625	0.250364	0.011361
C	2.271546	-0.007151	-1.200206
C	2.164603	-0.328197	1.164517
C	3.419074	-0.798017	-1.265603
H	1.873572	0.425773	-2.116430
C	3.307444	-1.126451	1.117939
H	1.683542	-0.145675	2.124066
C	3.940777	-1.362146	-0.102088
H	3.907828	-0.974322	-2.220165
H	3.707833	-1.561968	2.029661
H	4.834443	-1.978228	-0.145287
H	0.000166	1.252025	1.670665

TS23

M06-2X/BS1 SCF energy in solution :
-1673.303659 a.u.
M06-2X/BS2 SCF energy in solution:
-1673.625566 a.u.
M06-2X/BS2 Free energy in solution:
-1673.173526 a.u.

Si	-0.965489	0.913305	0.958534
H	-0.544615	-0.112870	-0.285995
C	-0.138175	2.300035	-0.079043
C	-0.114203	2.247975	-1.482088
C	0.495609	3.390554	0.533596
C	0.511789	3.234421	-2.242053
H	-0.585463	1.404994	-1.986648
C	1.158890	4.366250	-0.212768
H	0.481073	3.472496	1.619527
C	1.165838	4.289876	-1.604679
H	0.505993	3.175169	-3.326875
H	1.663107	5.187854	0.288325
H	1.675100	5.049623	-2.190409
C	-2.828051	0.668987	0.573417
C	-3.290514	0.120516	-0.633291
C	-3.799058	1.074589	1.502033
C	-4.650853	-0.011932	-0.907353
H	-2.566031	-0.211972	-1.374347
C	-5.163844	0.934495	1.247980
H	-3.480226	1.510918	2.446456
C	-5.593257	0.392806	0.038017
H	-4.977813	-0.432618	-1.854164
H	-5.891059	1.251345	1.990378
H	-6.654319	0.285527	-0.167601
C	2.421980	-1.907907	1.231392
C	1.497363	-2.839920	0.914686
H	3.094617	-1.912658	2.076521
H	1.276704	-3.750953	1.451971
N	2.443543	-0.878477	0.306038
N	0.810484	-2.511670	-0.236866
P	1.222774	-0.995900	-0.842463
C	-0.347104	-3.317330	-0.718116
C	3.430966	0.235044	0.427898
C	0.074291	-4.783744	-0.815872
H	0.312921	-5.207587	0.162942
H	-0.751809	-5.362986	-1.237426
H	0.944885	-4.891143	-1.469656
C	-1.499012	-3.139208	0.273540
H	-1.777395	-2.084165	0.349800
H	-2.367838	-3.711828	-0.064114
H	-1.212783	-3.496680	1.267302
C	-0.755644	-2.815729	-2.102476
H	-1.067736	-1.765782	-2.075530
H	0.060980	-2.929111	-2.822458
H	-1.607861	-3.402691	-2.453926
C	4.836146	-0.371236	0.444690
H	5.574166	0.434672	0.484326
H	4.991659	-1.013783	1.315221
H	5.009755	-0.958675	-0.461809
C	3.146922	0.997504	1.722554
H	3.853908	1.826646	1.819844
H	2.129736	1.397461	1.709902
H	3.256852	0.349886	2.597361
C	3.297741	1.171324	-0.770146
H	3.492035	0.645483	-1.710828

H	2.305558	1.629272	-0.812724
H	4.034443	1.972805	-0.670454
O	-0.087397	-0.223975	1.884783
H	-0.012743	0.069961	2.802176
H	-1.216498	1.832229	2.186400

IM22

M06-2X/BS1 SCF energy in solution :
-829.025995 a.u.
M06-2X/BS2 SCF energy in solution:
-829.194248 a.u.
M06-2X/BS2 Free energy in solution:
-829.029522 a.u.

Si	-0.004923	1.317694	-0.370638
C	-1.527382	0.246591	-0.216865
C	-2.716456	0.771237	0.311331
C	-1.522983	-1.089164	-0.645952
C	-3.865221	-0.011804	0.406362
H	-2.740487	1.800725	0.659152
C	-2.670447	-1.875016	-0.556681
H	-0.611484	-1.526966	-1.047794
C	-3.843009	-1.335787	-0.029677
H	-4.776155	0.409033	0.821246
H	-2.648928	-2.907216	-0.892584
H	-4.737359	-1.947203	0.043681
C	1.542159	0.300700	-0.109149
C	2.671296	0.491088	-0.918808
C	1.613402	-0.655973	0.916806
C	3.838024	-0.242790	-0.708684
H	2.641447	1.220243	-1.725788
C	2.776413	-1.390765	1.132193
H	0.746479	-0.834963	1.549878
C	3.890763	-1.182396	0.318839
H	4.703138	-0.082839	-1.344986
H	2.815225	-2.127227	1.929128
H	4.797974	-1.755664	0.484563
O	-0.224227	2.501121	0.778363
H	0.438187	3.202830	0.804671
H	0.115350	1.937043	-1.717426

TS4'

M06-2X/BS1 SCF energy in solution :
-1786.574366 a.u.
M06-2X/BS2 SCF energy in solution:
-1786.932340 a.u.
M06-2X/BS2 Free energy in solution:
-1786.478740 a.u.

Si	-2.055109	-0.725870	-0.711569
H	-0.317703	-0.325917	-1.023428
H	-2.122395	-1.020897	-2.179523
C	-2.271434	1.113976	-0.323821
C	-1.962876	2.098726	-1.274198
C	-2.756559	1.538127	0.921233
C	-2.141449	3.453445	-1.000200
H	-1.571611	1.801002	-2.246024
C	-2.906032	2.893897	1.217874
H	-3.025593	0.799375	1.674410
C	-2.606888	3.854443	0.253293
H	-1.911271	4.196928	-1.758305
H	-3.267485	3.199726	2.195511
H	-2.736022	4.909608	0.475474
C	-3.805150	-1.393608	-0.242783
C	-4.857402	-1.262014	-1.161942
C	-4.114684	-1.969667	0.999884
C	-6.155866	-1.674305	-0.863355
H	-4.658367	-0.828997	-2.142853
C	-5.408902	-2.387768	1.313163
H	-3.323405	-2.099137	1.734815
C	-6.434746	-2.239668	0.380526
H	-6.948264	-1.560177	-1.598116
H	-5.617998	-2.832133	2.282646
H	-7.443005	-2.564985	0.619889
C	3.959619	-0.347930	1.318864
C	3.416210	0.901229	1.384392
H	4.488159	-0.870934	2.103352
H	3.420598	1.569759	2.233717
N	3.768842	-0.914619	0.092418
N	2.826942	1.256405	0.206859
P	2.891740	0.053247	-0.954725
C	2.072964	2.543419	0.053140
C	4.214316	-2.312824	-0.211039
C	1.598934	2.666281	-1.391892
H	0.907062	1.858396	-1.655752
H	1.062063	3.611789	-1.502260
H	2.441906	2.670036	-2.091255
C	0.875619	2.479431	1.001668

H	1.203607	2.451550	2.045136
H	0.252023	3.366515	0.858302
H	0.286543	1.580959	0.795748
C	3.009314	3.700460	0.395837
H	3.350717	3.653211	1.433113
H	3.880731	3.701171	-0.265277
H	2.468726	4.641227	0.262391
C	3.816066	-2.663864	-1.641406
H	4.168617	-3.674601	-1.859398
H	2.728502	-2.653102	-1.771973
H	4.274452	-1.984275	-2.367317
C	3.508845	-3.244955	0.774608
H	3.738574	-4.281855	0.515722
H	3.844774	-3.066527	1.799710
H	2.426857	-3.091668	0.729901
C	5.733078	-2.375020	-0.058421
H	6.216637	-1.683084	-0.753892
H	6.043692	-2.128939	0.960385
H	6.071597	-3.390343	-0.280986
O	0.818928	-0.890216	1.040125
C	0.128743	-1.403985	0.164119
H	0.570372	-2.078246	-0.592418
O	-1.212488	-1.698712	0.397273

TS4''

M06-2X/BS1 SCF energy in solution :
-1975.113289 a.u.
M06-2X/BS2 SCF energy in solution:
-1975.533949 a.u.
M06-2X/BS2 Free energy in solution:
-1975.069338 a.u.

C	-4.211464	-0.066680	-1.134300
C	-3.565210	1.112611	-1.357345
H	-5.016462	-0.489366	-1.718473
H	-3.757883	1.820204	-2.151134
N	-3.720397	-0.706713	-0.032919
N	-2.594442	1.339825	-0.423507
P	-2.446545	0.099907	0.693427
C	-1.740541	2.573255	-0.444921
C	-4.215301	-2.056623	0.389140
C	-0.821130	2.550418	0.772555
H	-0.200348	1.648988	0.793008
H	-0.151644	3.414498	0.722957
H	-1.393151	2.613828	1.704653
C	-0.915566	2.565453	-1.732157
H	-1.556261	2.527097	-2.617694
H	-0.322855	3.483635	-1.777898
H	-0.232391	1.711950	-1.748123
C	-2.662564	3.791148	-0.388488
H	-2.051501	4.695520	-0.326459
H	-3.287513	3.866348	-1.282436
H	-3.307110	3.743505	0.493883
C	-5.711036	-1.947899	0.681889
H	-6.079559	-2.921708	1.015023
H	-5.896825	-1.214307	1.471601
H	-6.274962	-1.660140	-0.209469
C	-3.464988	-2.490964	1.645643
H	-3.841907	-3.469677	1.951851
H	-2.389108	-2.589761	1.463823
H	-3.628039	-1.793677	2.474192
C	-3.936460	-3.034186	-0.752869
H	-4.502113	-2.767792	-1.650039
H	-2.871281	-3.035375	-0.997733
H	-4.237632	-4.039036	-0.444759
O	-1.398911	-1.086531	-1.971546
C	-0.206542	-0.919024	-1.814686
H	0.515000	-1.101002	-2.629119
O	0.322369	-0.520770	-0.677261
Si	2.069151	-0.171036	-0.448823
H	1.723075	-0.551500	1.078309
H	2.198476	0.130613	-1.949367
C	3.407378	-1.537749	-0.456929
C	4.114212	-1.829111	-1.633748
C	3.744844	-2.260751	0.697164
C	5.122907	-2.791420	-1.659768
H	3.870535	-1.289871	-2.547070
C	4.752119	-3.224046	0.683977
H	3.203341	-2.066703	1.620680
C	5.443728	-3.491002	-0.497368
H	5.656548	-2.997465	-2.583236
H	4.996160	-3.770069	1.590815
H	6.226928	-4.243406	-0.512406
C	2.508655	1.599617	0.109780
C	2.637801	2.635963	-0.826186
C	2.660884	1.925320	1.466380
C	2.895792	3.948640	-0.429258
H	2.524017	2.413713	-1.885538

C	2.932352	3.230105	1.874071
H	2.541249	1.143944	2.213847
C	3.043625	4.246953	0.924630
H	2.982103	4.736259	-1.172536
H	3.047836	3.459169	2.929681
H	3.243942	5.267002	1.239291
C	0.488798	-1.060365	2.109015
O	0.236688	-0.060444	2.692831
O	0.388541	-2.210915	1.850931

TS9s$^-$

M06-2X/BS1 SCF energy in solution :
-1533.581128 a.u.
M06-2X/BS2 SCF energy in solution:
-1533.966209 a.u.
M06-2X/BS2 Free energy in solution:
-1533.629631 a.u.

C	1.055294	-1.332519	0.249868
O	0.706498	-2.143477	1.148201
O	0.132726	-0.352705	-0.149580
H	3.223194	-0.959270	0.845062
H	1.625516	-1.675634	-0.625784
Si	-1.353365	-0.246928	0.605299
N	2.387954	-0.288454	0.892133
C	2.105175	-0.005976	2.298418
H	2.063046	-0.962418	2.819481
H	2.890004	0.618893	2.733123
H	1.138409	0.491611	2.408119
C	-1.922504	1.509631	0.350144
C	-2.971802	2.056918	1.105016
C	-1.292820	2.325239	-0.601647
C	-3.380596	3.375361	0.914264
H	-3.476241	1.450286	1.854496
C	-1.695571	3.646044	-0.793598
H	-0.469857	1.924125	-1.188544
C	-2.741126	4.171186	-0.035959
H	-4.193339	3.783507	1.507485
H	-1.192210	4.264818	-1.530711
H	-3.056172	5.199904	-0.183145
H	5.269919	-3.500812	-0.068661
C	4.487373	-2.699433	-0.097181
O	4.465436	-1.949529	0.918745
O	3.764075	-2.654680	-1.107165
C	2.618870	0.830915	0.040033
C	2.165871	2.114608	0.351216
C	3.269369	0.593143	-1.176481
C	2.365305	3.155299	-0.554773
H	1.654320	2.307748	1.288035
C	3.460636	1.640497	-2.072552
H	3.602336	-0.418611	-1.402010
C	3.010793	2.925636	-1.767825
H	2.008175	4.150680	-0.307275
H	3.966078	1.451808	-3.015077
H	3.162964	3.739885	-2.469095
C	-2.531937	-1.468594	-0.190975
C	-2.141892	-2.803110	-0.398288
C	-3.820258	-1.087406	-0.593495
C	-3.010800	-3.721119	-0.983271
H	-1.148291	-3.120665	-0.089701
C	-4.694123	-2.003841	-1.178032
H	-4.149720	-0.059948	-0.455388
C	-4.289213	-3.322152	-1.373999
H	-2.692315	-4.748076	-1.136483
H	-5.687370	-1.688165	-1.483100
H	-4.966544	-4.037242	-1.831386
O	-1.203496	-0.513563	2.233365
H	-0.538651	-1.226591	2.317612

IM9s$^-$

M06-2X/BS1 SCF energy in solution :
-1533.581765 a.u.
M06-2X/BS2 SCF energy in solution:
-1533.966120 a.u.
M06-2X/BS2 Free energy in solution:
-1533.629306 a.u.

C	1.113153	-1.086880	0.295836
O	0.773847	-1.942692	1.188765
O	0.113936	-0.131840	-0.079949
H	3.176222	-0.818507	0.942853
H	1.577658	-1.460957	-0.633661
Si	-1.395052	-0.241164	0.616550
N	2.317095	-0.126872	0.847419
C	2.005500	0.321170	2.212838
H	1.930353	-0.571132	2.832333
H	2.807166	0.965686	2.577757
H	1.053586	0.855347	2.239293

C	-2.159695	1.449810	0.406471
C	-3.391163	1.781056	0.993886
C	-1.488744	2.439065	-0.327368
C	-3.934976	3.056067	0.852012
H	-3.937016	1.035686	1.569614
C	-2.027872	3.716939	-0.472023
H	-0.529887	2.204206	-0.783345
C	-3.252116	4.025885	0.117728
H	-4.888243	3.294920	1.313880
H	-1.492595	4.470750	-1.042096
H	-3.674121	5.020326	0.007354
H	5.169683	-3.412582	0.606958
C	4.455313	-2.594013	0.343736
O	4.314732	-1.714206	1.246663
O	3.910716	-2.641935	-0.768238
C	2.658982	0.910754	-0.093737
C	2.222567	2.226301	0.061193
C	3.416265	0.541875	-1.207161
C	2.551437	3.175578	-0.904236
H	1.627290	2.513671	0.920996
C	3.737424	1.500101	-2.166106
H	3.735498	-0.494197	-1.305531
C	3.308322	2.817896	-2.018873
H	2.210620	4.199292	-0.782212
H	4.328186	1.214306	-3.031058
H	3.562052	3.562216	-2.766914
C	-2.414104	-1.554831	-0.257665
C	-1.942083	-2.877754	-0.330906
C	-3.648690	-1.263269	-0.855738
C	-2.679506	-3.868437	-0.974715
H	-0.983001	-3.119737	0.122560
C	-4.391382	-2.252844	-1.500358
H	-4.040117	-0.249101	-0.824180
C	-3.907222	-3.557437	-1.560044
H	-2.298052	-4.884306	-1.021924
H	-5.344873	-2.004467	-1.957050
H	-4.482295	-4.329471	-2.062709
O	-1.250558	-0.566720	2.233425
H	-0.486901	-1.190617	2.259518

TS10s⁻

M06-2X/BS1 SCF energy in solution :
-1533.581168 a.u.
M06-2X/BS2 SCF energy in solution:
-1533.962366 a.u.
M06-2X/BS2 Free energy in solution:
-1533.624627 a.u.

C	1.113049	-0.600816	0.321527
O	0.665005	-1.472400	1.189241
O	0.093006	0.386288	0.006254
H	3.276764	-0.997807	1.196146
H	1.458815	-1.009042	-0.646558
Si	-1.351012	-0.118961	0.671411
N	2.314142	0.180097	0.848003
C	2.018413	0.773138	2.150308
H	1.710808	-0.037033	2.811822
H	2.918747	1.246118	2.550325
H	1.212352	1.514894	2.102730
C	-2.499481	1.335961	0.330087
C	-3.329229	1.861068	1.330875
C	-2.561079	1.924743	-0.943196
C	-4.189916	2.929251	1.074408
H	-3.295863	1.429409	2.328295
C	-3.414228	2.994112	-1.208167
H	-1.930594	1.542657	-1.743995
C	-4.233272	3.497554	-0.197055
H	-4.824034	3.319075	1.865618
H	-3.442402	3.435440	-2.200293
H	-4.900488	4.330043	-0.400403
H	4.466259	-3.632677	1.120789
C	3.912196	-2.770508	0.711701
O	3.893253	-1.767716	1.550746
O	3.411970	-2.795594	-0.396409
C	2.893906	1.053843	-0.117169
C	2.885734	2.446464	0.011899
C	3.515157	0.477589	-1.235958
C	3.482339	3.244939	-0.963780
H	2.416509	2.914047	0.870346
C	4.095536	1.282270	-2.210194
H	3.549262	-0.605926	-1.315429
C	4.083726	2.671683	-2.080741
H	3.469759	4.324610	-0.846754
H	4.573132	0.820332	-3.069328
H	4.544632	3.297851	-2.838016
C	-2.099054	-1.607477	-0.226689
C	-1.377647	-2.778575	-0.521089
C	-3.439956	-1.554147	-0.644095

C	-1.968657	-3.839553	-1.204979
H	-0.347752	-2.846708	-0.182202
C	-4.039926	-2.617190	-1.319141
H	-4.034378	-0.666216	-0.441770
C	-3.302108	-3.763239	-1.605543
H	-1.388267	-4.731766	-1.423255
H	-5.080127	-2.547761	-1.624150
H	-3.762868	-4.591826	-2.135488
O	-1.381340	-0.235561	2.330753
H	-0.603274	-0.789738	2.523060

IM10s$^-$

M06-2X/BS1 SCF energy in solution :
-1533.591032 a.u.
M06-2X/BS2 SCF energy in solution:
-1533.969497 a.u.
M06-2X/BS2 Free energy in solution:
-1533.630875 a.u.

C	-0.914133	-0.521017	-0.275257
O	-0.128564	-0.852809	-1.367734
O	-0.025585	0.127130	0.543475
H	-2.740012	-0.482548	-1.866286
H	-1.386138	-1.401056	0.185991
Si	1.349647	-0.223474	-0.631151
N	-2.046395	0.360504	-0.714795
C	-1.554694	1.548725	-1.417985
H	-1.025679	1.214976	-2.311578
H	-2.405119	2.164168	-1.718805
H	-0.869792	2.144420	-0.804492
C	2.106965	1.527321	-0.387708
C	3.236794	1.940358	-1.116086
C	1.611459	2.428346	0.571244
C	3.838743	3.180707	-0.904338
H	3.642965	1.272376	-1.869098
C	2.203470	3.672850	0.790200
H	0.735128	2.144168	1.146529
C	3.322198	4.053990	0.051652
H	4.710087	3.467677	-1.487005
H	1.790980	4.346607	1.536460
H	3.786539	5.021920	0.218062
H	-3.580617	-2.785185	-3.383263
C	-3.233265	-2.248147	-2.485207
O	-3.138167	-0.959531	-2.699968
O	-2.988544	-2.821371	-1.441608
C	-2.956806	0.618007	0.360640
C	-3.081723	1.872633	0.963324
C	-3.755515	-0.442091	0.810955
C	-3.995269	2.060936	2.000502
H	-2.473356	2.705741	0.628770
C	-4.654654	-0.247968	1.853849
H	-3.667567	-1.409373	0.322739
C	-4.781541	1.005435	2.453540
H	-4.085139	3.041733	2.457715
H	-5.269049	-1.077326	2.191245
H	-5.489883	1.157174	3.261661
C	2.224317	-1.505957	0.481793
C	1.750715	-1.838952	1.761823
C	3.394137	-2.152389	0.048104
C	2.405482	-2.770863	2.567482
H	0.848658	-1.353132	2.123195
C	4.060007	-3.082876	0.845618
H	3.781198	-1.917580	-0.939846
C	3.565166	-3.395745	2.110986
H	2.012857	-3.010327	3.552138
H	4.962939	-3.565493	0.481215
H	4.079005	-4.120578	2.736029
O	2.217265	-0.487005	-2.102458
H	1.829357	-1.224383	-2.586137

TS11s$^-$

M06-2X/BS1 SCF energy in solution :
-1533.586661 a.u.
M06-2X/BS2 SCF energy in solution:
-1533.966172 a.u.
M06-2X/BS2 Free energy in solution:
-1533.627339 a.u.

C	0.868938	-0.359315	-0.014188
O	0.194241	-0.542652	1.200512
O	-0.142654	0.084189	-0.828470
H	1.948664	0.022865	2.154396
H	1.332196	-1.293091	-0.364370
Si	-1.384144	-0.151026	0.523212
N	1.957876	0.617054	0.171407
C	1.471058	1.979475	0.329339
H	0.638150	1.968682	1.036255
H	2.266530	2.603427	0.745086

H	1.114459	2.424199	-0.608284
C	-2.214308	1.540989	0.162590
C	-3.115108	2.134320	1.063517
C	-1.984537	2.224728	-1.044647
C	-3.749386	3.344979	0.781975
H	-3.312721	1.632206	2.005095
C	-2.618400	3.431266	-1.340360
H	-1.282271	1.801829	-1.756790
C	-3.503755	3.998021	-0.424225
H	-4.435436	3.779452	1.504253
H	-2.418701	3.933699	-2.283070
H	-3.996105	4.940207	-0.647965
H	3.531323	-1.164824	4.169137
C	2.999930	-1.140558	3.206336
O	2.418608	0.031467	3.021163
O	2.967481	-2.074759	2.439089
C	3.128295	0.436286	-0.591159
C	3.693459	1.469764	-1.352604
C	3.791197	-0.805067	-0.553319
C	4.871108	1.258231	-2.069352
H	3.215602	2.441805	-1.395310
C	4.953732	-1.008917	-1.285636
H	3.405099	-1.589128	0.090472
C	5.504485	0.019714	-2.052500
H	5.287987	2.074817	-2.651832
H	5.446622	-1.975742	-1.239225
H	6.418448	-0.140723	-2.615096
C	-2.279302	-1.600036	-0.346921
C	-1.916679	-2.049206	-1.628157
C	-3.354710	-2.256764	0.275495
C	-2.586561	-3.099745	-2.255422
H	-1.088651	-1.559788	-2.132327
C	-4.034167	-3.306475	-0.342421
H	-3.655379	-1.932409	1.267852
C	-3.650110	-3.732211	-1.613230
H	-2.279853	-3.426155	-3.245604
H	-4.861634	-3.794024	0.166050
H	-4.174843	-4.550086	-2.098850
O	-2.118098	-0.292108	2.083157
H	-1.634292	-0.928212	2.621046

IM11s⁻

M06-2X/BS1 SCF energy in solution :
-1533.594995 a.u.

M06-2X/BS2 SCF energy in solution:
-1533.972944 a.u.

M06-2X/BS2 Free energy in solution:
-1533.633899 a.u.

C	0.984604	0.369691	-0.547293
O	0.621198	0.073072	0.796666
O	-0.234100	0.747358	-1.051074
H	0.929582	-1.232710	1.442574
H	1.383558	-0.528719	-1.035765
Si	-1.140218	0.171163	0.423594
N	1.982791	1.411894	-0.636859
C	1.536696	2.761154	-0.321909
H	0.447787	2.775397	-0.358517
H	1.868574	3.080186	0.674447
H	1.908471	3.483502	-1.055515
C	-2.471202	1.539242	0.230338
C	-3.489942	1.704774	1.185942
C	-2.517539	2.380807	-0.894816
C	-4.496560	2.657333	1.032042
H	-3.482250	1.075428	2.069856
C	-3.519896	3.337711	-1.058856
H	-1.745366	2.284666	-1.651583
C	-4.514192	3.480325	-0.093172
H	-5.266967	2.760245	1.791512
H	-3.522961	3.974197	-1.939579
H	-5.295110	4.225443	-0.215547
H	0.775065	-4.092149	1.405228
C	1.036897	-3.096830	1.013297
O	0.852810	-2.151728	1.909868
O	1.465660	-2.927385	-0.109625
C	3.307872	1.082217	-0.356546
C	4.291294	2.091541	-0.321688
C	3.722846	-0.243501	-0.104518
C	5.625912	1.784515	-0.078069
H	4.012316	3.124121	-0.497236
C	5.063489	-0.531980	0.130438
H	3.006549	-1.057338	-0.071827
C	6.030991	0.470744	0.145480
H	6.355340	2.589515	-0.063801
H	5.347911	-1.563179	0.320966
H	7.072454	0.234886	0.337180
C	-1.678041	-1.509893	-0.294199
C	-1.479926	-1.840332	-1.644272

C	-2.245278	-2.497395	0.527960
C	-1.826308	-3.092551	-2.151784
H	-1.034936	-1.098593	-2.302128
C	-2.582894	-3.758023	0.035897
H	-2.417463	-2.272981	1.577277
C	-2.374091	-4.058873	-1.309538
H	-1.660907	-3.319307	-3.201546
H	-3.011604	-4.504449	0.699143
H	-2.637708	-5.038463	-1.698055
O	-1.511872	-0.071194	2.086983
H	-0.926773	-0.724286	2.489663

TS12s$^-$

M06-2X/BS1 SCF energy in solution :
-1533.581496 a.u.
M06-2X/BS2 SCF energy in solution:
-1533.963471 a.u.
M06-2X/BS2 Free energy in solution:
-1533.626263 a.u.

C	0.801715	-0.700059	-1.072492
O	0.427309	-0.292295	0.454685
O	-0.170244	-0.375828	-1.831108
H	1.241156	0.834325	0.891371
H	1.007381	-1.775721	-0.906063
Si	-1.224988	-0.113095	0.634069
N	2.070932	-0.038586	-1.319751
C	2.050210	1.201893	-2.064616
H	1.004983	1.430637	-2.271246
H	2.501112	2.016000	-1.485562
H	2.581545	1.119418	-3.021282
C	-1.916229	1.455239	-0.147739
C	-1.940508	2.635385	0.610243
C	-2.446437	1.500051	-1.446164
C	-2.457291	3.822580	0.089008
H	-1.548008	2.629368	1.624933
C	-2.981181	2.675753	-1.966785
H	-2.417553	0.602847	-2.057485
C	-2.983552	3.842430	-1.200251
H	-2.455539	4.726830	0.690825
H	-3.392357	2.687468	-2.972193
H	-3.394665	4.761419	-1.607817
H	2.113045	3.075185	2.527106
C	1.481499	2.212235	2.268016
O	1.832101	1.665178	1.141957
O	0.569266	1.853689	2.999777
C	3.245394	-0.481559	-0.742046
C	4.490876	0.023145	-1.172474
C	3.251720	-1.443541	0.293364
C	5.679823	-0.440233	-0.618576
H	4.528365	0.773999	-1.953556
C	4.451101	-1.901130	0.827315
H	2.315137	-1.801934	0.702067
C	5.678223	-1.412405	0.379610
H	6.620421	-0.032904	-0.979142
H	4.420212	-2.641525	1.622136
H	6.608473	-1.771206	0.807316
C	-2.238077	-1.652188	0.248782
C	-2.255211	-2.320977	-0.987324
C	-3.053225	-2.159711	1.275865
C	-3.052379	-3.447780	-1.184783
H	-1.621205	-1.944379	-1.785051
C	-3.854992	-3.283635	1.080130
H	-3.053381	-1.668654	2.244837
C	-3.856499	-3.930963	-0.153633
H	-3.046101	-3.951458	-2.147408
H	-4.475394	-3.655004	1.890774
H	-4.477820	-4.808064	-0.310032
O	-1.437658	0.079028	2.272490
H	-0.742569	0.641439	2.661663

TS12-1a

M06-2X/BS1 SCF energy in solution :
-1784.425345 a.u.
M06-2X/BS2 SCF energy in solution:
-1784.848424 a.u.
M06-2X/BS2 Free energy in solution:
-1784.384953 a.u.

Si	2.053980	-0.261218	0.198966
C	2.512521	-1.850205	-0.686066
C	3.834295	-2.033961	-1.125184
C	1.583306	-2.871880	-0.952511
C	4.219201	-3.194454	-1.795359
H	4.578783	-1.261750	-0.950333
C	1.965770	-4.031612	-1.622942
H	0.556329	-2.737174	-0.625058
C	3.284721	-4.196647	-2.044031

H	5.247143	-3.312834	-2.124443
H	1.233000	-4.808965	-1.818387
H	3.581220	-5.100897	-2.567178
C	3.436357	0.990610	0.112596
C	3.289003	2.164853	-0.639902
C	4.641018	0.795615	0.807107
C	4.313902	3.108149	-0.707000
H	2.357522	2.345679	-1.169974
C	5.668891	1.733418	0.741733
H	4.781809	-0.100729	1.408176
C	5.505465	2.891574	-0.018219
H	4.181216	4.012630	-1.293048
H	6.593910	1.564598	1.284711
H	6.304574	3.625092	-0.069297
O	0.758217	0.542014	-0.440785
C	-0.540688	-0.028911	-0.179571
H	-1.049576	-0.081045	-1.161389
O	-0.472494	-1.137536	0.508724
N	-1.324475	1.070063	0.577613
H	-2.387822	0.220934	1.102569
O	1.921834	-0.526631	1.871804
C	1.252198	-1.548579	2.427657
H	1.256437	-2.473356	1.834765
O	0.755629	-1.464468	3.520843
N	-2.978880	-0.680273	1.554337
H	-2.190427	-1.343504	1.578676
C	-0.626481	1.459726	1.807964
H	-0.429062	0.551778	2.382761
H	0.329151	1.954083	1.607100
H	-1.265167	2.128501	2.390210
C	-1.754902	2.145738	-0.262653
C	-1.229326	3.437066	-0.168312
C	-2.746625	1.879358	-1.216520
C	-1.691857	4.442794	-1.016777
H	-0.462429	3.667402	0.562450
C	-3.193707	2.884619	-2.066873
H	-3.170418	0.879818	-1.278200
C	-2.669804	4.173725	-1.970019
H	-1.277620	5.442585	-0.929505
H	-3.963682	2.661849	-2.799061
H	-3.025467	4.960416	-2.627489
C	-3.475045	-0.369877	2.909661
H	-4.042987	0.560253	2.887402
H	-4.102740	-1.183111	3.277962
H	-2.609445	-0.246386	3.560502
C	-3.942859	-1.122342	0.566809
C	-5.283273	-0.759089	0.650887
C	-3.461725	-1.858406	-0.516924
C	-6.153220	-1.146701	-0.367734
H	-5.654017	-0.183024	1.491543
C	-4.341536	-2.236982	-1.526275
H	-2.405746	-2.115964	-0.549640
C	-5.688472	-1.881830	-1.455163
H	-7.200358	-0.868669	-0.305508
H	-3.972233	-2.812504	-2.369032
H	-6.372402	-2.178847	-2.243426

IM12-1a

M06-2X/BS1 SCF energy in solution :
-1784.443837 a.u.
M06-2X/BS2 SCF energy in solution:
-1784.867931 a.u.
M06-2X/BS2 Free energy in solution:
-1784.398692 a.u.

Si	-0.476474	0.972251	-0.084119
C	0.093820	2.679082	-0.688642
C	0.466734	3.691333	0.213204
C	0.130926	2.991798	-2.056382
C	0.859703	4.953431	-0.226947
H	0.450919	3.491211	1.282120
C	0.539072	4.247186	-2.507800
H	-0.171929	2.235530	-2.774604
C	0.903017	5.232530	-1.592655
H	1.137095	5.717738	0.493216
H	0.568254	4.458057	-3.573051
H	1.216569	6.212732	-1.939666
C	-2.124566	0.999467	0.861892
C	-3.333691	0.832307	0.163958
C	-2.210542	1.212879	2.248318
C	-4.565668	0.860671	0.815805
H	-3.300310	0.647604	-0.905229
C	-3.438939	1.240217	2.908310
H	-1.299675	1.345503	2.822111
C	-4.621869	1.061441	2.194092
H	-5.481652	0.717093	0.249150
H	-3.471702	1.398842	3.982596
H	-5.579179	1.077643	2.706975

O	-1.153250	0.395282	-1.652534
C	-0.230880	-0.628047	-1.758437
H	0.427305	-0.517187	-2.633195
O	0.497432	-0.427800	-0.562628
N	-0.786383	-1.974735	-1.839644
H	1.831341	-2.990959	0.813065
C	-1.840757	-2.249527	-0.922424
C	-3.158950	-2.466344	-1.346523
C	-1.563814	-2.328529	0.451122
C	-4.168711	-2.728990	-0.422465
H	-3.407788	-2.419048	-2.400948
C	-2.579098	-2.573964	1.370742
H	-0.548201	-2.192088	0.802680
C	-3.889836	-2.774000	0.941543
H	-5.183112	-2.888644	-0.776122
H	-2.338447	-2.617194	2.428904
H	-4.680556	-2.969314	1.658843
C	-1.040852	-2.354164	-3.226718
H	-0.102031	-2.295333	-3.783340
H	-1.395439	-3.386408	-3.263389
H	-1.778598	-1.712153	-3.728864
O	0.478351	0.997075	1.454402
C	1.786020	1.104619	1.466630
H	2.247892	1.280380	0.477362
O	2.462888	1.035715	2.471308
N	2.156774	-2.438849	0.012600
H	1.444561	-1.662632	-0.147634
C	2.196355	-3.316086	-1.194675
H	1.199701	-3.729554	-1.341975
H	2.479724	-2.705616	-2.052103
H	2.931460	-4.102408	-1.027461
C	3.441000	-1.821246	0.340063
C	4.172232	-2.293873	1.420439
C	3.886123	-0.768948	-0.451678
C	5.387973	-1.684052	1.722322
H	3.791863	-3.118134	2.016772
C	5.102848	-0.168946	-0.139732
H	3.284466	-0.419600	-1.286289
C	5.851237	-0.624321	0.945409
H	5.969481	-2.038085	2.566855
H	5.462202	0.658025	-0.742925
H	6.796996	-0.150123	1.186116

TS13-1a

M06-2X/BS1 SCF energy in solution :
-1784.432713 a.u.
M06-2X/BS2 SCF energy in solution:
-1784.854798 a.u.
M06-2X/BS2 Free energy in solution:
-1784.387575 a.u.

Si	-0.737449	0.972625	0.019713
C	-1.074674	2.745812	-0.533178
C	-1.536743	3.690836	0.398831
C	-0.919400	3.165501	-1.862057
C	-1.835158	4.997676	0.021030
H	-1.663128	3.400334	1.439617
C	-1.194564	4.478428	-2.244273
H	-0.585843	2.451886	-2.609629
C	-1.657328	5.395907	-1.303740
H	-2.200636	5.706306	0.758251
H	-1.055090	4.782418	-3.277549
H	-1.881153	6.416233	-1.600567
C	-2.198849	0.076381	0.800285
C	-3.306819	-0.259380	0.004367
C	-2.247818	-0.262563	2.161763
C	-4.418881	-0.901882	0.543739
H	-3.289117	-0.029303	-1.057562
C	-3.348413	-0.926807	2.702628
H	-1.411713	-0.009540	2.805340
C	-4.438347	-1.245077	1.895316
H	-5.263928	-1.149110	-0.092586
H	-3.356418	-1.191537	3.756010
H	-5.298481	-1.757754	2.315899
O	-0.968442	0.261315	-1.679766
C	0.197555	-0.337846	-1.860468
H	0.955356	0.158339	-2.473165
O	0.777339	0.179840	-0.326371
N	0.228454	-1.696589	-1.990101
H	2.393557	0.100986	2.109527
C	-0.827426	-2.465004	-1.391370
C	-1.916251	-2.839198	-2.175662
C	-0.763860	-2.836512	-0.049373
C	-2.948594	-3.586132	-1.612847
H	-1.944720	-2.536800	-3.217824
C	-1.798655	-3.582583	0.511156
H	0.083512	-2.523147	0.553368
C	-2.889907	-3.959575	-0.270301

H	-3.799351	-3.874843	-2.222030
H	-1.755009	-3.861452	1.559529
H	-3.697409	-4.537375	0.168614
C	1.554397	-2.305662	-2.004658
H	1.952536	-2.418225	-0.986236
H	1.501147	-3.287280	-2.477639
H	2.236188	-1.670041	-2.575069
O	-0.018951	1.407467	1.634308
C	1.013900	2.200287	1.763843
H	1.085336	3.013810	1.020375
O	1.853223	2.075730	2.638726
N	2.495670	-0.665477	1.439386
H	1.422674	-0.241110	0.378879
C	2.220954	-1.942245	2.103526
H	1.279015	-1.843853	2.646336
H	2.114403	-2.739248	1.364396
H	3.015670	-2.218164	2.805756
C	3.733219	-0.537308	0.743170
C	4.159513	0.749549	0.389649
C	4.487882	-1.645366	0.351721
C	5.326545	0.921622	-0.343852
H	3.574824	1.608421	0.708426
C	5.654790	-1.460176	-0.391077
H	4.178106	-2.648779	0.622197
C	6.080652	-0.183300	-0.742717
H	5.648045	1.924994	-0.605646
H	6.234459	-2.328595	-0.688686
H	6.990922	-0.046970	-1.317166

TS14-1a

M06-2X/BS1 SCF energy in solution :
-1671.136062 a.u.
M06-2X/BS2 SCF energy in solution:
-1671.525324 a.u.
M06-2X/BS2 Free energy in solution:
-1671.070279 a.u.

Si	2.077509	0.260166	-0.387717
C	3.520594	1.457230	-0.359713
C	4.705993	1.192578	-1.063741
C	3.427648	2.668537	0.340618
C	5.759277	2.104840	-1.071779
H	4.814362	0.255744	-1.607789
C	4.476300	3.587412	0.336306
H	2.522399	2.890476	0.901515
C	5.643678	3.305446	-0.371177
H	6.669166	1.881175	-1.620778
H	4.385482	4.520068	0.885349
H	6.463295	4.017909	-0.374603
C	2.702986	-1.480001	-0.115065
C	3.855329	-1.774657	0.628469
C	1.957081	-2.549864	-0.631210
C	4.248219	-3.093220	0.852777
H	4.458904	-0.965023	1.033847
C	2.341082	-3.870848	-0.408164
H	1.061083	-2.340153	-1.212115
C	3.489475	-4.142559	0.334634
H	5.144227	-3.303661	1.429118
H	1.747016	-4.685863	-0.812247
H	3.794032	-5.170351	0.508784
O	1.077594	0.691733	0.878312
C	-0.164756	1.378865	0.687102
H	-0.162330	2.199780	1.432285
O	-0.438108	1.753342	-0.540041
N	-1.254671	0.433080	1.266481
H	-2.371658	1.182934	0.808745
C	-1.306923	-0.853972	0.632291
C	-1.037589	-2.038462	1.325699
C	-1.688333	-0.927988	-0.716306
C	-1.172098	-3.272699	0.690006
H	-0.731988	-2.010020	2.365178
C	-1.812465	-2.163569	-1.343796
H	-1.884638	-0.013885	-1.263594
C	-1.562871	-3.344244	-0.643714
H	-0.964378	-4.181140	1.247487
H	-2.117814	-2.201581	-2.385136
H	-1.672140	-4.306680	-1.133674
C	-1.195536	0.404292	2.730221
H	-1.320090	1.425207	3.098641
H	-2.006509	-0.210975	3.126009
H	-0.236874	0.016950	3.091970
N	-3.148988	1.910502	0.284881
H	-2.588902	2.236123	-0.512174
C	-3.428822	3.047804	1.194125
H	-2.479074	3.518966	1.452580
H	-4.081460	3.769128	0.699558
H	-3.909687	2.668138	2.096195
C	-4.323112	1.187830	-0.154900

C	-4.941339	0.316716	0.739209
C	-4.798205	1.348863	-1.451866
C	-6.058296	-0.401539	0.322916
H	-4.542843	0.201199	1.743989
C	-5.913850	0.620672	-1.860692
H	-4.295565	2.032349	-2.130364
C	-6.544643	-0.251370	-0.975768
H	-6.544016	-1.085009	1.011676
H	-6.288220	0.737078	-2.872481
H	-7.413032	-0.816788	-1.297626
O	1.210410	0.311888	-1.778550
H	0.419637	0.898245	-1.498479

TS15-1a

M06-2X/BS1 SCF energy in solution :
-1671.133312 a.u.
M06-2X/BS2 SCF energy in solution:
-1671.521644 a.u.
M06-2X/BS2 Free energy in solution:
-1671.063809 a.u.

Si	-0.284342	0.978615	0.162452
C	0.168909	2.784140	-0.177220
C	0.964123	3.491567	0.740111
C	-0.284280	3.483990	-1.308645
C	1.295437	4.831220	0.540725
H	1.336009	2.979964	1.623145
C	0.036811	4.825102	-1.512606
H	-0.890257	2.962643	-2.042897
C	0.829280	5.502939	-0.587461
H	1.916288	5.350226	1.265305
H	-0.328471	5.341305	-2.395795
H	1.082978	6.546986	-0.745995
C	-2.029526	0.740316	0.868777
C	-3.121695	1.427712	0.313613
C	-2.279557	-0.099657	1.962075
C	-4.405965	1.291386	0.832818
H	-2.961262	2.077311	-0.544502
C	-3.568734	-0.261807	2.475856
H	-1.456485	-0.645335	2.418280
C	-4.633409	0.438346	1.915142
H	-5.232523	1.842335	0.393128
H	-3.738904	-0.932347	3.313511
H	-5.635934	0.321958	2.316254
O	-1.008712	0.699249	-1.603972
C	-0.178470	-0.196055	-2.068179
H	0.608101	0.112251	-2.763669
O	0.773469	-0.182189	-0.608182
N	-0.604940	-1.480263	-2.311126
H	1.693874	-1.069144	1.771301
C	-1.630400	-2.011409	-1.467148
C	-2.931735	-1.513952	-1.571222
C	-1.343167	-3.023117	-0.549457
C	-3.934886	-2.021251	-0.752196
H	-3.139165	-0.725769	-2.286331
C	-2.355419	-3.532266	0.263910
H	-0.334829	-3.416279	-0.470323
C	-3.651214	-3.032330	0.167584
H	-4.943175	-1.626909	-0.833978
H	-2.124286	-4.320369	0.974097
H	-4.437535	-3.426190	0.803929
C	0.448019	-2.392067	-2.748901
H	1.160030	-2.620049	-1.943186
H	-0.001445	-3.321456	-3.100172
H	0.995643	-1.929972	-3.572597
N	2.083304	-1.720385	1.087282
H	1.229249	-0.916970	-0.069737
C	1.771573	-3.109131	1.411858
H	0.736245	-3.155265	1.753509
H	1.869321	-3.739354	0.523977
H	2.425255	-3.508938	2.196916
C	3.424357	-1.384415	0.785938
C	3.789387	-0.029447	0.814027
C	4.367513	-2.343109	0.406030
C	5.079840	0.352255	0.472509
H	3.043118	0.705663	1.107365
C	5.659472	-1.944561	0.059173
H	4.104016	-3.394883	0.379781
C	6.025064	-0.602763	0.090209
H	5.350026	1.403606	0.503148
H	6.383823	-2.698705	-0.233831
H	7.032349	-0.301549	-0.178018
O	0.572759	0.697926	1.643412
H	0.122086	1.059457	2.415966

TS12-1s

M06-2X/BS1 SCF energy in solution :
-2475.245100 a.u.

M06-2X/BS2 SCF energy in solution:
-2475.816780 a.u.
M06-2X/BS2 Free energy in solution:
-2475.286692 a.u.

Si	-2.972533	-0.289921	0.637068
C	-4.152863	0.207423	-0.707684
C	-5.462250	0.593890	-0.380413
C	-3.772761	0.208248	-2.060739
C	-6.362143	0.979430	-1.371746
H	-5.792659	0.592958	0.655378
C	-4.673553	0.587673	-3.052304
H	-2.763367	-0.081484	-2.339277
C	-5.967929	0.975576	-2.707885
H	-7.370203	1.277481	-1.101278
H	-4.366233	0.581783	-4.093330
H	-6.669237	1.272801	-3.481595
C	-2.101421	-1.902233	0.386584
C	-1.071152	-2.263167	1.270605
C	-2.475305	-2.806548	-0.617590
C	-0.438545	-3.498418	1.162631
H	-0.766814	-1.570063	2.051868
C	-1.838099	-4.041713	-0.732812
H	-3.272615	-2.551569	-1.311586
C	-0.823141	-4.386568	0.157466
H	0.353197	-3.766214	1.856528
H	-2.134806	-4.733428	-1.515190
H	-0.327409	-5.348441	0.067054
O	-0.891108	0.948536	-0.995039
O	-3.854991	-0.464600	2.071476
C	-1.278952	1.695633	0.085906
H	-1.920315	2.517045	-0.250536
C	-4.216359	0.529251	2.900451
H	-3.854515	1.526139	2.603931
O	-1.916601	0.939962	1.063509
O	-4.873042	0.326936	3.884433
N	-0.043728	2.276424	0.743206
H	0.636794	1.334500	0.769451
Si	2.372655	-0.844976	0.358587
C	4.010509	0.061030	0.428384
C	4.578501	0.598194	-0.738757
C	4.664569	0.294341	1.646987
C	5.752553	1.347425	-0.690553
H	4.098845	0.428393	-1.701515
C	5.839839	1.042145	1.702123
H	4.256338	-0.114266	2.569208
C	6.383944	1.570335	0.532729
H	6.176462	1.753267	-1.604204
H	6.332549	1.210408	2.654978
H	7.300082	2.151664	0.573658
C	2.312417	-1.971488	-1.129609
C	1.086021	-2.241264	-1.754435
C	3.467246	-2.576234	-1.649612
C	1.011193	-3.088886	-2.858260
H	0.171791	-1.794957	-1.365338
C	3.399624	-3.424835	-2.752748
H	4.434407	-2.378670	-1.191871
C	2.169898	-3.680228	-3.358985
H	0.050810	-3.287621	-3.325224
H	4.303487	-3.883462	-3.142128
H	2.115559	-4.339446	-4.220183
O	1.079946	0.096998	0.456408
H	-0.147943	0.368098	-0.669605
O	2.413075	-1.898340	1.718641
C	1.799055	-1.639068	2.869797
H	1.410718	-0.611363	2.962004
O	1.677434	-2.464074	3.741238
C	-0.292762	2.782684	2.117979
H	0.621549	3.262519	2.465646
H	-1.114372	3.502222	2.113153
H	-0.540884	1.939381	2.759308
C	0.571463	3.285005	-0.122464
C	-0.074977	4.496759	-0.353724
C	1.809956	3.009577	-0.690157
C	0.534744	5.442621	-1.173582
H	-1.039302	4.704372	0.100682
C	2.413676	3.964759	-1.504453
H	2.296259	2.061501	-0.490001
C	1.777191	5.179165	-1.749185
H	0.036490	6.388314	-1.359719
H	3.382961	3.753862	-1.945360
H	2.247934	5.921448	-2.385527

TS13-1s

M06-2X/BS1 SCF energy in solution :
-2475.244943 a.u.
M06-2X/BS2 SCF energy in solution:
-2475.815913 a.u.

M06-2X/BS2 Free energy in solution:
-2475.285254 a.u.

Si	1.695466	1.432195	0.439432
C	3.371585	0.719246	0.025748
C	4.480602	0.839593	0.875815
C	3.528868	0.000454	-1.170030
C	5.700453	0.244128	0.554180
H	4.386025	1.391159	1.807691
C	4.744569	-0.595120	-1.497792
H	2.684251	-0.114780	-1.846882
C	5.831424	-0.477020	-0.631079
H	6.545711	0.340264	1.229071
H	4.843650	-1.156811	-2.422019
H	6.778183	-0.947158	-0.880010
C	1.309201	2.997533	-0.499536
C	0.317201	2.998184	-1.489503
C	1.992324	4.194421	-0.238603
C	0.009488	4.158980	-2.197941
H	-0.222034	2.077160	-1.704623
C	1.691955	5.356874	-0.945018
H	2.763557	4.223200	0.529107
C	0.699431	5.338894	-1.925297
H	-0.773066	4.142891	-2.950187
H	2.227069	6.277079	-0.730573
H	0.462794	6.245489	-2.474118
O	-0.187035	-0.460701	2.595757
O	1.930925	1.860831	2.090912
C	0.383656	-1.101092	1.598337
H	-0.249011	-1.608161	0.879071
C	0.926538	2.345335	2.818787
H	1.231429	2.522674	3.860402
O	0.507623	0.349281	0.260942
O	-0.183591	2.569551	2.401565
N	1.602652	-1.596659	1.806274
H	-1.026087	-0.070270	2.257168
Si	-2.878282	-0.074357	-0.326764
C	-4.505908	-0.418361	0.498484
C	-4.845566	0.237824	1.691365
C	-5.429643	-1.314551	-0.060414
C	-6.074451	0.008598	2.306300
H	-4.139915	0.930212	2.143843
C	-6.659266	-1.546101	0.551909
H	-5.185112	-1.844559	-0.978752
C	-6.981404	-0.883364	1.735668
H	-6.323846	0.522468	3.229560
H	-7.363707	-2.243691	0.109635
H	-7.938515	-1.064313	2.215492
C	-2.179913	-1.564540	-1.193164
C	-1.534368	-1.462181	-2.435005
C	-2.251307	-2.825721	-0.578590
C	-0.979334	-2.585868	-3.045163
H	-1.473819	-0.498572	-2.934484
C	-1.692042	-3.950098	-1.182687
H	-2.755851	-2.934996	0.379901
C	-1.059438	-3.829690	-2.419180
H	-0.487178	-2.492848	-4.008605
H	-1.756065	-4.917651	-0.694724
H	-0.627596	-4.705198	-2.894915
O	-1.880659	0.594204	0.786405
H	-0.879426	0.663908	0.486104
O	-3.090713	1.054794	-1.589246
C	-3.010700	2.384332	-1.444407
H	-2.725156	2.716628	-0.433775
O	-3.230628	3.140362	-2.353584
C	2.377260	-1.206957	2.987253
H	2.534116	-2.077482	3.628032
H	3.341189	-0.801113	2.673576
H	1.829719	-0.449440	3.540582
C	2.213190	-2.416485	0.812984
C	3.421671	-3.054865	1.107503
C	1.643221	-2.570019	-0.458914
C	4.041985	-3.850816	0.146984
H	3.884883	-2.943886	2.080066
C	2.270147	-3.378344	-1.401227
H	0.745839	-2.031720	-0.747039
C	3.469776	-4.024174	-1.109075
H	4.979979	-4.338022	0.393690
H	1.818482	-3.481761	-2.383021
H	3.955787	-4.644569	-1.854703

TS14-1s

M06-2X/BS1 SCF energy in solution :
-2361.955659 a.u.
M06-2X/BS2 SCF energy in solution:
-2362.493921 a.u.
M06-2X/BS2 Free energy in solution:
-2361.972475 a.u.

Si	3.182406	-0.427513	-1.159348
C	4.358150	0.178455	0.146084
C	5.331764	1.139975	-0.167229
C	4.285344	-0.295487	1.463901
C	6.205030	1.615433	0.808494
H	5.413482	1.514862	-1.184644
C	5.158251	0.176473	2.441968
H	3.542621	-1.044457	1.729637
C	6.117530	1.133232	2.113903
H	6.954307	2.357910	0.551551
H	5.092081	-0.201609	3.457457
H	6.798867	1.501520	2.874901
C	2.468583	-2.107774	-0.805042
C	1.085641	-2.292002	-0.668574
C	3.308259	-3.228130	-0.693848
C	0.550733	-3.560579	-0.444141
H	0.426363	-1.428711	-0.726863
C	2.778464	-4.495043	-0.462616
H	4.386681	-3.114028	-0.786736
C	1.398090	-4.662019	-0.342740
H	-0.525058	-3.684589	-0.349134
H	3.438813	-5.352588	-0.378029
H	0.985457	-5.651057	-0.167887
O	1.098967	0.947455	0.787599
C	1.512342	1.532323	-0.364552
H	2.300914	2.264641	-0.158882
O	1.889685	0.635377	-1.350958
N	0.324721	2.297854	-0.969440
H	-0.440235	1.546834	-0.947680
Si	-2.346464	-0.722316	-0.175475
C	-4.005702	0.114147	-0.409163
C	-4.606174	0.796346	0.661728
C	-4.653183	0.137847	-1.652912
C	-5.806461	1.484325	0.495584
H	-4.133799	0.786927	1.642956
C	-5.854655	0.823878	-1.825750
H	-4.217813	-0.389090	-2.499546
C	-6.431803	1.497948	-0.751009
H	-6.256529	2.004444	1.335821
H	-6.342325	0.829663	-2.795971
H	-7.368691	2.030754	-0.883240
C	-2.361638	-1.769173	1.374265
C	-1.207381	-1.884914	2.162829
C	-3.506488	-2.477938	1.769381
C	-1.191263	-2.687235	3.302510
H	-0.305475	-1.346169	1.879763
C	-3.497251	-3.283452	2.906434
H	-4.421372	-2.396923	1.185397
C	-2.337459	-3.388535	3.673620
H	-0.287950	-2.765119	3.899948
H	-4.392176	-3.825924	3.196110
H	-2.328174	-4.013874	4.561362
O	-1.087552	0.255668	-0.257462
H	0.220019	0.491368	0.559635
O	-2.284958	-1.868499	-1.463403
C	-1.577870	-1.693152	-2.578186
H	-0.996318	-0.756390	-2.600735
O	-1.574592	-2.490446	-3.481980
C	0.566074	2.756449	-2.365072
H	-0.299447	3.337963	-2.680008
H	1.466273	3.371911	-2.393291
H	0.689769	1.882242	-2.999238
C	-0.102539	3.388884	-0.085776
C	0.732450	4.484558	0.110195
C	-1.349620	3.303133	0.521983
C	0.302198	5.515480	0.941325
H	1.701674	4.537947	-0.376249
C	-1.769651	4.344251	1.346568
H	-1.973084	2.431104	0.351356
C	-0.946212	5.447672	1.558290
H	0.945231	6.374062	1.103608
H	-2.742916	4.287249	1.823116
H	-1.276325	6.255975	2.202610
O	4.041743	-0.403867	-2.569281
H	3.581252	-0.701965	-3.364348

TS15-1s

M06-2X/BS1 SCF energy in solution :
-2361.957582 a.u.
M06-2X/BS2 SCF energy in solution:
-2362.493775 a.u.
M06-2X/BS2 Free energy in solution:
-2361.970980 a.u.

Si	1.453838	-0.753619	-0.815438
C	3.307841	-0.943995	-0.679753
C	4.181728	-0.633198	-1.732161

C	3.864253	-1.367707	0.537646
C	5.564207	-0.721068	-1.568392
H	3.779214	-0.301932	-2.685848
C	5.243867	-1.456139	0.708286
H	3.212855	-1.609827	1.375015
C	6.096047	-1.128198	-0.346292
H	6.224824	-0.468168	-2.392238
H	5.654041	-1.772822	1.662686
H	7.172206	-1.189269	-0.214446
C	0.548203	-2.363260	-1.084387
C	0.138796	-3.125028	0.020495
C	0.255956	-2.844369	-2.368917
C	-0.534556	-4.332728	-0.150571
H	0.335613	-2.765227	1.028267
C	-0.427247	-4.046418	-2.545592
H	0.555476	-2.272546	-3.244740
C	-0.821043	-4.792302	-1.435521
H	-0.843181	-4.910313	0.715701
H	-0.653127	-4.400174	-3.546979
H	-1.352611	-5.729247	-1.571862
O	0.294539	2.426789	-0.453469
O	1.345880	0.140426	-2.292887
C	1.165859	2.020490	0.441294
H	0.858827	1.982245	1.479366
C	0.205923	0.586277	-2.789346
H	0.361656	1.264687	-3.638929
O	0.830295	0.076472	0.419546
O	-0.898293	0.291343	-2.383240
N	2.459271	2.241768	0.190958
H	-0.595213	2.060741	-0.175270
Si	-2.926647	0.108664	0.145905
C	-3.994521	1.374883	-0.724951
C	-3.419612	2.363027	-1.539505
C	-5.392977	1.346893	-0.613321
C	-4.209396	3.291731	-2.213467
H	-2.340001	2.400034	-1.662476
C	-6.189656	2.273093	-1.284906
H	-5.870283	0.596285	0.012904
C	-5.597204	3.248450	-2.084557
H	-3.744550	4.048895	-2.837767
H	-7.269957	2.235873	-1.182033
H	-6.214663	3.973317	-2.606550
C	-3.797671	-0.554587	1.659892
C	-3.773435	-1.924709	1.959179
C	-4.450221	0.307377	2.555331
C	-4.379473	-2.418627	3.113597
H	-3.274687	-2.609973	1.278251
C	-5.056485	-0.179812	3.711157
H	-4.494030	1.374393	2.344108
C	-5.020875	-1.545741	3.990711
H	-4.352425	-3.482619	3.329126
H	-5.557381	0.502057	4.391735
H	-5.493693	-1.928853	4.890097
O	-1.525926	0.904588	0.546763
H	-0.660480	0.357323	0.560753
C	2.898920	2.692172	-1.130978
H	3.310416	3.701691	-1.058676
H	3.660461	2.011123	-1.516422
H	2.048225	2.702151	-1.805981
C	3.435210	1.944182	1.183730
C	4.760134	2.342272	0.981884
C	3.099268	1.232978	2.344604
C	5.729298	2.047663	1.938477
H	5.046772	2.884291	0.089115
C	4.074733	0.963903	3.298035
H	2.099525	0.842226	2.489816
C	5.394725	1.366520	3.104541
H	6.753270	2.362112	1.763149
H	3.798048	0.411136	4.190334
H	6.152670	1.142334	3.847695
O	-2.595823	-1.232296	-0.759013
H	-1.912360	-1.077660	-1.430213
